# Supplementary material for: A boron-transfer mechanism mediating the thermally induced revival of frustrated carbene–borane pairs from their shelf-stable adducts
Source: Commun Chem. 2021 Sep 27;4:137. doi: 10.1038/s42004-021-00576-1 (PMC9814311; doi:10.1038/s42004-021-00576-1)

# Supplementary Materials

## A boron-transfer mechanism mediating the thermally induced revival of frustrated carbene–borane pairs from their shelf-stable adducts

Yoichi Hoshimoto,[*1] Mahiro Sakuraba,[1] Takuya Kinoshita,[1] Masaki Ohbo,[2] Manussada Ratanasak,[2] Jun-ya Hasegawa,[*2] and Sensuke Ogoshi[*1]

[1]*Department of Applied Chemistry, Faculty of Engineering, Osaka University, Suita, Osaka 565-0871, Japan*
[2]*Institute for Catalysis Hokkaido University, Kita 21, Nishi 10, Kitaku, Sapporo 001-0021, Japan*

E-mail: hoshimoto@chem.eng.osaka-u.ac.jp
ogoshi@chem.eng.osaka-u.ac.jp
hasegawa@cat.hokudai.ac.jp

## Supplementary Methods

[1] General Considerations
[2] Materials
[3] Synthesis of 3aB$^2$, 5aB$^2$, and [1a–H][HO(B$^2$)$_2$]
[4] Reaction between 1a and B(*p*-$HC_6F_4$)$_3$ Giving 2aB$^2$
[5] NMR Experiments
[6] Variable Temperature NMR Experiments
[7] Kinetic Studies
[8] Theoretical Studies

## Supplementary References

## NMR Spectra

# Supplementary Methods

## [1] General Considerations

Unless otherwise noted, all manipulations were conducted under a nitrogen atmosphere using standard Schlenk or dry box techniques. $^{1}$H, $^{11}$B, $^{13}$C, $^{19}$F, and $^{31}$P NMR spectra were recorded on a Bruker AVANCE III 400 or JEOL JNM-400 spectrometers at 25 °C. The chemical shifts in the $^{1}$H NMR spectra were recorded relative to $Me_4Si$ or residual protonated solvent ($CDHCl_2$ ($\delta$ 5.32) or DCE-$d_4$ ($\delta$ 3.75)). The chemical shifts in the $^{11}$B NMR spectra were recorded relative to $BF_3$. The chemical shifts in the $^{13}$C spectra were recorded relative to $Me_4Si$ or deuterated solvent ($CD_2Cl_2$ ($\delta$ 53.84)). The chemical sifts in the $^{19}$F NMR spectra were recorded relative to α,α,α-trifluorotoluene ($\delta$ −65.64). The chemical shifts in the $^{31}$P NMR spectra were recorded relative to 85% $H_3PO_4$ as an external standard. Assignment of the resonances in $^{1}$H and $^{13}$C NMR spectra was based on $^{1}$H–$^{1}$H COSY, HMQC and HMBC experiments. Elementary analyses were performed at Instrumental Analysis Center, Faculty of Engineering, Osaka University. ESI-MS analyses were performed with a Bruker Daltonics micrOTOF mass spectrometer. X-ray crystal data were collected with Rigaku XtaLAB Synergy equipping with the HyPix-6000HE detector.

## [2] Materials

All commercially available reagents were used as received. Unless otherwise noted, toluene, hexane, and mesitylene were distilled from sodium benzophenone ketyl prior to use. $CH_2Cl_2$ and 1,2-dichloroethane were distilled over $CaH_2$ prior to use. $CD_2Cl_2$ and DCE-$d_4$ were distilled over $CaH_2$ and stored over molecular sieves (4 Å). PoxIm (**1a**)[1] and B($p$-$HC_6F_4$)$_3$ (**B$^2$**)[2–3] were furnished by the known procedure.

Metrical data for the solid state structures are available from Cambridge Crystallographic Data Centre: CCDC2072358 (**3aB$^2$**), 2072359 (**5aB$^2$**), 2072360 (**2aB$^2$**), 2072638 ([**1a–H][HO(B$^2$)$_2$**]).

## [3] Synthesis of 3aB$^2$, 5aB$^2$, and [1a–H][HO(B$^2$)$_2$]

## 3-1. Synthesis of 3aB$^2$

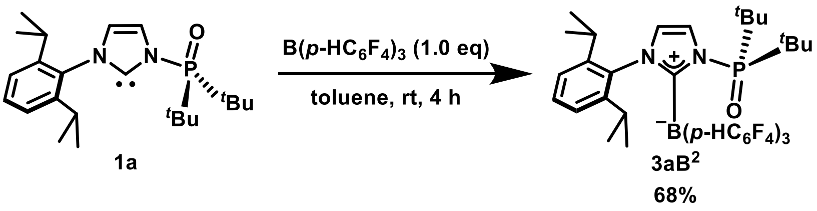


PoxIm **1a** (154.8 mg, 0.40 mmol) and B($p$-$HC_6F_4$)$_3$ (**B$^2$**) (183.4 mg, 0.40 mmol) were mixed in toluene (10 mL) at room temperature to furnish the yellow solution. Stirring this mixture for 4 h resulted into the precipitation of a white solid, which was collected via removal of a supernatant solution. The obtained solid was washed with hexane (5 mL) and dried *in vacuo* to afford **3aB$^2$** as a white solid (230.2 mg, 0.27 mmol, 68%). A single crystal suitable for X-ray diffraction analysis was prepared by recrystallization from $CH_2Cl_2$/hexane at –30 °C (Supplementary Figure 1). **$^{1}$H NMR** (400 MHz, $CD_2Cl_2$): $\delta$ 7.43 (m, 1H, Im-*H*), 7.25–7.17 (m, 2H, $C_{Dipp}$-*H*), 7.10 (s, 1H, Im-*H*), 6.88–6.84 (m, 2H, $C_{Dipp}$-*H* and $C_{B2}$-*H* are overlapped), 6.76 (tt, $^{3}J_{H,F}$ = 8.2 Hz, $^{4}J_{H,F}$ = 8.0 Hz, 1H, $C_{B2}$-*H*), 6.43 (tt, $^{3}J_{H,F}$ = 8.2 Hz, $^{4}J_{H,F}$ = 8.0 Hz, 1H, $C_{B2}$-

*H*), 3.02 (brs, 1H, C*H*($CH_3$)$_2$), 2.53 (sept, $J$ = 6.7 Hz, 1H, C*H*($CH_3$)$_2$), 1.45 (d, $^3J_{H,P}$ = 15.6 Hz, 9H, $^t$Bu-*H*), 1.30 (d, $J$ = 6.8 Hz, 3H, CH(C*H*$_3$)$_2$), 1.08 (d, $J$ = 6.8 Hz, 3H, CH(C*H*$_3$)$_2$), 1.08 (d, $^3J_{H,P}$ = 15.6 Hz, 9H, $^t$Bu-*H*), 0.98 (d, $J$ = 6.8 Hz, 3H, CH(C*H*$_3$)$_2$), 0.89 (d, $J$ = 6.8 Hz, 3H, CH(C*H*$_3$)$_2$). **$^{11}$B{$^1$H}NMR** (128 MHz, $CD_2Cl_2$): $\delta$ –14.1 (s). **$^{13}$C{$^1$H}NMR** (100 MHz, $CD_2Cl_2$): $\delta$ 148.1, 145.5, 134.7, 131.3, 128.0, 123.7, 123.6, 121.8 (d, $^2J_{C,P}$ = 7.0 Hz), 104.0 (brs), 102.3 (m), 43.3 (d, $^1J_{C,P}$ = 57.0 Hz), 41.1 (d, $^1J_{C,P}$ = 60.0 Hz), 29.1–27.3 (10C, *C*H($CH_3$)$_2$, CH(*C*H$_3$)$_2$, C(*C*H$_3$)$_3$), 21.24, 21.19. Resonances of N*C*N, and carbons at the *ipso*, *ortho*, and *meta* positions in *p*-H$C_6F_4$ groups could not be identified. **$^{19}$F NMR** (376 MHz, $CD_2Cl_2$): $\delta$ –115.8 (s, 1F), –126.3 (s, 1F), –131.6 (s, 1F), –131.9 (s, 1F), –134.3 (s, 1F), –139.0 (s, 1F), –145.4 (s, 1F), –145.8 (m, 1F), –146.2 (m, 1F), –148.5 (m, 1F), –149.7 (brs, 1F), –150.0 (m, 1F). **$^{31}$P{$^1$H}NMR** (162 MHz, $CD_2Cl_2$): $\delta$ 75.1 (s). X-ray data for **$C_{41}H_{40}BF_{12}N_2OP$** ($M$ = 846.57 g/mol): monoclinic, space group P2$_1$/c (#14), $a$ = 14.2029(3) Å, $b$ = 17.1447(3) Å, $c$ = 16.5196(3) Å, $\beta$ = 99.683(2)°, $V$ = 3965.29(13) Å$^3$, $Z$ = 4, $T$ = 143.15 K, $\mu$(Cu Kα) = 1.438 mm$^{-1}$, *Dcalc* = 1.4179 g/cm$^3$ 50366 reflections measured (7.48° ≤ 2θ ≤ 136.48°), 7243 unique ($R_{int}$ = 0.0573, $R_{sigma}$ = 0.0319) which were used in all calculations. The final $R_1$ was 0.0415 (I ≥ 2σ(I)) and $wR_2$ was 0.0937 (all data). **Anal. Calcd for $C_{41}H_{40}BF_{12}N_2OP$:** C, 58.17; H, 4.76; N, 3.31. Found: C, 58.01; H, 4.96; N, 3.38.

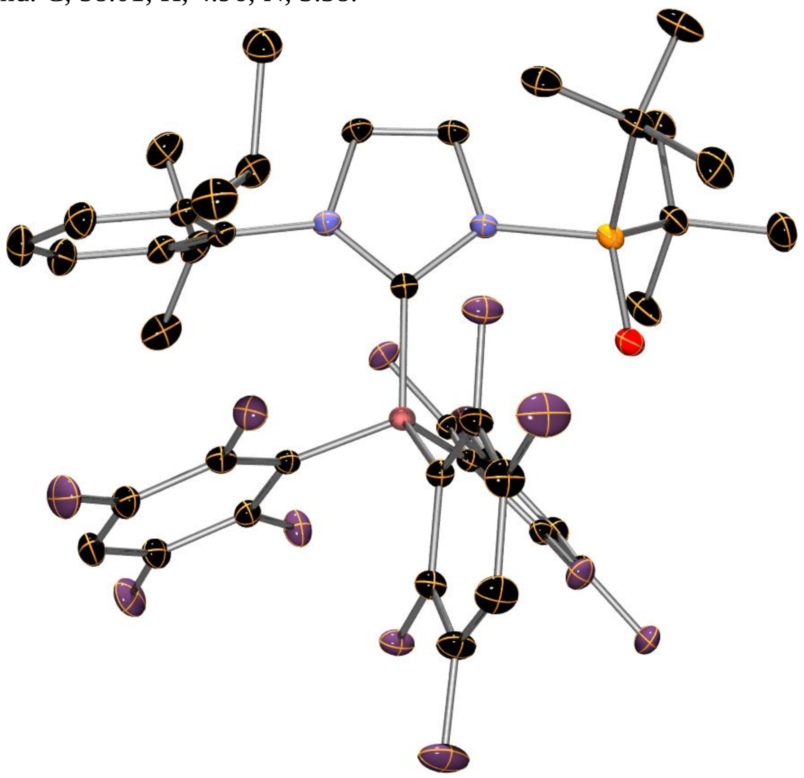

**Supplementary Figure 1.** The molecular structure of **3aB$^2$** with ellipsoids set at 30% probability, in which hydrogen atoms are omitted for clarity.

## 3-2. Synthesis of $5aB^2$

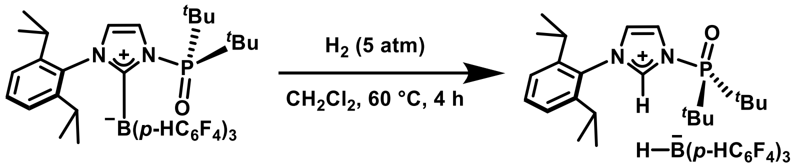


$3aB^2$ $5aB^2$ >99%

A solution of $\mathbf{3aB^2}$ (51.6 mg, 0.06 mmol) in $CH_2Cl_2$ (3 mL) was transferred into an autoclave reactor. Then, $H_2$ (5 atm) was pressurized, and the reaction mixture was stirred at 60 °C for 4 h. Then, the solvent was removed *in vacuo* to give $\mathbf{5aB^2}$ as a white solid (51.8 mg, 0.06 mmol, >99%). A single crystal suitable for X-ray diffraction analysis was prepared by recrystallization from THF/hexane at –30 °C (Supplementary Figure 2). **$^1$H NMR** (400 MHz, $CD_2Cl_2$): $\delta$ 8.81 (s, 1H, Im-*H*), 7.86 (s, 1H, Im-*H*), 7.64 (t, $J$ = 8.0 Hz, 1H, $C_{Dipp}$-*H*), 7.58 (s, 1H, Im-*H*), 7.40 (d, $J$ = 8.0 Hz, 2H, $C_{Dipp}$-*H*), 6.72–6.64 (m, 3H, $C_{B2}$-*H*), 3.70 (1:1:1:1 q, $^1J_{H,B}$ = 88.3 Hz, 1H, B-*H*), 2.17 (sept, $J$ = 6.9 Hz, 2H, C*H*($CH_3$)$_2$), 1.39 (d, $^3J_{H,P}$ = 16.4 Hz, 18H, $^t$Bu-*H*), 1.19 (d, $J$ = 6.8 Hz, 6H, CH(C$H_3$)$_2$), 1.15 (d, $J$ = 6.8 Hz, 6H, CH(C$H_3$)$_2$). **$^{11}$B{$^1$H}NMR** (128 MHz, $CD_2Cl_2$): $\delta$ –24.6 (d, $^1J_{H,B}$ = 88.3 Hz). **$^{13}$C{$^1$H}NMR** (100 MHz, $CD_2Cl_2$): $\delta$ 148.7 (dm, $^1J_{C,F}$ = 235.0 Hz), 145.6 (dm, $^1J_{C,F}$ = 242.0 Hz), 145.2, 141.4, 133.2, 129.7, 128.3, 125.5, 123.6, 103.7 (t, $^2J_{C,F}$ = 22.5 Hz), 101.0 (t, $^2J_{C,F}$ = 23.0 Hz), 38.6 (d, $^1J_{C,P}$ = 59.0 Hz), 29.5, 25.9, 24.5, 23.6. **$^{19}$F NMR** (376 MHz, $CD_2Cl_2$): $\delta$ –137.4 (s, 6F), –147.6 (m, 6F). **$^{31}$P{$^1$H}NMR** (162 MHz, $CD_2Cl_2$): $\delta$ 77.5 (s). **ESI-MS** (pos): m/z Calcd for $C_{23}H_{38}N_2OP$ [M–HBAr$_3$]$^+$ 389.2716, found 389.2726; (neg): m/z Calcd for $C_{18}H_4BF_{12}$ [M–Im]$^-$ 459.0214, found 459.0258. X-ray data for $\mathbf{C_{41}H_{42}BF_{12}N_2OP}$ ($M$ = 848.58 g/mol): monoclinic, space group P2$_1$/c (#14), $a$ = 13.4128(2) Å, $b$ = 19.2008(2) Å, $c$ = 16.4807(2) Å, $\beta$ = 110.160(2)°, $V$ = 3984.34(10) Å$^3$, $Z$ = 4, $T$ = 123.15 K, $\mu$(Cu Kα) = 1.432 mm$^{-1}$, *Dcalc* = 1.4145 g/cm$^3$, 44646 reflections measured (7.02° ≤ 2θ ≤ 136.5°), 7288 unique ($R_{int}$ = 0.0393, $R_{sigma}$ = 0.0255) which were used in all calculations. The final $R_1$ was 0.0414 (I ≥ 2σ(I)) and $wR_2$ was 0.1144 (all data).

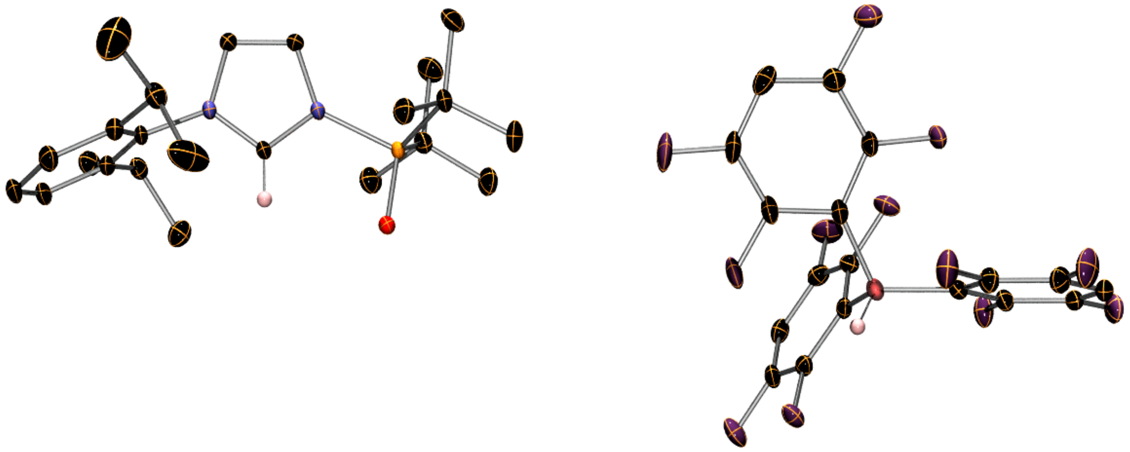

**Supplementary Figure 2.** The molecular structure of $\mathbf{5aB^2}$ with ellipsoids set at 30% probability, in which hydrogen atoms are omitted for clarity excepted description.

## 3-3. Synthesis of [1a–H][HO(B$^2$)$_2$]

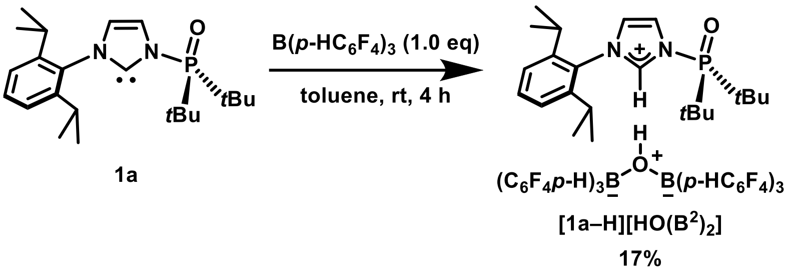


PoxIm **1a** (116.5 mg, 0.30 mmol) and **B$^2$** (136.9 mg, 0.30 mmol) were mixed in toluene (*10 mL of a commercially available super dehydrated toluene including up to 1 ppm H$_2$O*) at room temperature to furnish the yellow solution. After stirring this mixture for 4 h at room temperature, addition of hexane (5 mL) and the following decantation gave a mixture of **3aB$^2$** and **[1a–H][HO(B$^2$)$_2$]**. The resultant solid was dissolved in $CH_2Cl_2$ (2 mL) and stored at –30 °C for 2 d, which afforded a single crystal of **[1a–H][HO(B$^2$)$_2$]** (66.7 mg, 0.05 mmol, 17%). This crystal was used for the single-crystal X-ray diffraction analysis (Supplementary Figure 3). **$^1$H NMR** (400 MHz, $CD_2Cl_2$): $\delta$ 8.79 (s, 1H, Im-*H*), 7.68 (s, 1H, Im-*H*), 7.66 (t, 1H, $J$ = 8.0 Hz, C$_{Dipp}$-*H*), 7.54 (s, 1H, Im-*H*), 7.42 (d, 2H, $J$ = 8.0 Hz, C$_{Dipp}$-*H*), 6.84 (brs, 1H, O*H*), 6.76 (tt, $^3J_{H,F}$ = 8.4 Hz, $^4J_{H,F}$ = 7.2 Hz, 6H, C$_{B2}$-*H*), 2.16 (sept, $J$ = 6.8 Hz, 2H, C*H*(CH$_3$)$_2$), 1.42 (d, $^3J_{H,P}$ = 16.4 Hz, 18H, $^t$Bu-*H*), 1.21 (d, $J$ = 6.8 Hz, 6H, CH(C*H*$_3$)$_2$), 1.17 (d, $J$ = 6.8 Hz, 6H, CH(C*H*$_3$)$_2$). **$^{11}$B{$^1$H}NMR** (128 MHz, $CD_2Cl_2$): $\delta$ 0.37 (s). **$^{13}$C{$^1$H}NMR** (150 MHz, $CD_2Cl_2$): $\delta$ 148.0 (d, $^1J_{C,F}$ = 234.0 Hz), 145.6 (d, $^1J_{C,F}$ = 243 Hz), 145.1, 133.4, 129.5, 128.0, 126.8, 125.6, 123.2, 103.7 (t, $^2J_{C,F}$ = 23.3 Hz), 38.7 (d, $^1J_{C,P}$ = 58.5 Hz), 29.6, 26.0, 24.5, 23.7. Resonance of N*C*N could not be identified. **$^{19}$F NMR** (376 MHz, $CD_2Cl_2$): $\delta$ –136.8 (s, 12F), –146.2 (s, 12F,). **$^{31}$P{$^1$H}NMR** (162 MHz, $CD_2Cl_2$): $\delta$ 77.7 (s). X-ray data for **$C_{59}H_{45}B_2F_{24}N_2O_2P$** ($M$ = 1322.611 g/mol): colorless, monoclinic, space group P2$_1$/c (#14), $a$ = 12.5482(1) Å, $b$ = 29.6562(4) Å, $c$ = 17.0447(2) Å, $\beta$ = 94.211(1)°, $V$ = 6325.75(12) Å$^3$, $Z$ = 4, $T$ = 143.15 K, $\mu$(Cu Kα) = 1.542 mm$^{-1}$, *Dcalc* = 1.389 g/cm$^3$, 83587 reflections measured (7.66° ≤ 2θ ≤ 149°), 12731 unique ($R_{int}$ = 0.0566, $R_{sigma}$ = 0.0333) which were used in all calculations. The final $R_1$ was 0.0433 (I ≥ 2σ(I)) and $wR_2$ was 0.1110 (all data). Note that two severely disordered $CH_2Cl_2$ molecules are found, and thus refinements used the following solvent mask conditions: A solvent mask was calculated and 304 electrons were found in a volume of 974 (Å$^3$) in 1 void per unit cell. This is consistent with the presence of 2[$CH_2Cl_2$] per asymmetric unit which account for 336 electrons per unit cell.

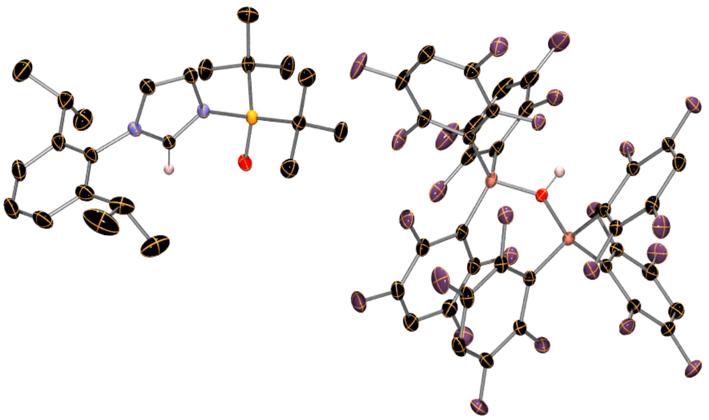

**Supplementary Figure 3.** The molecular structure of **[1a–H][HO(B$^2$)$_2$]** with ellipsoids set at

30% probability, in which hydrogen atoms are omitted for clarity.

## [4] Reaction between 1a and B($p$-HC$_6$F$_4$)$_3$ Giving 2aB$^2$

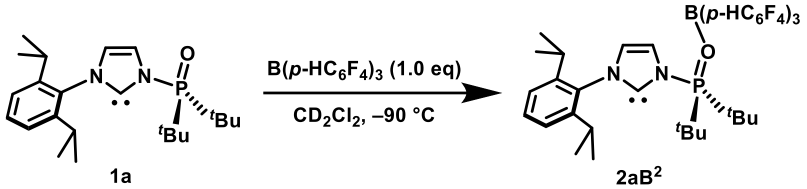


**1a** **2aB$^2$ quant**

A solution of **1a** (7.4 mg, 0.02 mmol) and **B$^2$** (9.3 mg, 0.02 mmol) in $CD_2Cl_2$ (0.5 mL) was prepared at –30 °C, and then transferred into a J. Young NMR tube. The quantitative formation of **2aB$^2$** was then confirmed at –90 °C by $^1$H, $^{13}$C, $^{19}$F, and $^{31}$P NMR analyses (Supplementary Figures 5–8). A single crystal suitable for X-ray diffraction analysis was prepared by recrystallization from toluene/hexane at –30 °C (Supplementary Figure 4). **$^1$H NMR** (400 MHz, $CD_2Cl_2$, –90 °C): $\delta$ 7.58–7.33 (m, 3H, Im-$H$, $C_{Dipp}$-$H$), 7.00–6.97 (m, 4H, Im-$H$, $C_{Dipp}$-$H$, $C_{B2}$-$H$), 6.81–6.72 (m, 1H, $C_{B2}$-$H$), 2.27–2.26 (m, 2H, C$H$($CH_3$)$_2$), 1.26–0.97 (m, 30H, tBu-$H$, CH(C$H_3$)$_2$). **$^{13}$C NMR** (100 MHz, $CD_2Cl_2$, –90 °C): $\delta$ 219.5 (d, $^2J_{C,P}$ = 32.0 Hz, N$C$N), 147.2 (d, $^1J_{C,F}$ = 215 Hz), 145.1, 144.8 (dm, $^1J_{C,F}$ = 217 Hz), 135.5, 129.2, 125.1, 123.5, 123.3, 121.8, 103.1, 38.9 (d, $^1J_{C,P}$ = 63.0 Hz), 27.7, 25.8, 23.7, 22.7. Resonances of some carbons at the *ipso*, *ortho*, *meta*, and *para* positions in B($p$-H$C_6$F$_4$)$_3$ could not be identified. **$^{19}$F NMR** (376 MHz, $CD_2Cl_2$, –90 °C): $\delta$ –129.8 (4F), –133.7 (2F), –141.7 (6F). **$^{31}$P{H}NMR** (162 MHz, $CD_2Cl_2$, –90 °C): $\delta$ 79.3 (s). X-ray data for **$C_{55}H_{57}BF_{13}N_2PO_{0.25}$** ($M$ =1030.85 g/mol): monoclinic, space group P2$_1$/c (#14), $a$ = 16.2735(2) Å, $b$ = 15.2296(2) Å, $c$ = 20.5604(2) Å, $\beta$ = 93.9330(10)°, $V$ = 5083.67(10) Å$^3$, $Z$ = 4, $T$ = 143.15 K, $\mu$(Cu K$\alpha$) = 1.222 mm$^{-1}$, $Dcalc$ = 1.3467 g/cm$^3$, 65960 reflections measured (6.7° ≤ 2θ ≤ 136.48°), 9330 unique ($R_{int}$ = 0.0346, $R_{sigma}$ = 0.0199) which were used in all calculations. The final $R_1$ was 0.0481 (I ≥ 2σ(I)) and $wR_2$ was 0.1321 (all data).

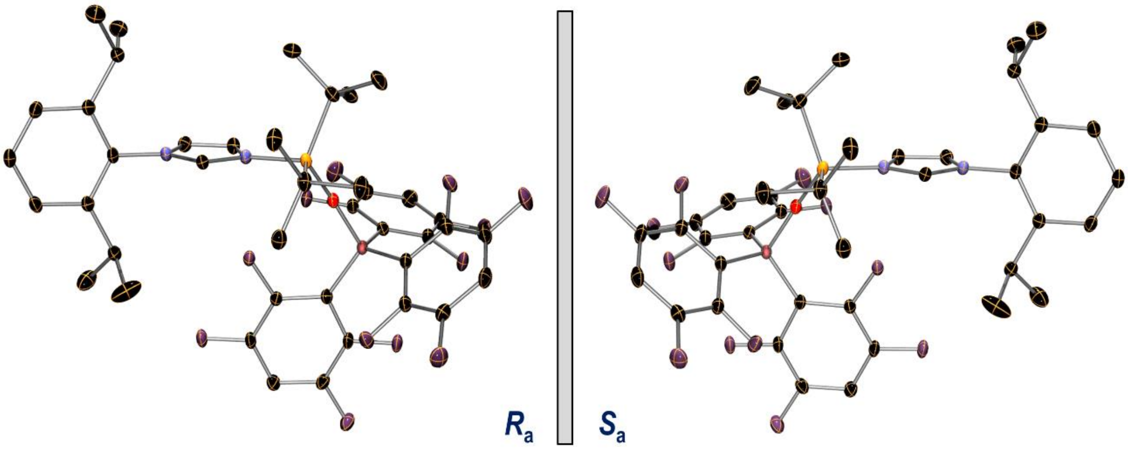


**Supplementary Figure 4.** Molecular structure of **2aB$^2$** with ellipsoids set at 30% probability, in which hydrogen atoms and solvated $C_7H_8$ molecules are omitted for clarity. In this crystalline lattice, ($R_a$)- and ($S_a$)-atropisomers are included.

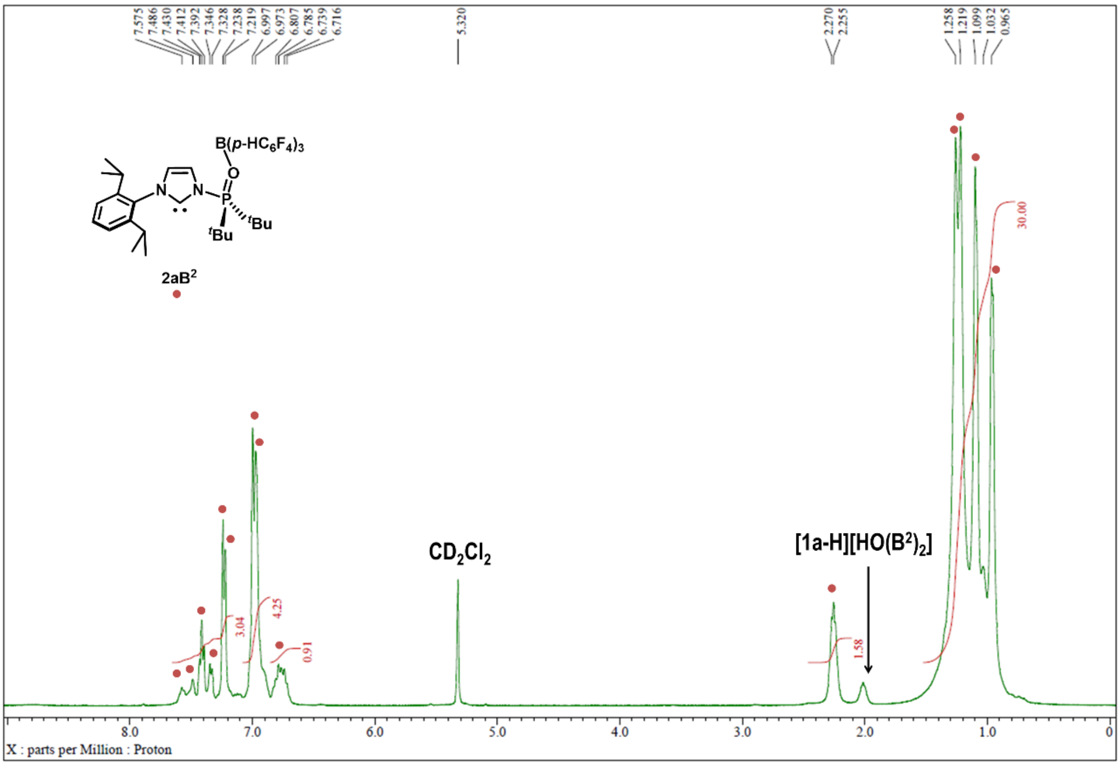


**Supplementary Figure 5.** Formation of **2aB$^2$** confirmed at –90 °C by $^1$H NMR.

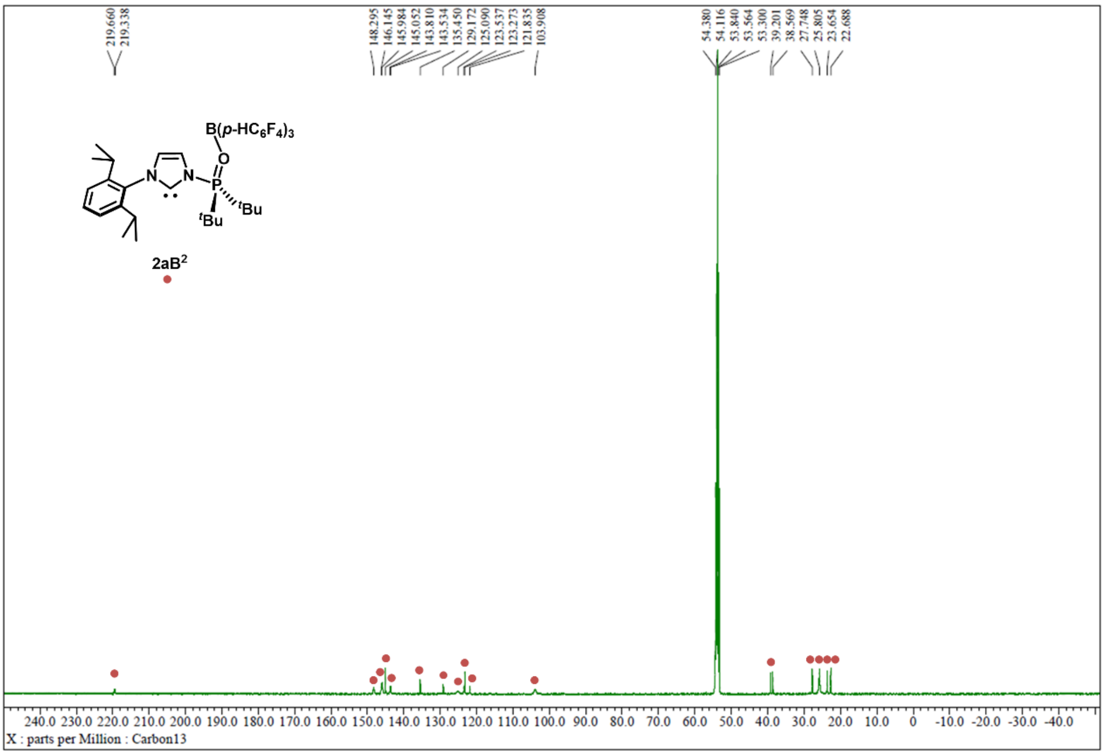


**Supplementary Figure 6.** Formation of **2aB$^2$** confirmed at –90 °C by $^{13}$C NMR.

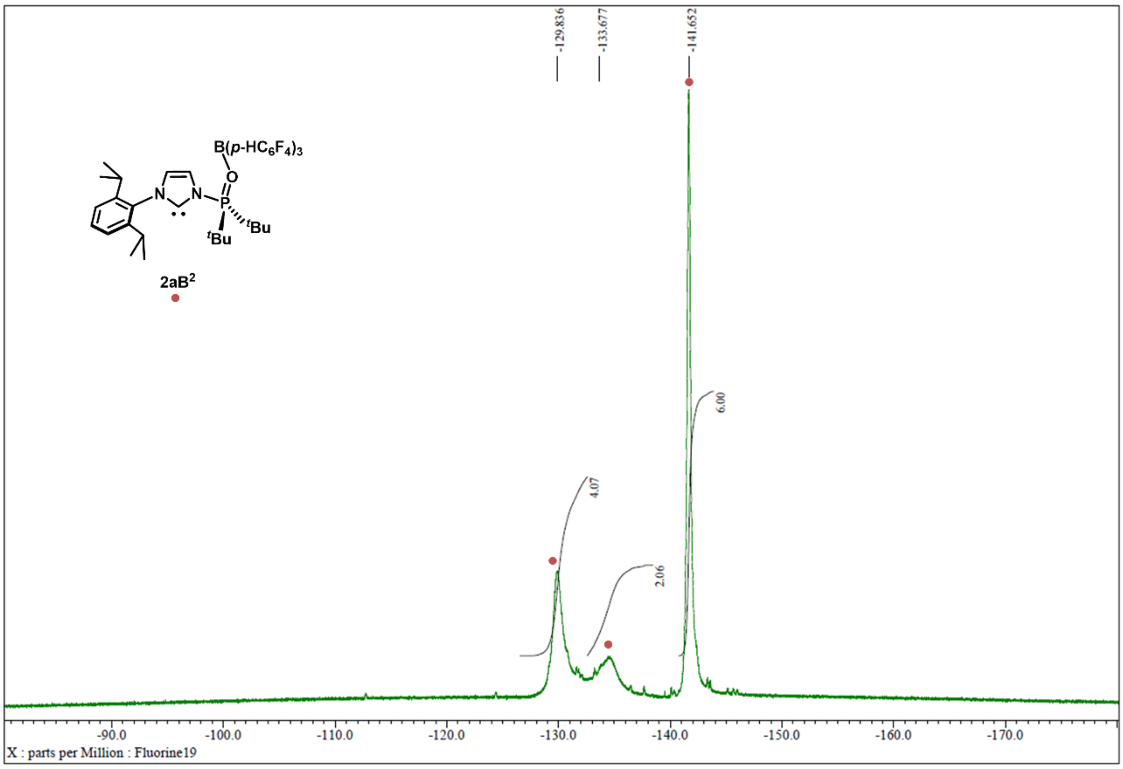


**Supplementary Figure 7.** Formation of **2aB$^2$** confirmed at –90 °C by $^{19}$F NMR.

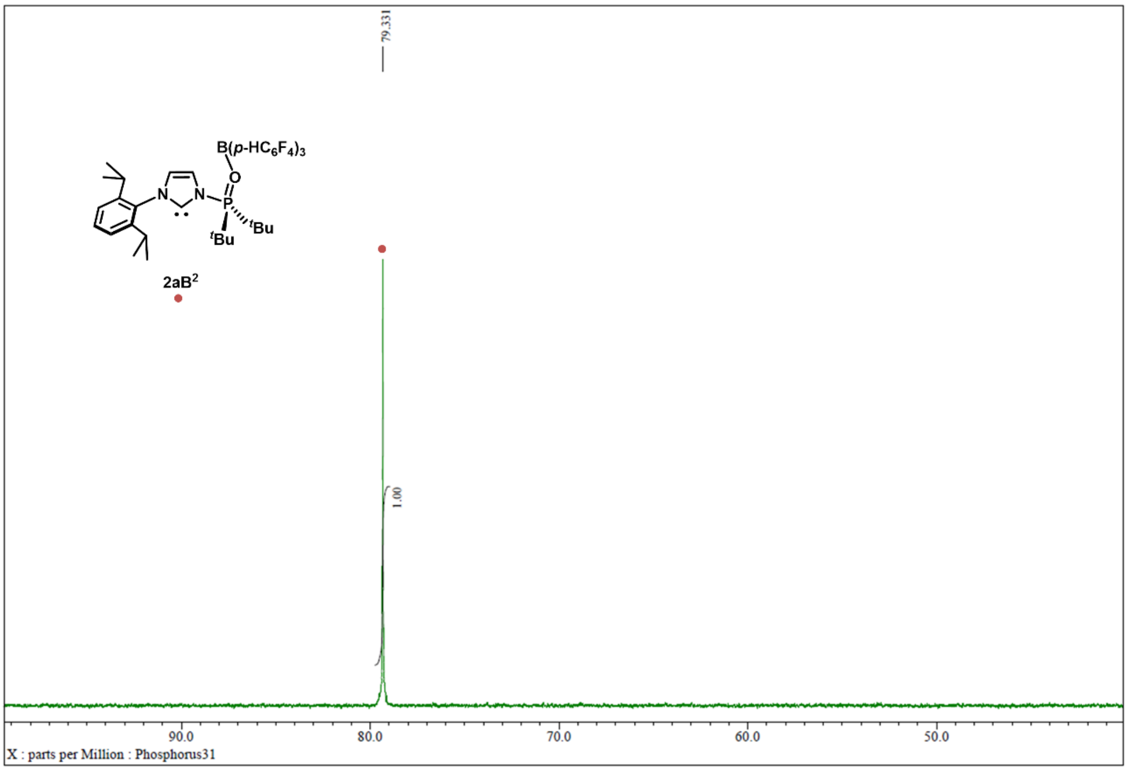


**Supplementary Figure 8.** Formation of **2aB$^2$** confirmed at –90 °C by $^{31}$P NMR.

# [5] NMR Experiments

## 5-1. Reaction between 1a and B($p$-$HC_6F_4$)$_3$ at 22 °C

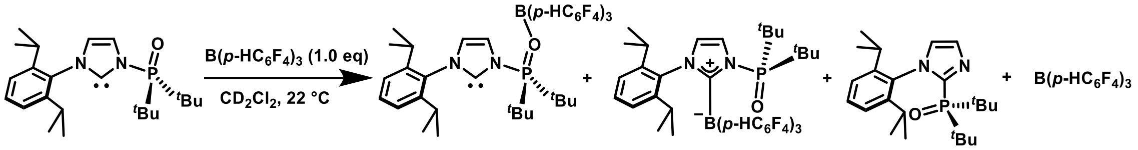


1a → 2aB$^2$ + 3aB$^2$ + 4a + B$^2$

| | 2aB$^2$ | 3aB$^2$ | 4a | B$^2$ |
|---|---|---|---|---|
| 20 min | 61% | 29% | 9% | n.d. |
| 2 h | 16% | 61% | 23% | n.d. |
| 6 h | n.d. | 75% | 25% | 12% |

PoxIm **1a** (5.0 mg, 0.01 mmol) and **B$^2$** (5.8 mg, 0.01 mmol) were mixed in $CD_2Cl_2$ (0.5 mL), and the resultant solution was transferred into a J. Young NMR tube. The reaction was monitored by $^1$H, $^{19}$F, and $^{31}$P NMR analyses conducted at 22 °C (Supplementary Figures 9–11). Note that the identification of **2aB$^2$** in $CD_2Cl_2$ at room temperature were independently conducted by $^1$H, $^{19}$F, and $^{31}$P NMR analyses shown in Supplementary Figures 24–26. Yield of products were estimated by the $^{19}$F and $^{31}$P NMR analyses.

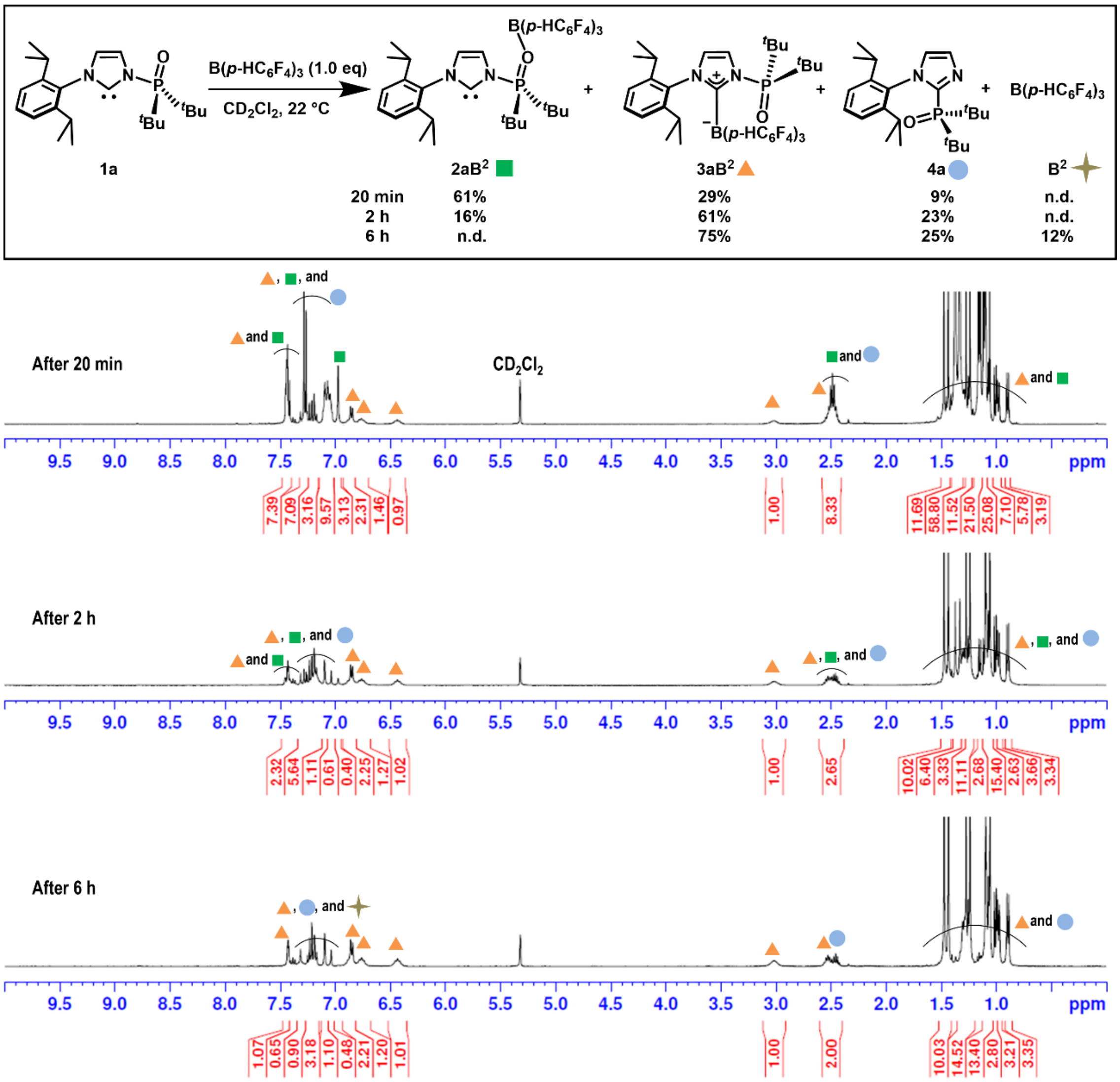


**Supplementary Figure 9.** Reaction between **1a** and **B**$^2$ at 22 °C monitored by $^1$H NMR.

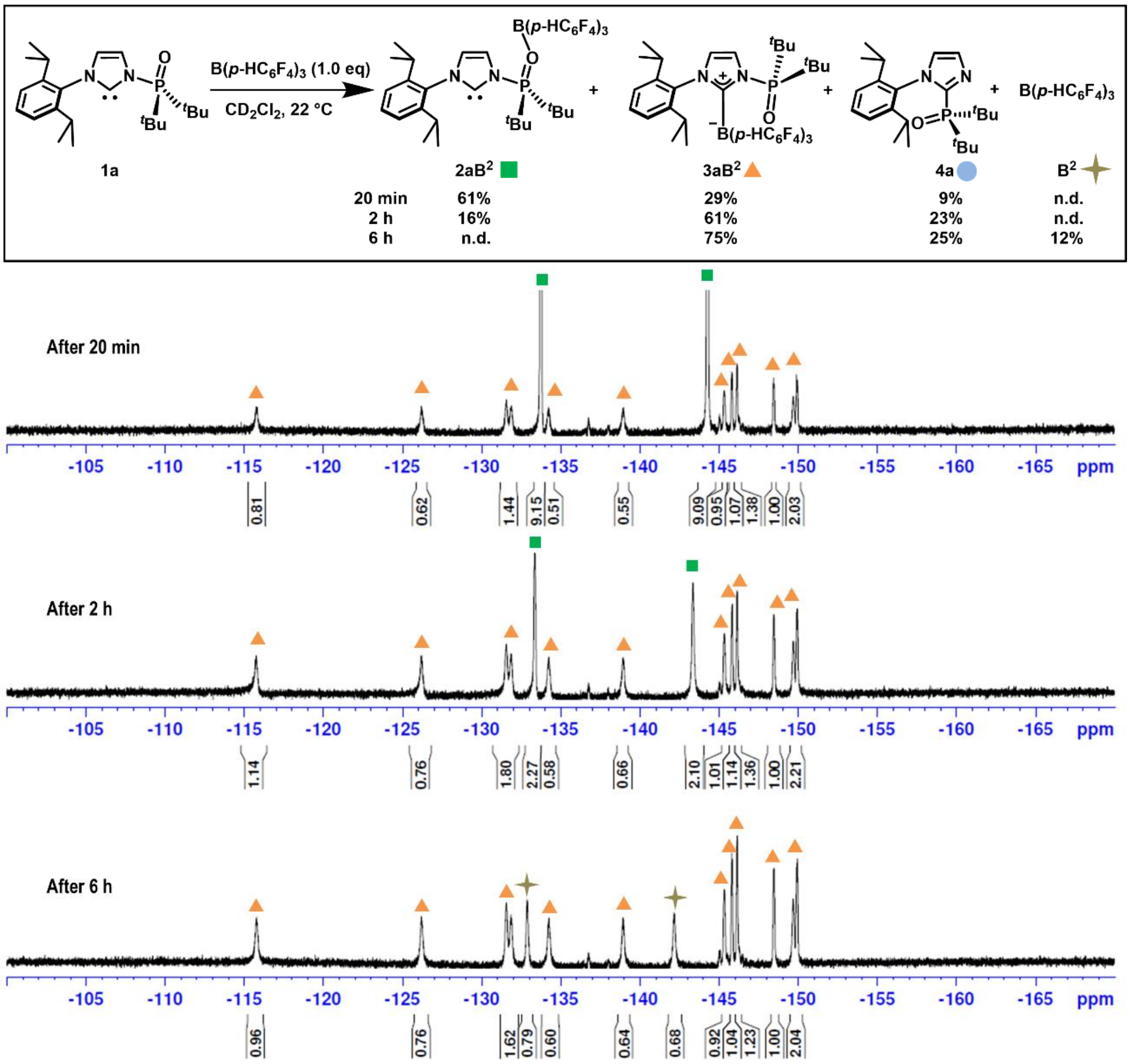


**Supplementary Figure 10.** Reaction between **1a** and $\mathbf{B^2}$ at 22 °C monitored by $^{19}$F NMR .

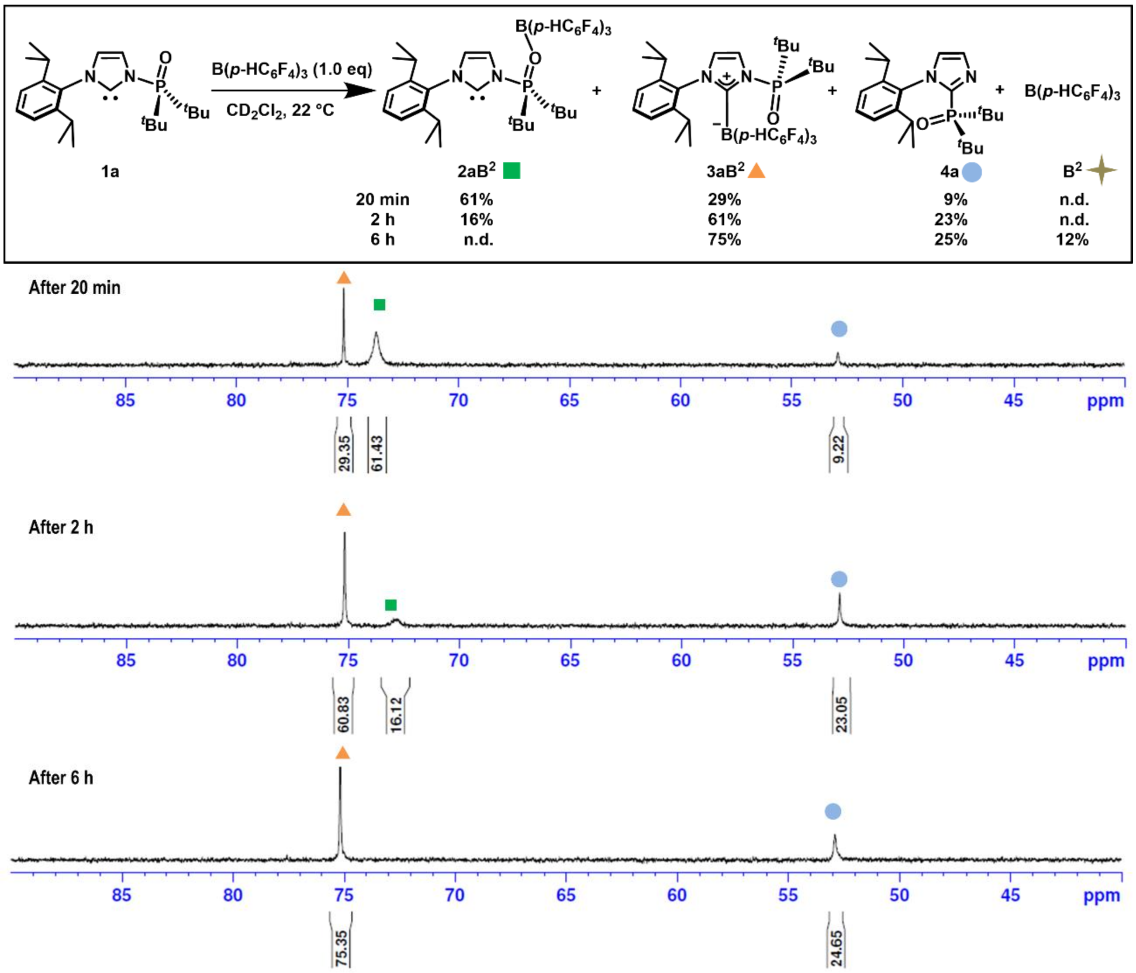


**Supplementary Figure 11.** Reaction between **1a** and $\mathbf{B^2}$ at 22 °C monitored by $^{31}$P NMR.

## 5-2. Thermolysis of 3aB$^2$

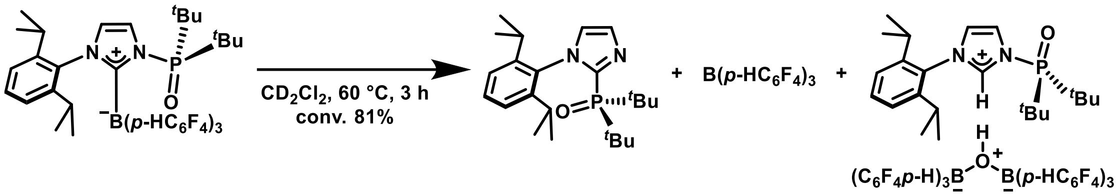


3aB$^2$ | 4a 77% | B$^2$ 73% | [1a–H][HO(B$^2$)$_2$] 4%

Compound **3aB$^2$** (8.4 mg, 0.01 mmol) and 1,2-dichloroethane (12.5 mg, 0.13 mmol; an internal standard) was dissolved in $CD_2Cl_2$ (0.5 mL). The resultant mixture was heated at 60 °C for 3 h and NMR analysis was carried out (Supplementary Figures 12–14), which showed the formation of **4a**, **B$^2$**, and **[1a–H][HO(B$^2$)$_2$]**.

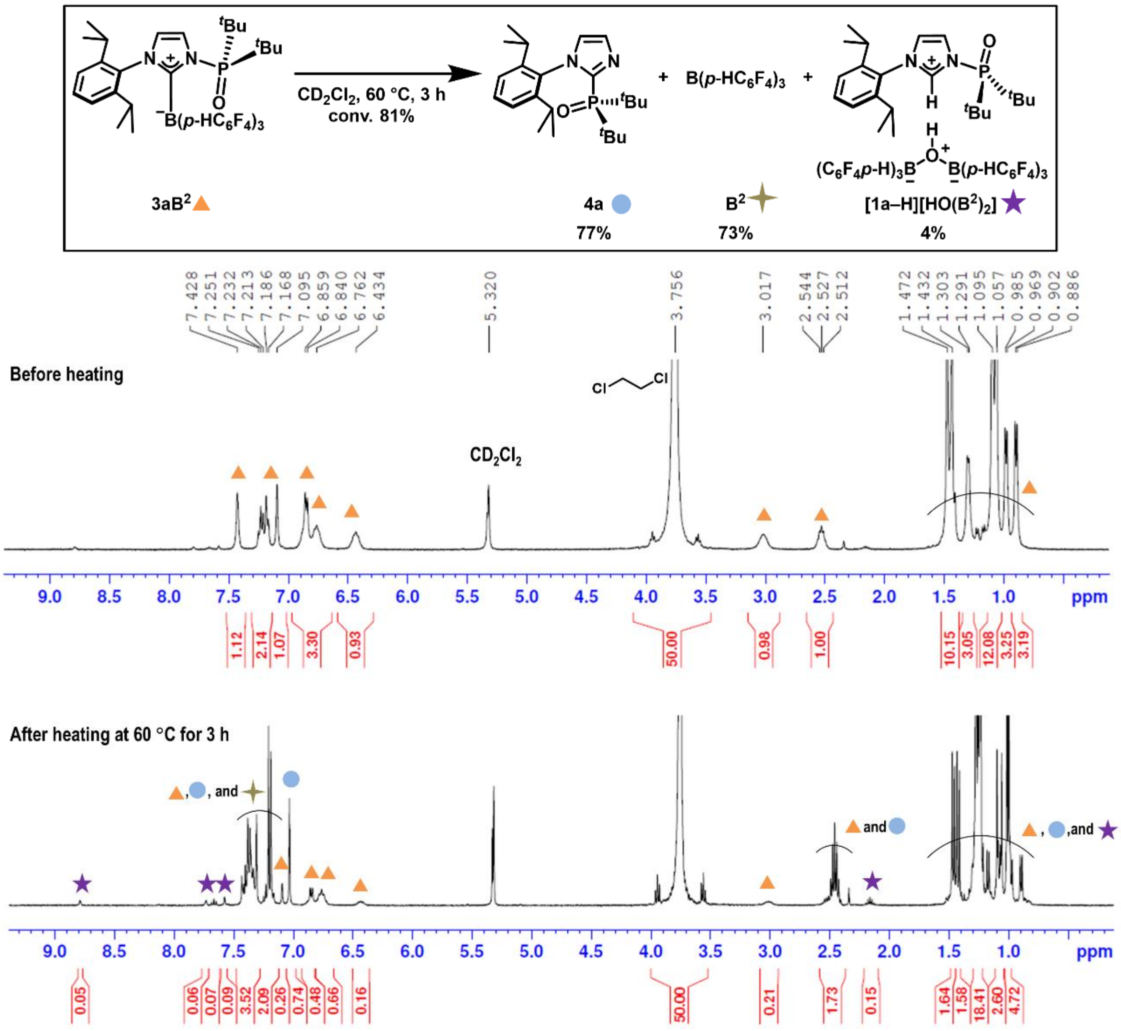


**Supplementary Figure 12.** Thermolysis of **3aB$^2$** at 60 °C monitored by $^1$H NMR.

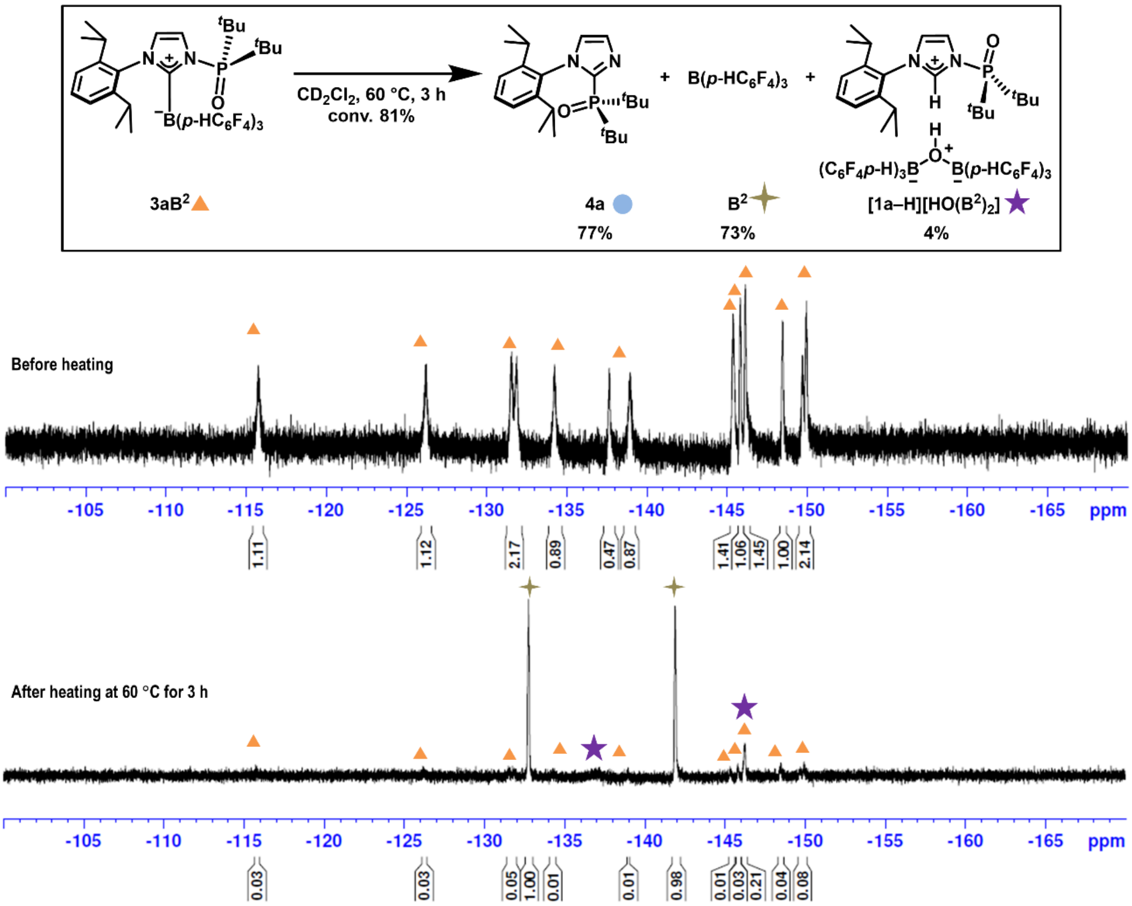


**Supplementary Figure 13.** Thermolysis of **3aB$^2$** at 60 °C monitored by $^{19}$F NMR.

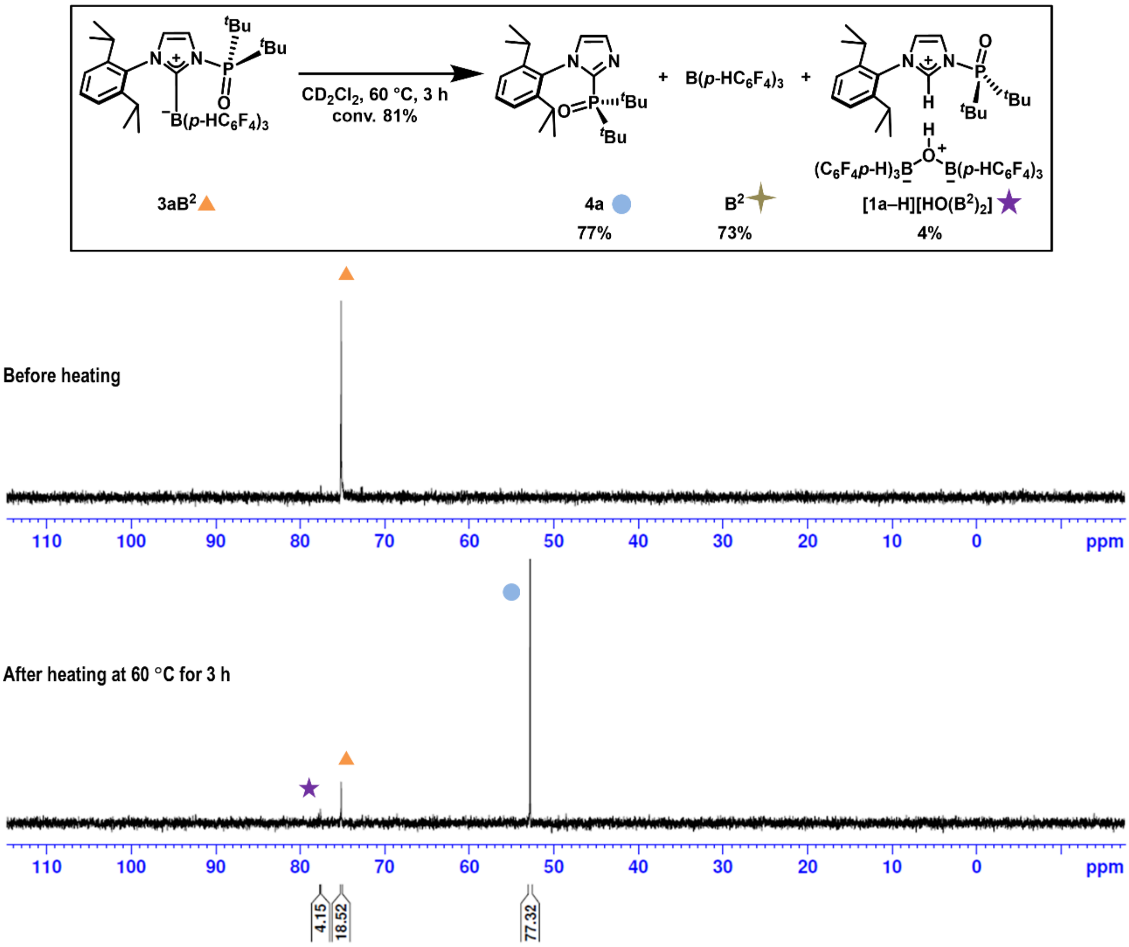


**Supplementary Figure 14.** Thermolysis of $\mathbf{3aB^2}$ at 60 °C monitored by $^{31}$P NMR.

## 5-3. Reaction between $3aB^2$ and $H_2$ at 22 °C

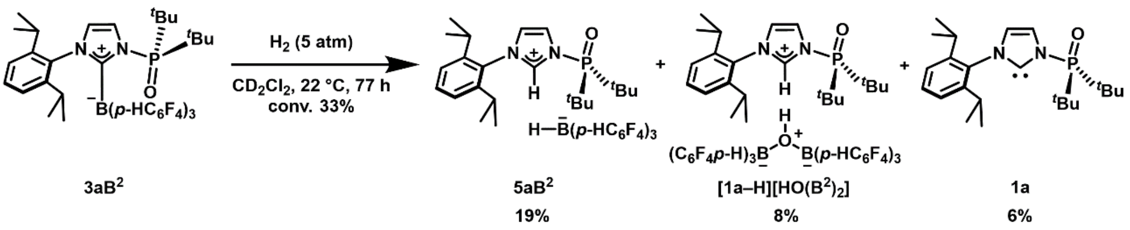


A solution of **$3aB^2$** (8.4 mg, 0.01 mmol) in $CD_2Cl_2$ (0.5 mL) was transferred into a pressure-tight NMR tube. Then, $H_2$ (5 atm) was pressurized, and the reaction was monitored at room temperature for 77 h by $^1H$, $^{19}F$, and $^{31}P$ NMR analyses (Supplementary Figures 15–17). Yield of products were estimated by the $^{19}F$ and $^{31}P$ NMR analyses.

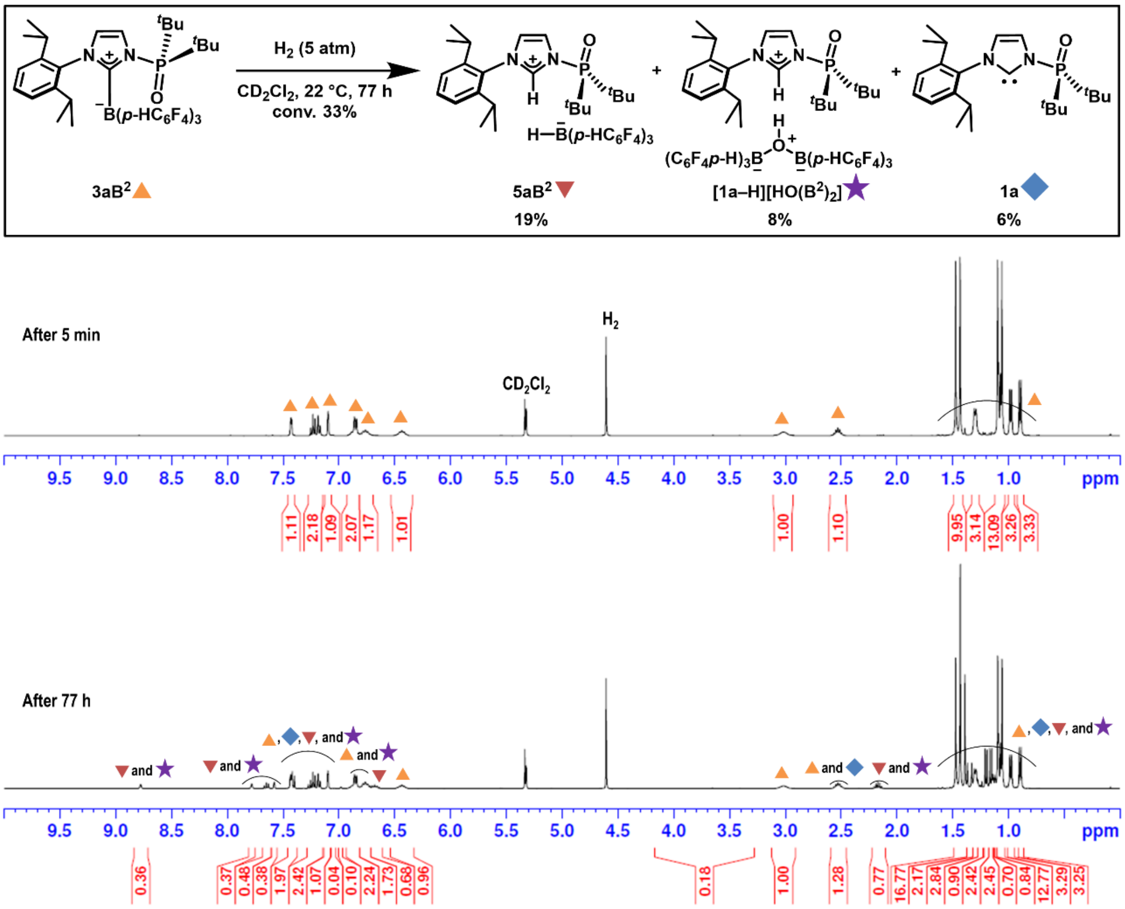


**Supplementary Figure 15.** Reaction between **$3aB^2$** and $H_2$ at 22 °C monitored by $^1H$ NMR.

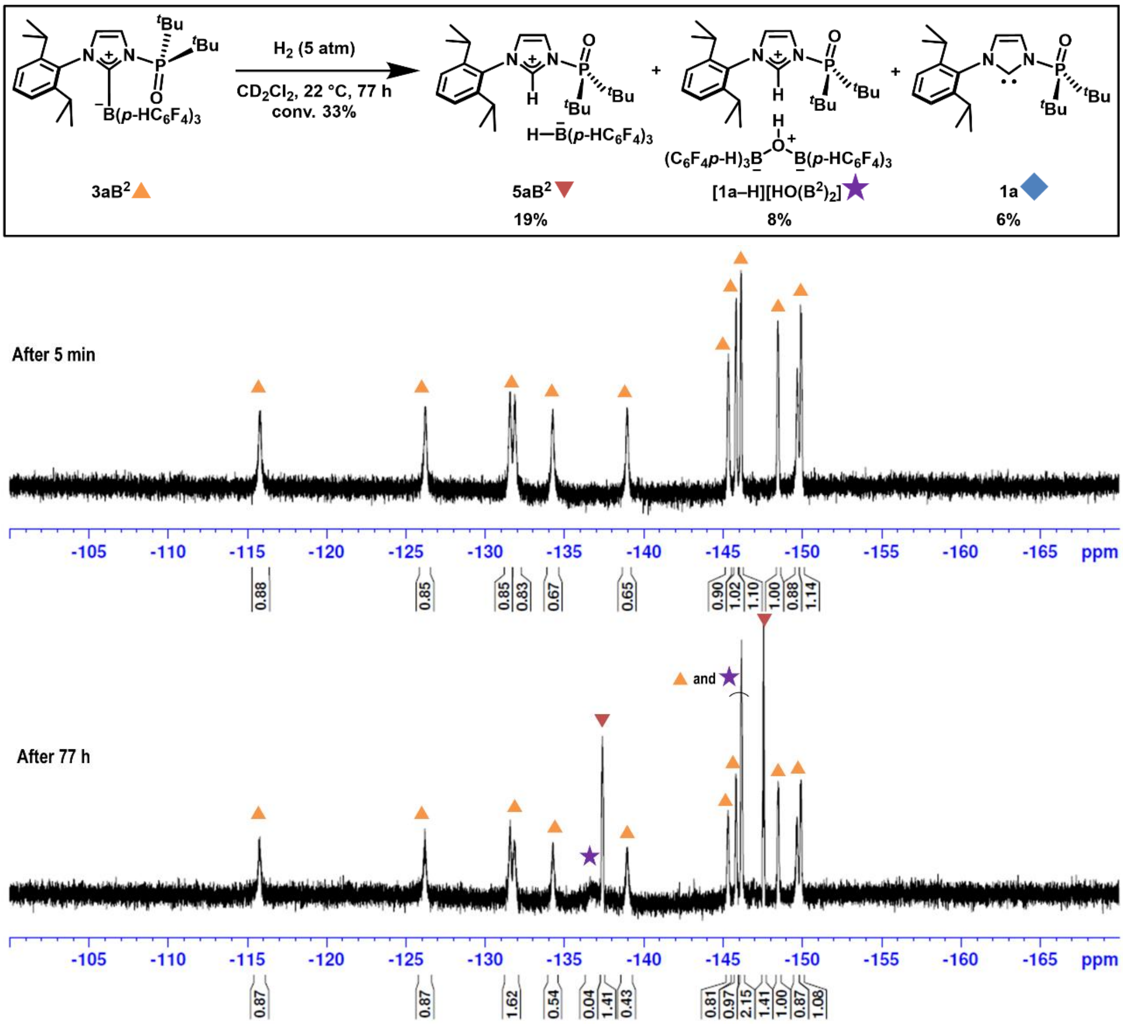


**Supplementary Figure 16.** Reaction between **$3aB^2$** and $H_2$ at 22 °C monitored by $^{19}F$ NMR.

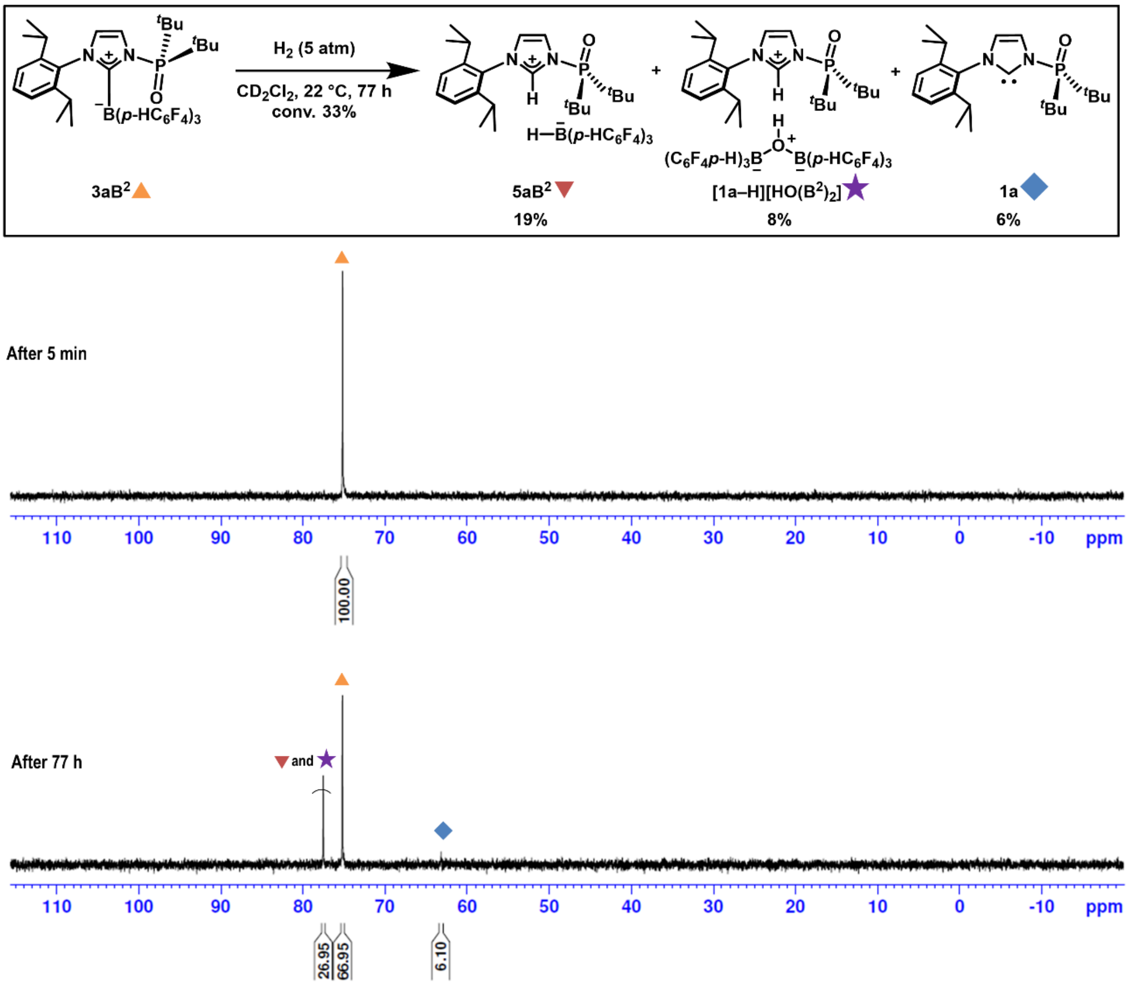


**Supplementary Figure 17.** Reaction between **3aB**$^{2}$ and $H_2$ at 22 °C monitored by $^{31}P$ NMR.

## 5-4. Reaction between $3aB^2$ and $H_2$ at 60 °C

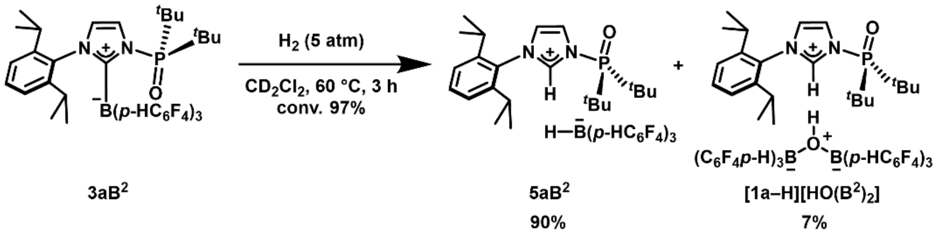


A solution of **$3aB^2$** (8.6 mg, 0.01 mmol) in $CD_2Cl_2$ (0.5 mL) was transferred into a pressure-tight NMR tube. The reaction mixture was heated at 60 °C for 3 h after pressurization of $H_2$ (5 atm), which was monitored by $^1H$, $^{19}F$, and $^{31}P$ NMR analyses (Supplementary Figures 18–20). Yield of products were estimated by the $^{19}F$ and $^{31}P$ NMR analyses.

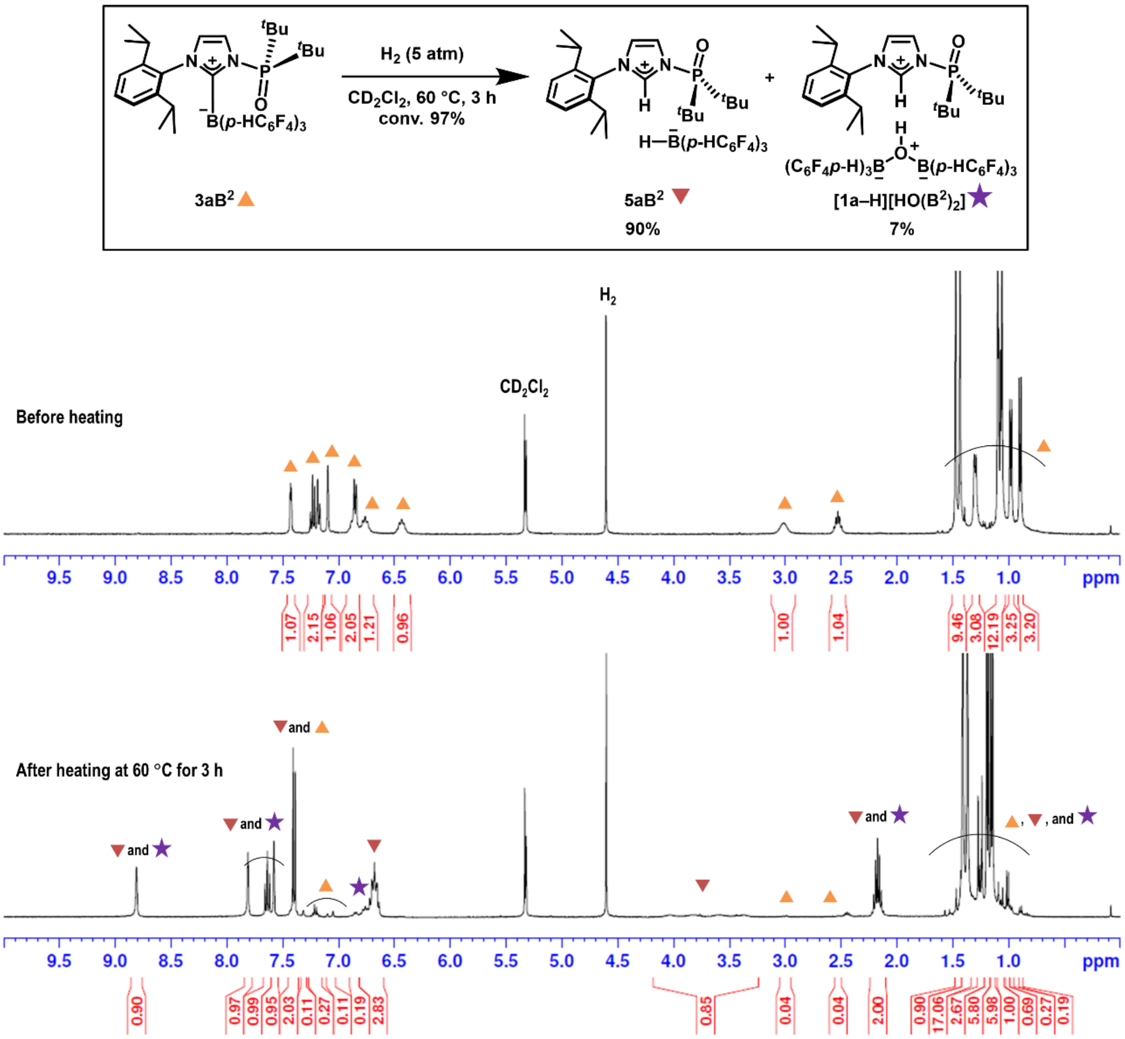


**Supplementary Figure 18.** Reaction between **$3aB^2$** and $H_2$ at 60 °C monitored by $^1H$ NMR.

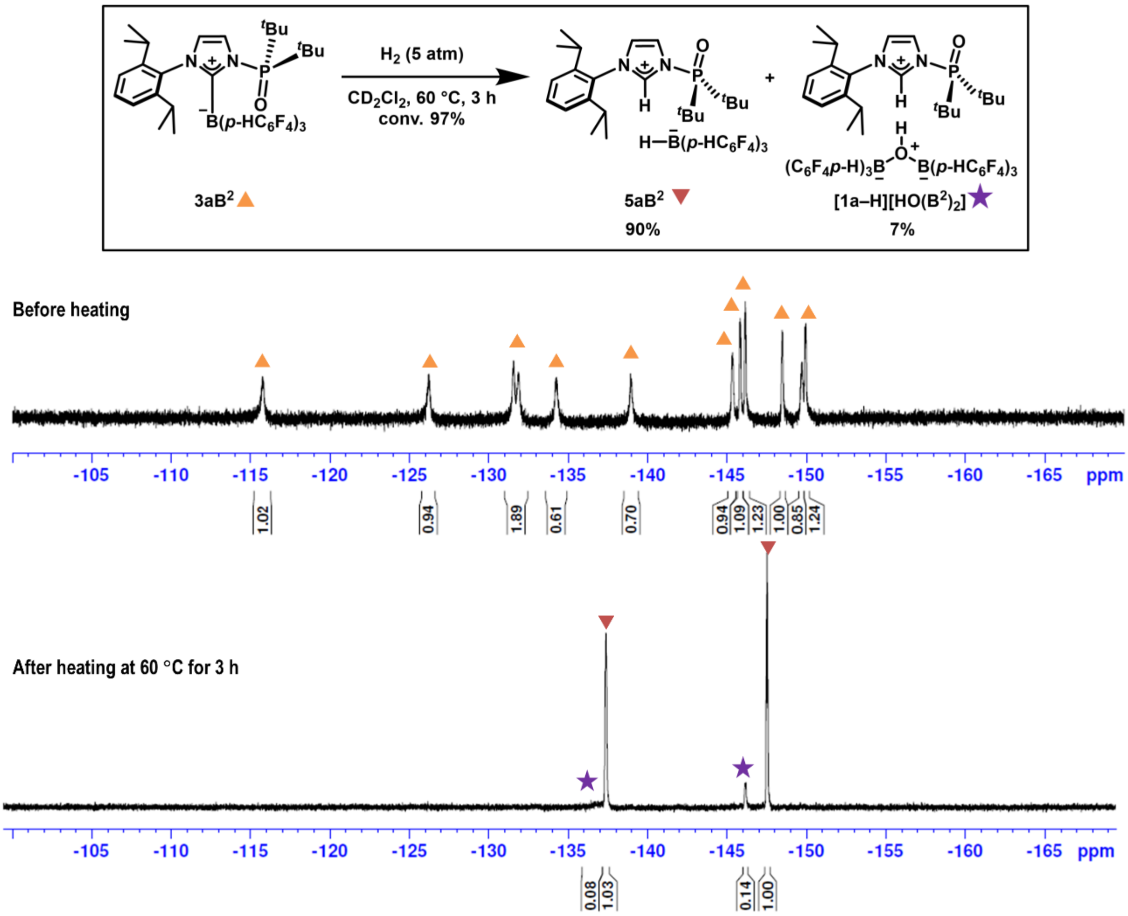


**Supplementary Figure 19.** Reaction between **3aB$^2$** and $H_2$ at 60 °C monitored by $^{19}F$ NMR.

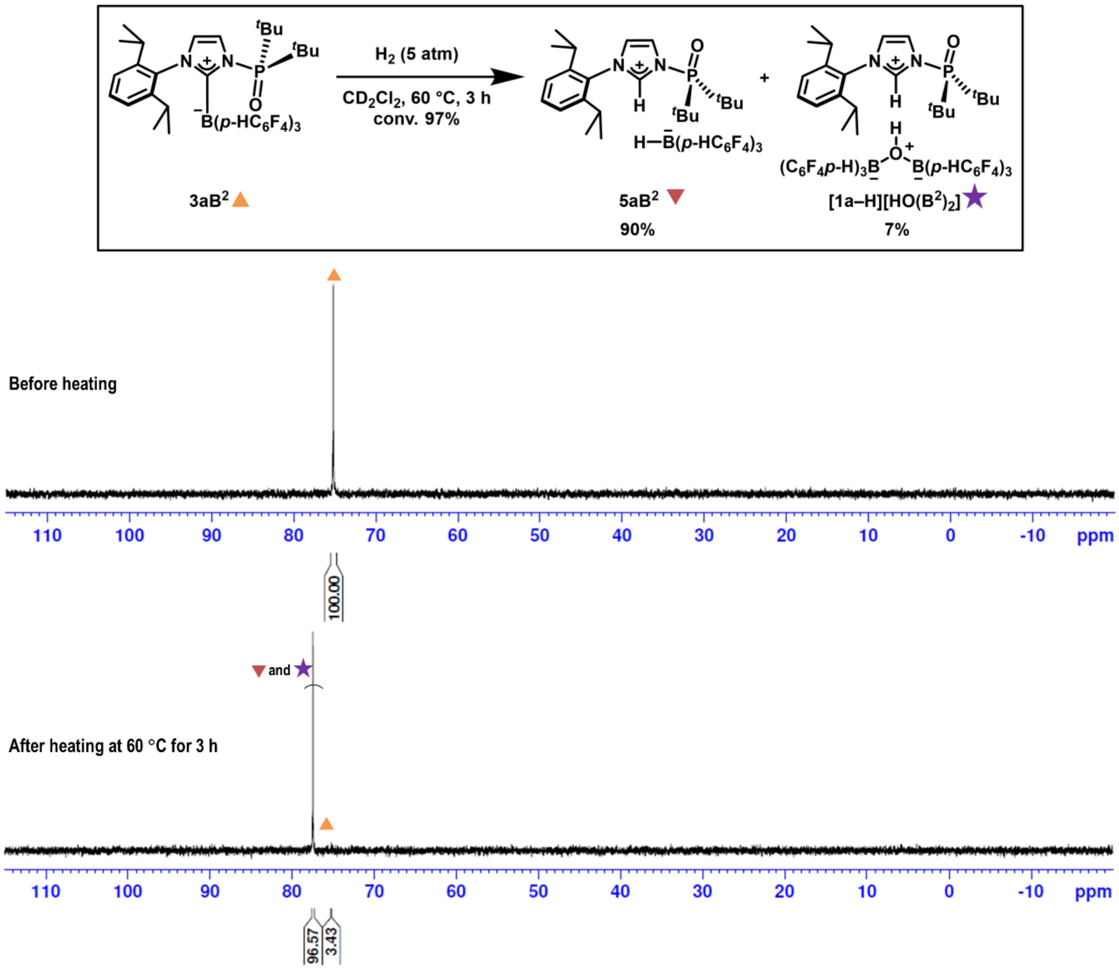


**Supplementary Figure 20.** Reaction between $\mathbf{3aB^2}$ and $H_2$ at 60 °C monitored by $^{31}$P NMR.

## 5-5. Reaction between 3aB[1] and $H_2$ at 60 °C

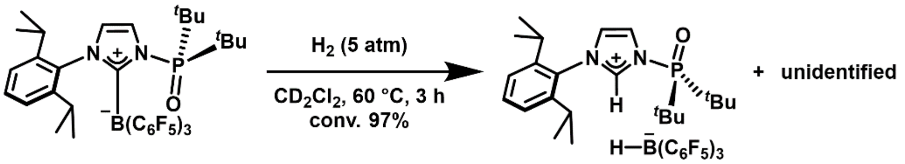


**3aB[1]** **5aB[1]** 89%

Compound **3aB[1]** (9.0 mg, 0.01 mmol) and mesitylene (1.0 mg, 0.01 mmol; an internal standard) was dissolved in $CD_2Cl_2$ (0.5 mL). After transferring this solution into a pressure-tight NMR tube, $H_2$ was pressurized at 5 atm. The reaction mixture was then heated at 60 °C for 3 h, monitored by the $^{1}H$, $^{19}F$, and $^{31}P$ NMR analyses (Supplementary Figures 21–23).

(We previously reported that the heterolytic cleavage of $H_2$ quantitatively proceeded by **3aB[1]** in the presence of excess amounts of $H_2$ (5 atm, ca. 30−50 equiv) in the autoclave reactor ($V$ = 25 mL, see ref. 1). In this work, we used a pressure-tight NMR tube ($V$ = 1.8 mL) that can contain less than 30 equivalents of $H_2$ after the pressurization at 5 atm.)

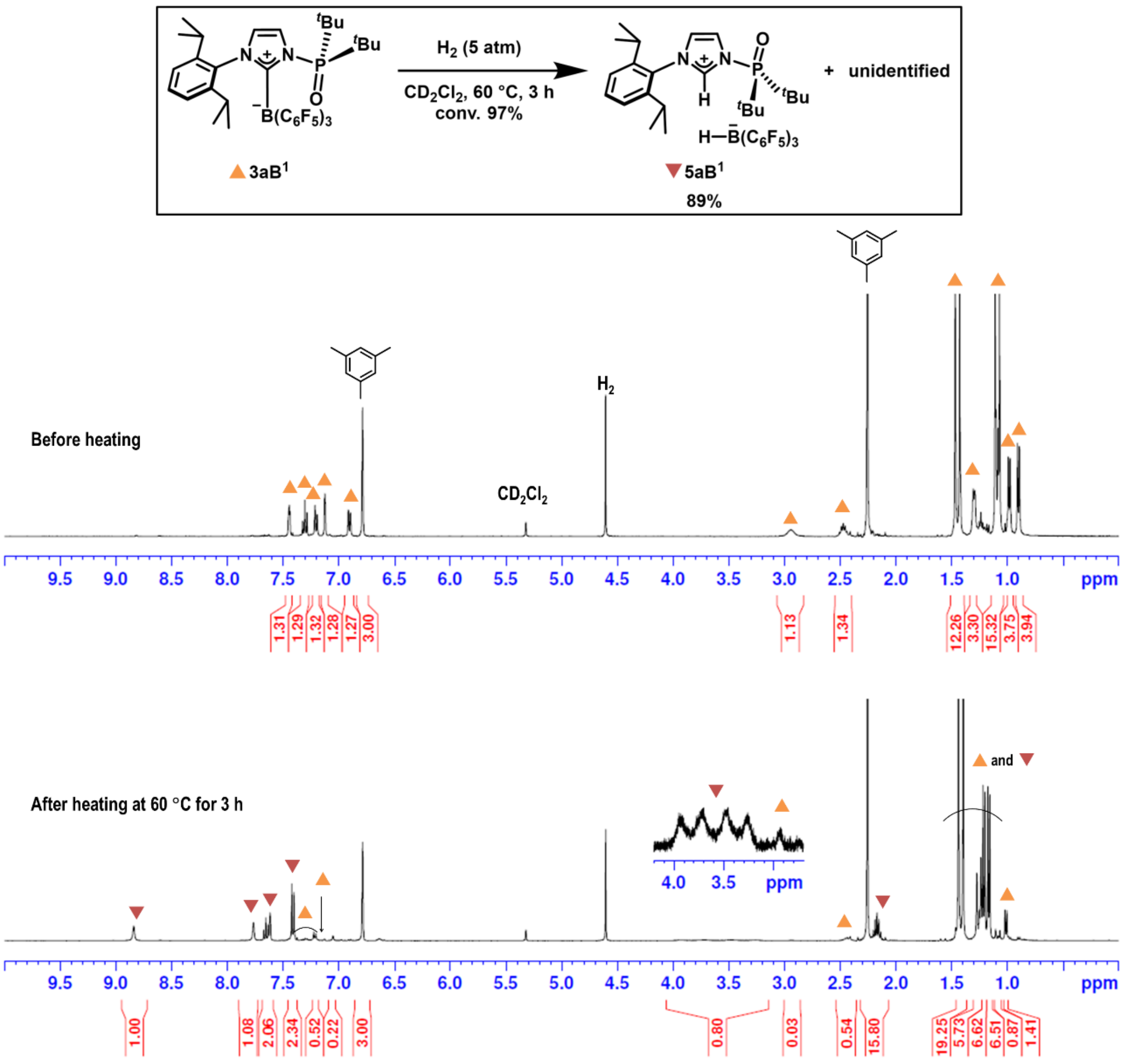


**Supplementary Figure 21.** Reaction between **3aB[1]** and $H_2$ at 60 °C monitored by $^{1}H$ NMR.

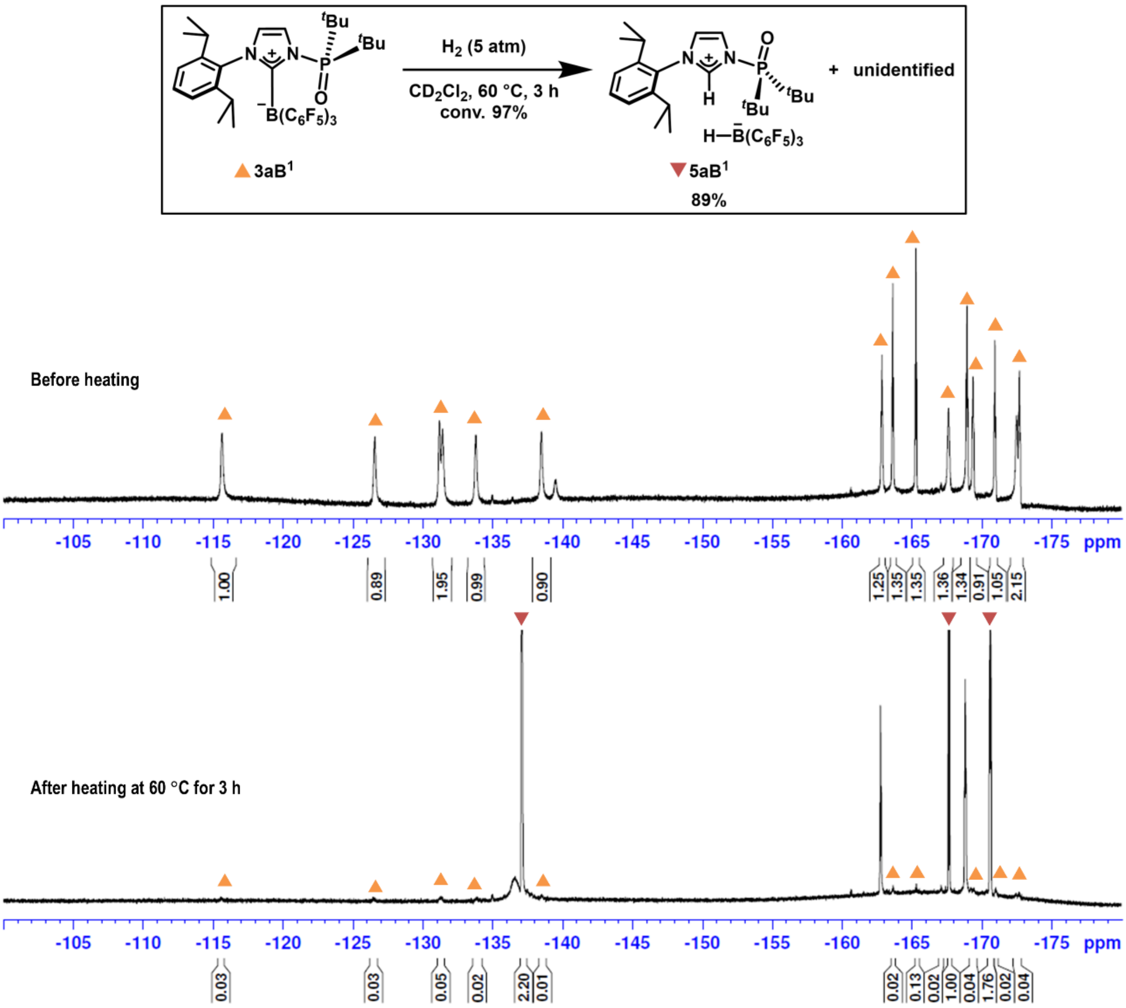


**Supplementary Figure 22.** Reaction between **3aB**[1] and $H_2$ at 60 °C monitored by $^{19}F$ NMR.

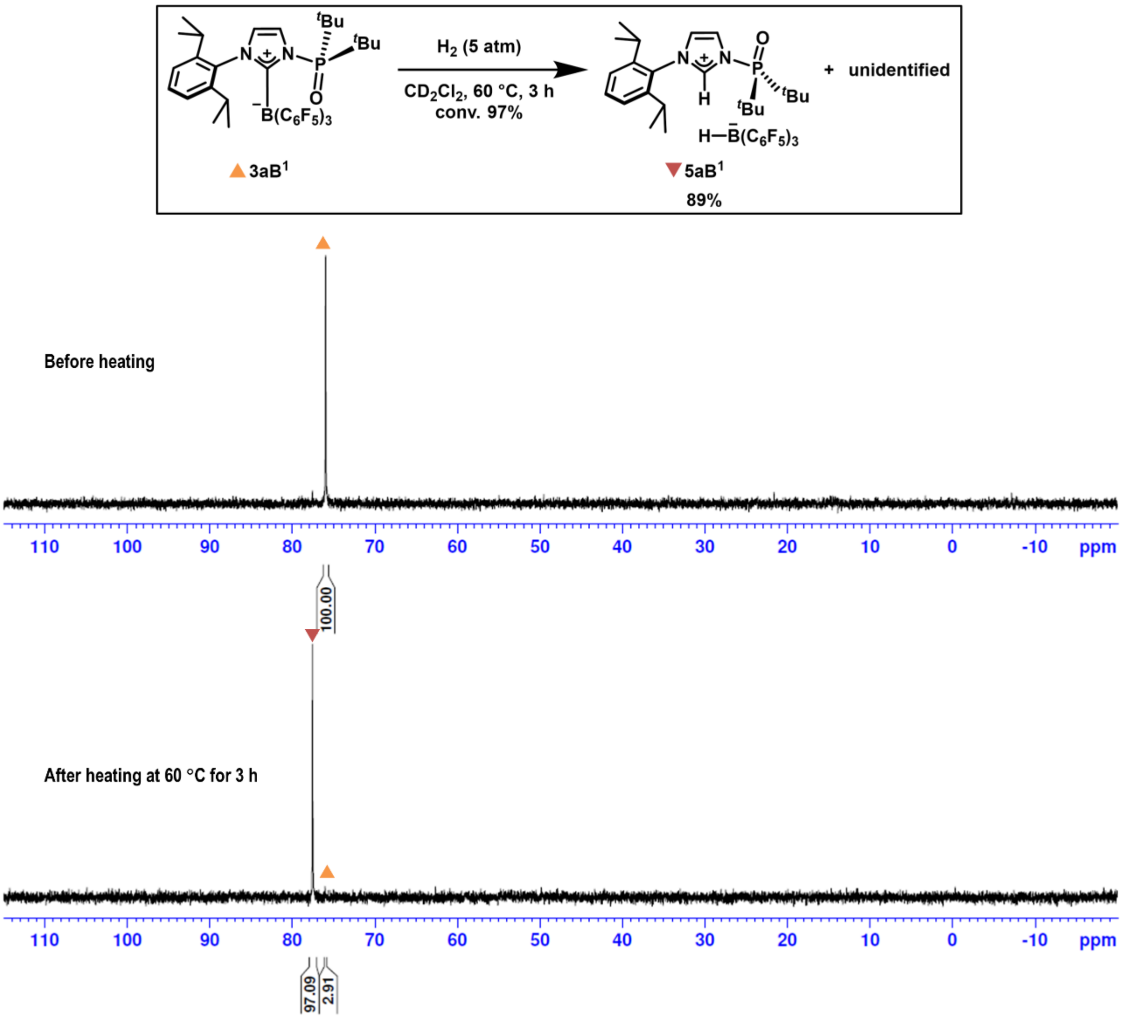


**Supplementary Figure 23.** Reaction between **3aB**[1] and $H_2$ at 60 °C monitored by $^{31}$P NMR.

## [6] Variable Temperature NMR Experiments

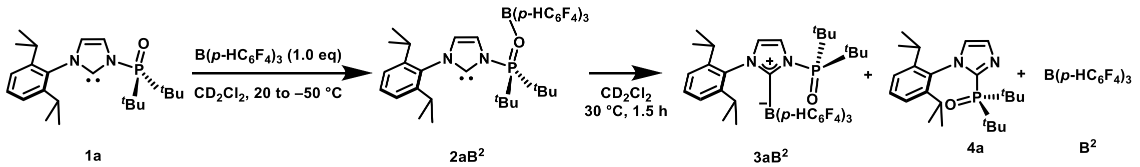


A J. Young NMR tube was charged with **1a** (7.6 mg, 0.02 mmol), **B**[2] (9.4 mg, 0.02 mmol), 1,2-dichloroethane (2.5 mg, 0.03 mmol; an internal standard), and $CD_2Cl_2$ (0.5 mL). The $^{1}$H, $^{19}$F, and $^{31}$P NMR analyses were once conducted at 20 °C, and then the temperature was changed to –50 °C. The NMR measurements were then conducted at every 20 °C. Then, the reaction was monitored for 1.5 h after the mixture was allowed to warm to 30 °C (Supplementary Figures 24–26).

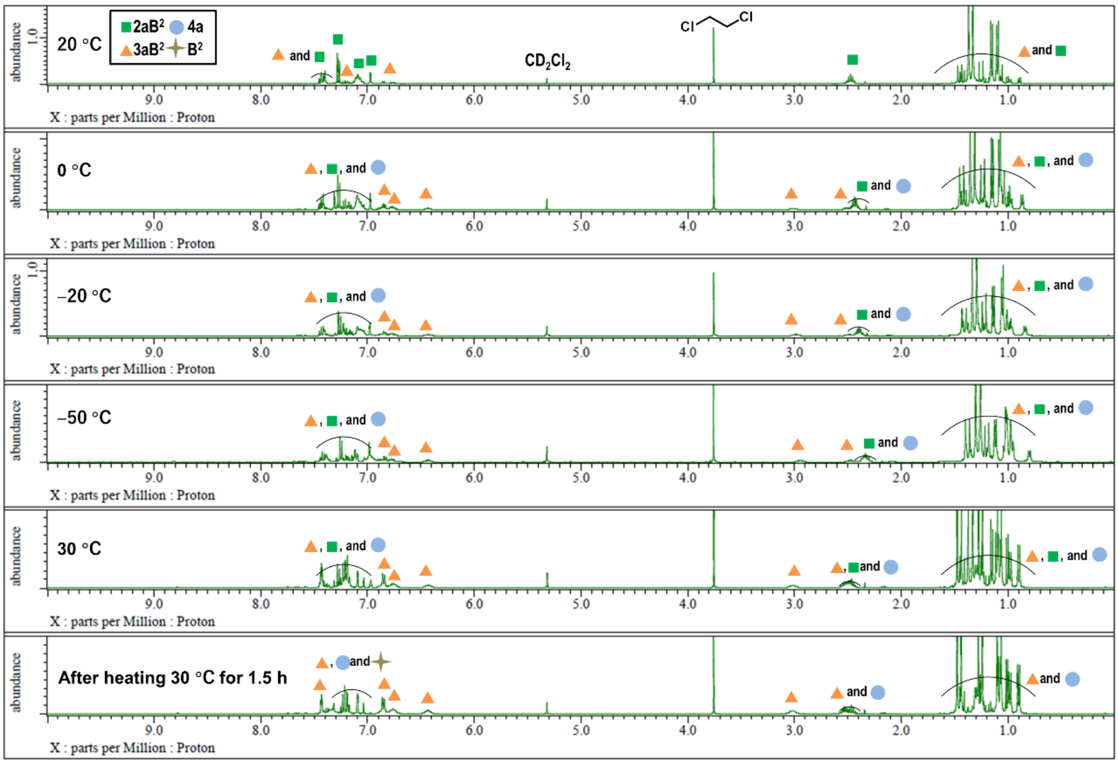


**Supplementary Figure 24.** $^{1}H$ NMR spectra obtained by the VT-NMR.

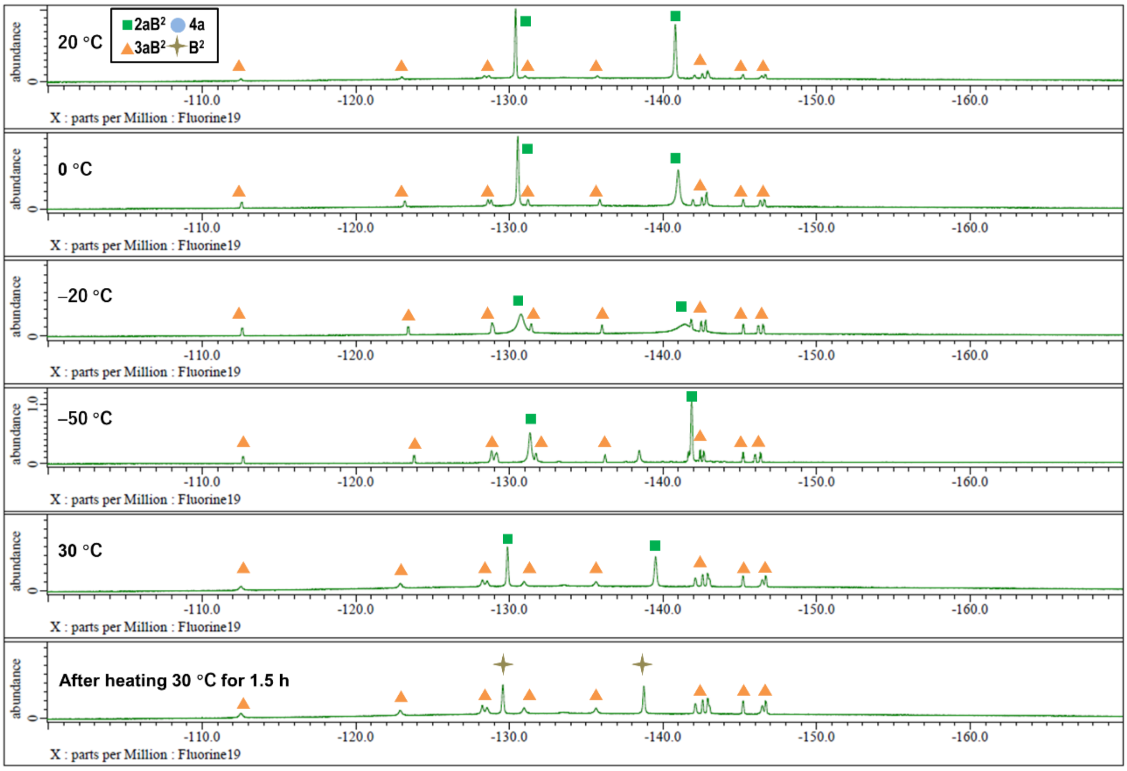


**Supplementary Figure 25.** $^{19}F$ NMR spectra obtained by the VT-NMR.

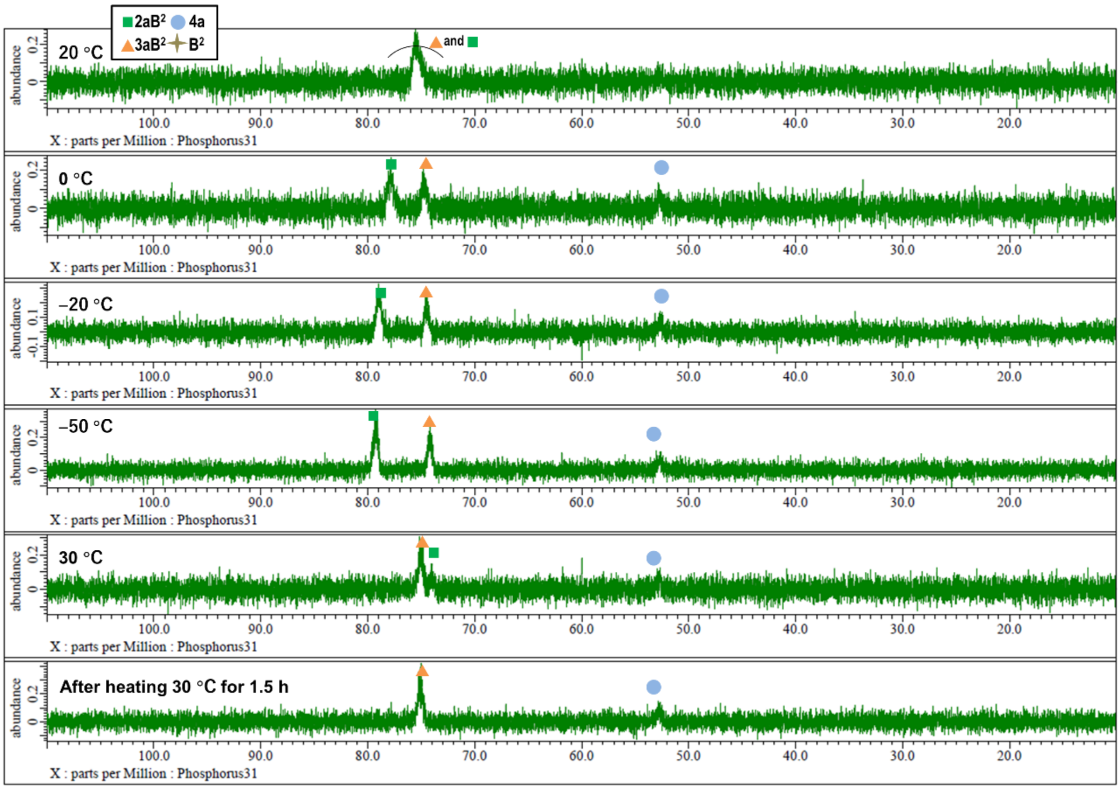


**Supplementary Figure 26.** $^{31}$P NMR spectra obtained by the VT-NMR.

## [7] Kinetics Studies
## 7-1. The Arrhenius plot

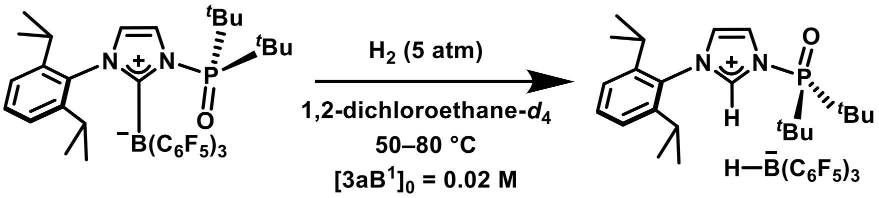


**3aB$^1$** **5aB$^1$**

A solution of **3aB$^1$** (9.0 mg, 0.01 mmol) and mesitylene (2.4 mg, 0.02 mmol; an internal standard) in 1,2-dichloroethane-$d_4$ (0.5 mL) was heated at 60 °C in the presence of $H_2$ that was pressurized at 5 atm into a pressure-tight NMR tube. The reaction was monitored by $^1$H NMR analysis. The rate constant of the production of **5aB$^1$** was evaluated by least-squares fitting of the time–conversion profiles to a first-order rate equation.

$$d[\mathbf{5aB^1}]/dt = k_{obs}[\mathbf{3aB^1}]$$
$$k_{obs} = 11.2(8)\ (10^{-5}\ s^{-1})$$

The aforementioned procedure was applied for the reaction between $H_2$ and **3aB$^1$** conducted at 50, 70, and 80 °C, respectively (Supplementary Figure 27). All experiments were repeated two times to give $k_{obs}$ ($10^{-5}$ $s^{-1}$) as an average of values obtained in each experiment (Supplementary Figure 28). These results were used for the analysis of reaction parameters estimated by the Arrhenius Equation (Eqn 1).

$$lnk_{obs} = -\left(\frac{E_a}{R}\right)\left(\frac{1}{T}\right) + lnA \quad (1)$$

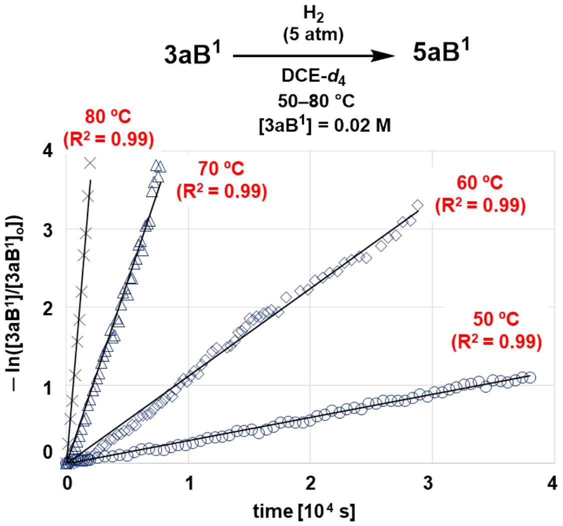


**Supplementary Figure 27.** The profiles of time ($10^4$ s) *vs* –ln([**3aB$^1$**]/[**3aB$^1$**]$_0$) at 50, 60, 70, and 80 °C.

| | Reaction rate constant, $k_{obs}$ ($10^{-5}$ s$^{-1}$) | | |
|---|---|---|---|
| $T$ (°C) | 1st run | 2nd run | average |
| 50 | 2.95(2) | 2.73(3) | 2.84(2) |
| 60 | 11.2(8) | 10.1(14) | 10.6(8) |
| 70 | 46.4(4) | 44.0(6) | 45.2(4) |
| 80 | 183(2) | 161(2) | 172(2) |

**Supplementary Figure 28.** The list of reaction rate constants, $k_{obs}$ ($10^{-5}$ s$^{-1}$).

## 7-2. Order in $H_2$

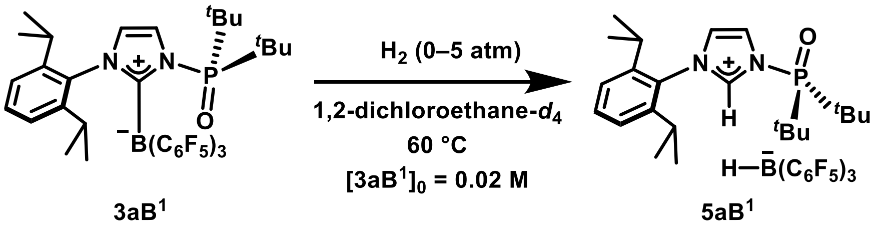


A solution of **3aB$^1$** (9.0 mg, 0.01 mmol) and mesitylene (internal standard) in 1,2-dichloroethane-$d_4$ (0.5 mL) was heated at 60 °C in the presence of $H_2$ that was pressurized at 0.5, 1, 2, and 5 atm, respectively, into a pressure-tight NMR tube (Supplementary Figure 29). The initial reaction rate constants ($k_{int}$ ($10^{-5}$ s$^{-1}$)) for the production of **5aB$^1$** were determined based on the results of the $^1$H NMR measurements. All experiments were repeated two times to give $k_{int}$ ($10^{-5}$ s$^{-1}$) as an average of values obtained in each experiment (Supplementary Figure 30).

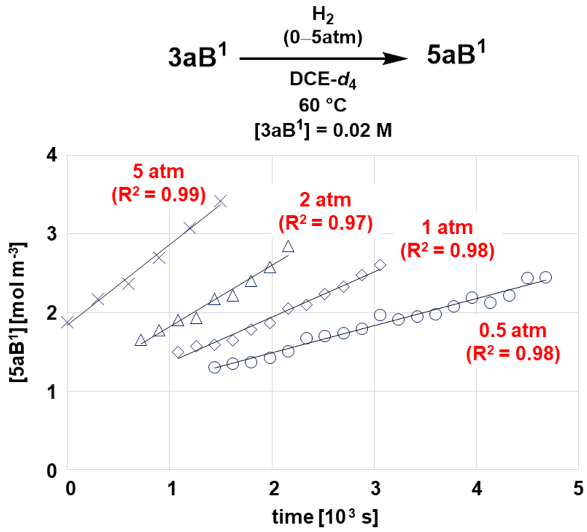


**Supplementary Figure 29.** The profiles of time ($10^3$ s) *vs* [**5aB**$^1$] (mol $m^{-3}$) at 0.5, 1, 2, and 5 atm.

| | Initial reaction rate constant, $k_{int}$ ($10^{-5}$ $s^{-1}$) | | |
|---|---|---|---|
| *P* (atm) | 1st run | 2nd run | average |
| 0 | 0 | 0 | 0 |
| 0.5 | 1.73(7) | 1.72(8) | 1.73(5) |
| 1 | 2.87(13) | 2.57(12) | 2.72(9) |
| 2 | 3.92(26) | 3.98(13) | 3.95(15) |
| 5 | 5.12(25) | 5.13(36) | 5.12(22) |

**Supplementary Figure 30.** The list of initial reaction rate constants, $k_{int}$ ($10^{-5}$ $s^{-1}$).

# [8] Theoretical Studies

## 8-1. Computational details

Geometry optimizations and energy calculations were performed by using the density functional theory (DFT) at the *ω*B97X-D[4]/6-31G(d)[5–6] level. For the H atoms in $H_2$ molecule, a p-type polarization function was augmented.[6] For the thermal correction, temperature and pressure were set to 298.15 K and 1.0 atm, respectively. At the optimized structures, we performed single-point calculations with the 6-311G(d,p) basis sets with solvation effect. A polarizable continuum model[7] was adopted, and the parameters for 1,2-dichloroethane (DCE) were used. The Gaussian 09 package was used for the DFT calculations.[8]

The analyses on the quantum theory of the atoms in molecules were carried out using AIMAll program (Version 19.10.12),[9] in which the wave functional wiles were prepared based on the optimized electron density at the *ω*B97X-D/6-31G(d) level of theory.

## 8-2. Details on proposed mechanism

The relative Gibbs energies shown in Supplementary Figures 31–33 are shown in kcal mol$^{-1}$ with respect to that of **[1a + B$^1$]**.

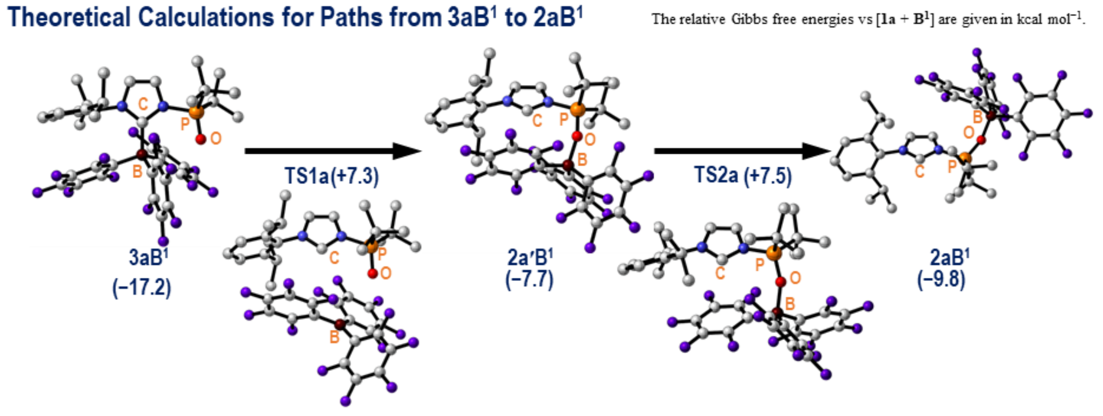


Selected bond lengths/(interatomic distances) (Å) and angles (°)

| | 3aB$^1$ | TS1a | 2a′B$^1$ | TS2a | 2aB$^1$ |
|---|---|---|---|---|---|
| C–B | 1.71 | (4.24) | — | — | — |
| O–B | (3.28) | (3.34) | 1.59 | 1.62 | 1.58 |
| P-O-B | 92.8 | 141.8 | 152.7 | 159.8 | 150.2 |
| C-N-P-O | 3.92 | 43.3 | 35.5 | 60.0 | 156.0 |

**Supplementary Figure 31.** Theoretical calculations for paths from **3aB$^1$** to **2aB$^1$**.

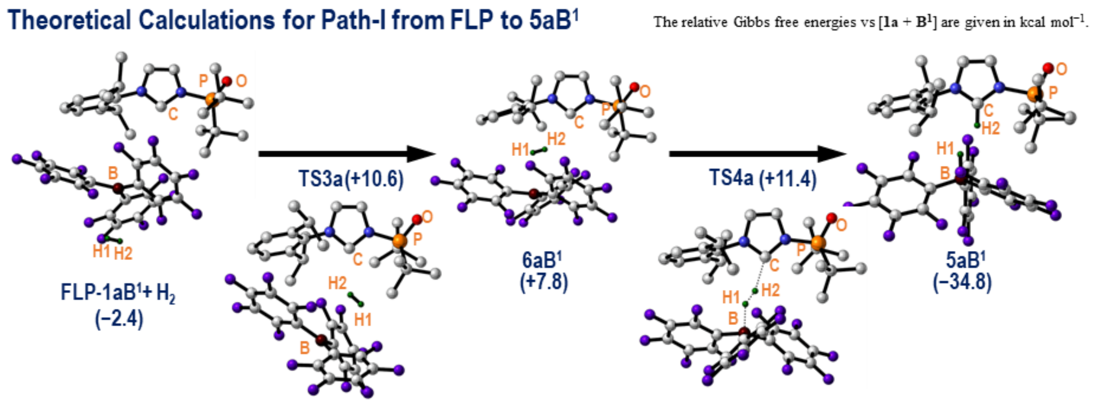


Selected bond lengths/(interatomic distances) (Å) and angles (°)

| | FLP-1aB$^1$ | TS3a | 6aB$^1$ | TS4a | 5aB$^1$ |
|---|---|---|---|---|---|
| H1–H2 | 0.74 | 0.74 | 0.75 | 0.84 | (1.69) |
| C–H2 | — | (3.40) | (2.58) | (1.83) | 1.08 |
| B–H1 | — | (2.80) | (2.36) | (1.49) | 1.22 |
| C-N-P-O | 170.5 | 158.2 | 169.8 | 170.0 | 172.0 |

**Supplementary Figure 32.** Theoretical calculations for path I, affording **5aB$^1$** from **FLP-1aB$^1$**.

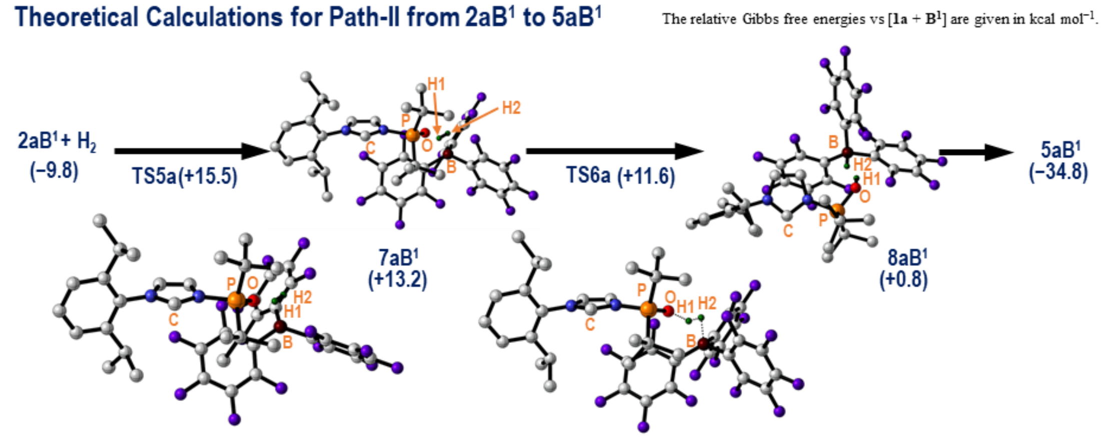


Selected bond lengths/(interatomic distances) (Å) and angles (°)

| | TS5a | 7aB¹ | TS6a | 8aB¹ |
|---|---|---|---|---|
| H1–H2 | 0.76 | 0.84 | 0.85 | 1.44 |
| O–H1 | 1.93 | 1.50 | 1.45 | 0.99 |
| B–H2 | 1.76 | 1.34 | 1.33 | 1.23 |
| C-N-P-O | 159.8 | 163.7 | 163.6 | 171.6 |

**Supplementary Figure 33.** Theoretical calculations for path II, affording **5aB¹** from **2aB¹**.

**Discussions for TS1.** The potential energy surface around **TS1a** was found to be relatively flat (Supplementary Figure 34). As ordinary IRC calculations were difficult to apply, we thus performed a relaxed potential energy scan calculation that approximately shows the connection from **2a’B¹** to **3aB¹** via **TS1a**.

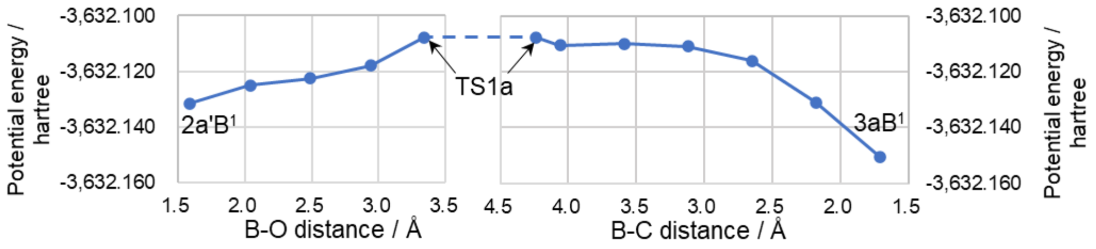


**Supplementary Figure 34.** Minimum energy potential pathway from **2a’B¹** to **3aB¹** via **TS1a**, obtained from a relaxed potential energy scan calculation. For the reaction coordinate from **2a’B¹** to **TS1a**, the B-O(P=O) distance was used. For that from **3aB¹** to **TS1a**, the B-C(carbene) distance was used.

**Discussions for TS3a.** Relative potential energies are shown in Supplementary Figure 35a for the structures along a minimum energy pathway. To the left of **TS3a,** the result of an IRC calculation is shown. To the right of **TS3a**, the relative potential energy is plotted for structures along the steepest decent pathway that was obtained from a structural optimization with very small increments. This optimization terminated at a metastable minimum which is energetically 0.4 kcal/mol higher than the **6aB[1]** state. This metastable point is structurally close to that of the **6aB[1]** state (Supplementary Figures 35b and 35c). Therefore, under thermal fluctuations in the experimental conditions, the system can be expected to reach the **6aB[1]** state.

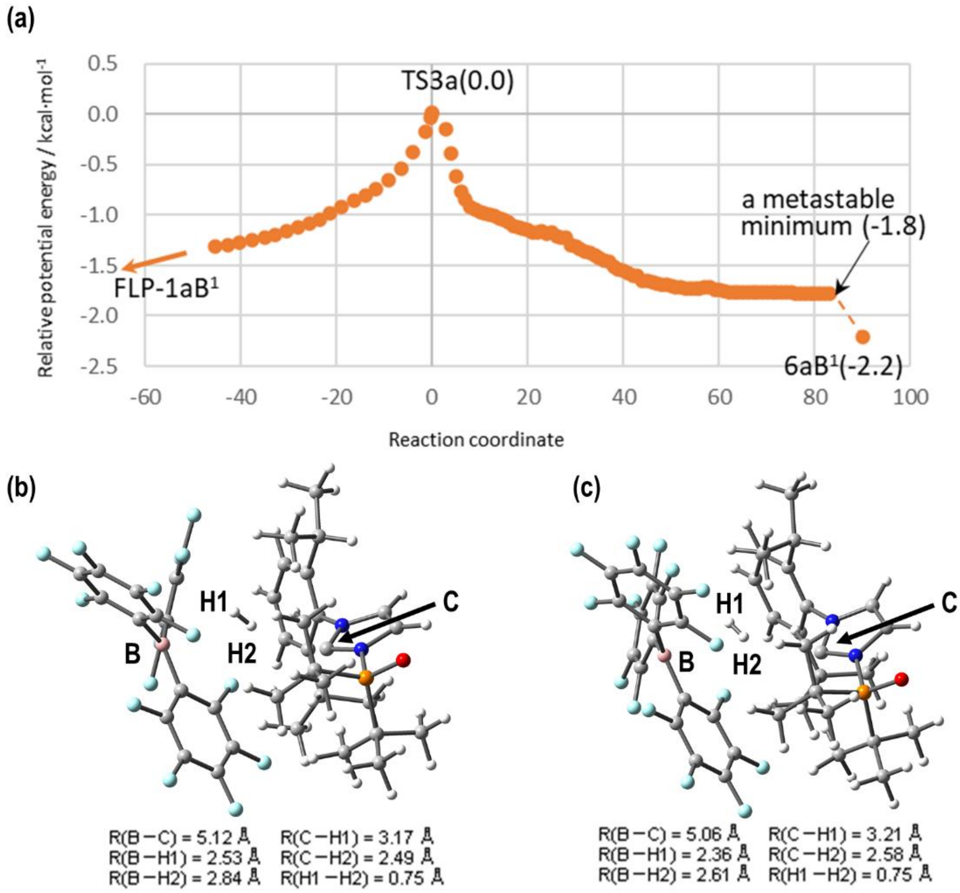


**Supplementary Figure 35**. (a) Relative potential energies of structures along a minimum energy pathway via **TS3a**. For the reaction coordinates from 0 (**TS3a**) in negative direction, an IRC is given. Those from 0 in positive direction represent potential energies of structures along the steepest descent direction obtained from a structural optimization with very small increments. For the **6aB[1]** state, a reaction coordinate of 90 is given only for representation purposes. The numbers in parentheses are potential energy values relative to that of **TS3a**. Structures of (b) the metastable minimum state and (c) the **6aB[1]** state with selected structural parameters.

**Discussions for TS5a**. Relative potential energies are shown in Supplementary Figure 36 for the structures along a minimum energy pathway. To the right of **TS5a**, the result of the IRC calculation is given. To the left of **TS5a**, the relative potential energy is plotted for structures along the steepest decent pathway obtained from a structural optimization with very small increments. This optimization terminated at the **7aB$^1$** state, which shows the connectivity between the two stationary points.

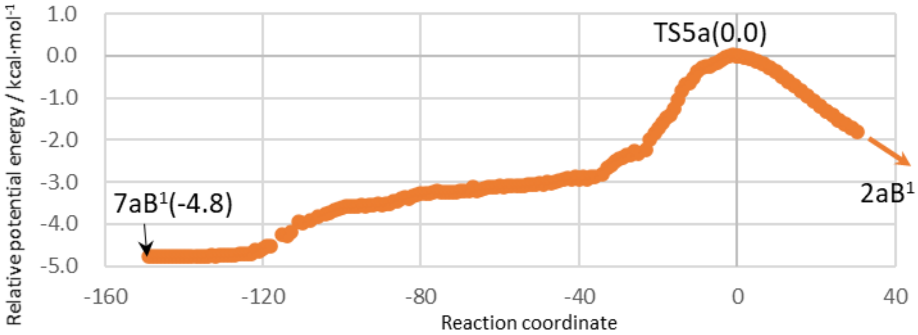


**Supplementary Figure 36**. Relative potential energies of structures along a minimum energy pathway via **TS5a**. For reaction coordinates from 0 (**TS5a**) in positive direction, the result of the IRC calculation is given. Those from 0 in negative direction are potential energies of structures along the steepest descent direction given by structural optimization with very small increments. The numbers in parentheses represent potential energy values relative to that of **TS5a**.

**Discussions for TS6a.** Given the results on the structural optimization, the potential energy of **TS6a** (-3633.288355 hartree) is found to be very close to that of **7aB$^1$** (-3633.288363 hartree). In general, Gibbs energy correction (ΔGibbs) is positive, and ΔGibbs to a transition state is smaller than that of an equilibrium state. This causes the reversed energy level between **TS6a** and **7aB$^1$** found in Figure 5b. The energy difference after the Gibbs correction and implementation of solvent effect was 1.5 kcal/mol, of which 1.4 kcal/mol arises from ΔGibbs.

## 8-3. The AIM analysis of TS1a

Detailed values on each BCPs are provided in Supplementary Data 3.

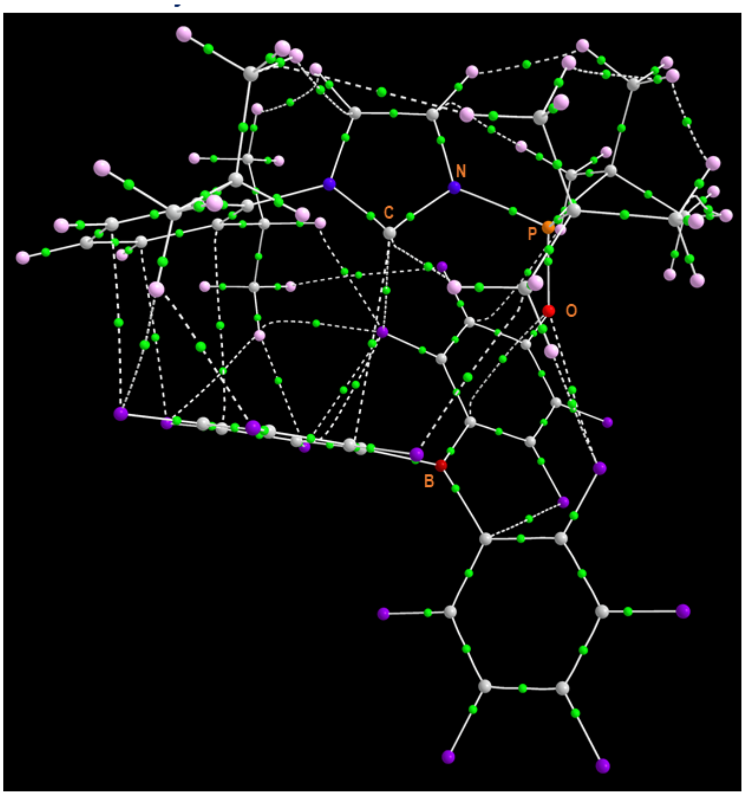


AIM calculations were carried out by AIMAll program based on the SCF densities obtained at the ωB97X-D/6-31G(d) level.
The AIM bond paths and bond critical points ($e \times r_{Bohr}^{-3}$) are shown in gray lines and green dots, respectively.

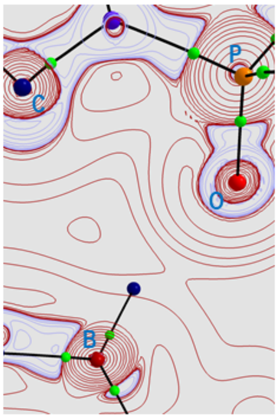


The selected AIM bond paths and bond critical points ($e \times r_{Bohr}^{-3}$) with overlaid contour plots of $\nabla^2\rho$ through the plane defined by the C, B, and O atoms, which clearly shows that none of the NCI are included between C/O and B atoms.

**Supplementary Figure 37.** Results of the AIM analysis of **TS1a**.

## 8-4. Optimized structure (cartesian coordinate) and potential energy of the stationary points during the reaction pathways

"E1"and"G1"denote potential energy and Gibbs free energy with 6-31G(d,p) for $H_2$ and 6-31G(d) for others in gas phase. E2 denotes potential energy with the 6-311G(d,p) basis sets with the solvation effect. Units for the coordinates and energy are in Å and atomic units, respectively.

E1 = -1.175055, G1 = -1.176368, E2 = -1.176203

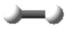

| | | | |
|---|---|---|---|
| H | 0. | 0. | 0.37123 |
| H | 0. | 0. | -0.37123 |

**Supplementary Table 1.** Computed Cartesian coordinates (x, y, z) for the optimized structure of $H_2$.

E1 = -2207.595797, G1 = -2207.499918, E2 = -2208.214841

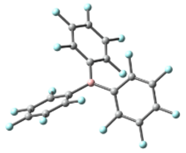

| | | | |
|---|---|---|---|
| F | -1.91767 | 1.59859 | 1.48644 |
| F | -2.02914 | 4.2705 | 1.4808 |
| F | -0.26592 | 5.7054 | -0.00249 |
| F | 1.62469 | 4.4382 | -1.48221 |
| F | 1.76301 | 1.76792 | -1.4825 |
| F | 2.34944 | 0.8699 | 1.47462 |
| F | 4.71898 | -0.3682 | 1.4691 |
| F | 5.07658 | -2.6215 | -0.00114 |
| F | 3.03018 | -3.63526 | -1.46894 |
| F | 0.64823 | -2.42067 | -1.47036 |
| F | -0.42469 | -2.47067 | 1.47513 |
| F | -2.684 | -3.90011 | 1.46819 |
| F | -4.81144 | -3.08098 | -0.0051 |
| F | -4.66151 | -0.80216 | -1.47266 |
| F | -2.4168 | 0.65015 | -1.47248 |
| C | -0.07212 | 1.56365 | 0.00166 |
| C | 0.82133 | 2.34665 | -0.73416 |
| C | 0.76542 | 3.73211 | -0.75497 |
| C | -0.20408 | 4.38285 | -0.00129 |
| C | -1.10845 | 3.64589 | 0.75409 |
| C | -1.03477 | 2.26122 | 0.73613 |
| C | 1.39187 | -0.72019 | 0.00222 |
| C | 1.6218 | -1.88974 | -0.72758 |
| C | 2.84981 | -2.53375 | -0.74789 |
| C | 3.9 | -2.01442 | -0.00027 |
| C | 3.71581 | -0.85821 | 0.74842 |
| C | 2.47944 | -0.23039 | 0.73048 |
| C | -1.31915 | -0.84533 | 0.00134 |
| C | -2.4459 | -0.45809 | -0.72925 |
| C | -3.61867 | -1.19805 | -0.75041 |
| C | -3.6966 | -2.36687 | -0.00271 |
| C | -2.60453 | -2.78646 | 0.74752 |
| C | -1.4414 | -2.03157 | 0.73021 |
| B | 0.00036 | -0.00119 | 0.00226 |

**Supplementary Table 2.** Computed Cartesian coordinates (x, y, z) for the optimized structure of **B[1]**.

E1 = -1424.491118, G1 = -1423.978013, G2 = -1424.802438

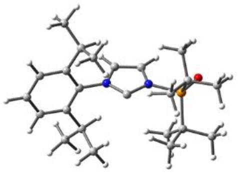

| | | | | | | | |
|---|---|---|---|---|---|---|---|
| P | 2.71269 | -0.04806 | 0.49015 | H | -1.69092 | -2.39728 | 0.91396 |
| O | 3.44966 | -0.06927 | 1.79121 | H | -1.05766 | -2.20672 | -1.4747 |
| N | -1.106 | -0.05469 | 0.6788 | H | -2.50607 | -3.12024 | -1.94208 |
| N | 1.00826 | -0.12211 | 0.83154 | H | -1.23141 | -3.91072 | -0.99025 |
| C | 0.00392 | -0.06093 | -0.10466 | H | -4.15879 | -3.89652 | -0.10091 |
| C | -0.81453 | -0.10355 | 2.04246 | H | -3.9712 | -3.2763 | 1.55019 |
| C | 0.52892 | -0.14362 | 2.14199 | H | -2.8619 | -4.51053 | 0.92237 |
| C | -2.44268 | 0.01642 | 0.16512 | H | -1.42528 | 2.33512 | 0.88759 |
| C | -3.13814 | -1.17889 | -0.06331 | H | -2.1908 | 3.14438 | -1.96077 |
| C | -4.43883 | -1.0837 | -0.55983 | H | -0.83805 | 2.0852 | -1.5067 |
| C | -5.01771 | 0.15439 | -0.80907 | H | -0.83097 | 3.79843 | -1.02336 |
| C | -4.30646 | 1.32353 | -0.56963 | H | -3.59162 | 3.45248 | 1.54693 |
| C | -3.00262 | 1.27909 | -0.07409 | H | -3.72786 | 4.09312 | -0.10149 |
| C | -2.46758 | -2.52679 | 0.15218 | H | -2.36096 | 4.56013 | 0.90915 |
| C | -1.76989 | -2.96837 | -1.14364 | H | 1.06153 | 2.4741 | 0.31303 |
| C | -3.42552 | -3.6086 | 0.66076 | H | 2.39013 | 3.61799 | 0.05558 |
| C | -2.19091 | 2.54828 | 0.13308 | H | 2.39231 | 2.57446 | 1.49089 |
| C | -1.46538 | 2.91647 | -1.17071 | H | 4.57746 | 2.93006 | -0.7445 |
| C | -3.0222 | 3.7257 | 0.65222 | H | 5.04546 | 1.23332 | -0.89713 |
| C | 2.93353 | 1.57614 | -0.38876 | H | 4.78999 | 1.92932 | 0.71274 |
| C | 2.14009 | 2.61675 | 0.42622 | H | 3.12543 | 1.02943 | -2.49719 |
| C | 4.42976 | 1.92988 | -0.31935 | H | 2.43037 | 2.62518 | -2.20216 |
| C | 2.44875 | 1.58847 | -1.84377 | H | 1.44155 | 1.17168 | -1.93623 |
| C | 3.04685 | -1.58025 | -0.5073 | H | 4.89627 | -0.88813 | -1.46076 |
| C | 4.56451 | -1.65184 | -0.74905 | H | 4.81161 | -2.6291 | -1.18076 |
| C | 2.29246 | -1.69701 | -1.83928 | H | 5.12622 | -1.53242 | 0.18228 |
| C | 2.62413 | -2.74378 | 0.41304 | H | 1.22904 | -1.47308 | -1.72263 |
| H | -1.58888 | -0.10617 | 2.79346 | H | 2.39912 | -2.72389 | -2.21046 |
| H | 1.19713 | -0.18158 | 2.98752 | H | 2.69945 | -1.03032 | -2.60284 |
| H | -5.00727 | -1.9871 | -0.75704 | H | 2.92326 | -3.68932 | -0.0544 |
| H | -6.03106 | 0.20897 | -1.19642 | H | 1.5381 | -2.76565 | 0.55275 |
| H | -4.77352 | 2.28226 | -0.77165 | H | 3.10351 | -2.67473 | 1.39381 |

**Supplementary Table 3.** Computed Cartesian coordinates (x, y, z) for the optimized structure of **1a**.

E1 = -3632.150367, G1 = -3631.503686, E2 = -3633.08234383

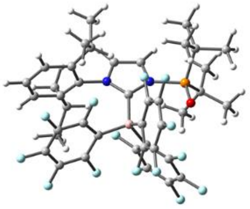

| | | | | | | | |
|---|---|---|---|---|---|---|---|
| P | -3.07657 | -0.64888 | -1.08138 | C | -0.18082 | -0.61027 | 3.07765 |
| F | 0.29315 | 2.58105 | -1.02104 | C | -0.43243 | -1.52152 | 4.09371 |
| F | -0.82963 | 4.94999 | -0.68156 | C | -0.80079 | -2.82105 | 3.77195 |
| F | -2.74045 | 5.29663 | 1.25817 | C | -0.88581 | -3.18287 | 2.43677 |
| F | -3.44746 | 3.17522 | 2.8097 | C | -0.61367 | -2.23247 | 1.46572 |
| F | -2.3005 | 0.85484 | 2.54561 | C | 1.68958 | 0.4805 | 0.87003 |
| F | -0.60975 | -2.67355 | 0.19288 | C | 2.33731 | 1.68164 | 1.15491 |
| F | -1.20682 | -4.43422 | 2.09107 | C | 3.70089 | 1.78976 | 1.41716 |
| F | -1.05295 | -3.70949 | 4.73164 | C | 4.49373 | 0.65763 | 1.46246 |
| F | -0.32192 | -1.16142 | 5.37329 | C | 3.89415 | -0.57327 | 1.2536 |
| F | 0.19304 | 0.62131 | 3.45646 | C | 2.53734 | -0.62465 | 0.9808 |
| F | 2.0499 | -1.8608 | 0.7844 | B | 0.066 | 0.21003 | 0.59284 |
| F | 4.6183 | -1.69254 | 1.30061 | H | 0.70041 | -0.3462 | -4.18217 |
| F | 5.79972 | 0.74683 | 1.69898 | H | -2.01266 | -0.40461 | -3.76727 |
| F | 4.24438 | 2.99075 | 1.61898 | H | 4.45572 | -2.95869 | -1.72709 |
| F | 1.69383 | 2.85819 | 1.18148 | H | 5.97616 | -1.02909 | -1.88947 |
| O | -3.07619 | -0.70026 | 0.40067 | H | 5.07386 | 1.22226 | -2.33583 |
| N | 0.73099 | -0.29697 | -2.03561 | H | 0.78722 | -2.71625 | -1.46451 |
| N | -1.40576 | -0.35623 | -1.68822 | H | 2.59276 | -3.8793 | -0.07986 |
| C | -0.19438 | -0.25351 | -1.03331 | H | 3.09845 | -4.69874 | -1.57006 |
| C | 0.12051 | -0.3412 | -3.2742 | H | 1.46362 | -4.93022 | -0.95019 |
| C | -1.20283 | -0.37378 | -3.06123 | H | 2.43805 | -3.76865 | -3.8199 |
| C | 2.17491 | -0.47873 | -2.00213 | H | 1.03906 | -2.69303 | -3.95973 |
| C | 2.66026 | -1.79576 | -1.89072 | H | 0.82573 | -4.34118 | -3.352 |
| C | 4.04353 | -1.96363 | -1.85194 | H | 1.42646 | 1.98065 | -2.64857 |
| C | 4.90312 | -0.87809 | -1.95463 | H | 4.1606 | 3.23941 | -2.10034 |
| C | 4.39263 | 0.39115 | -2.18136 | H | 2.8296 | 3.1349 | -0.9342 |
| C | 3.01658 | 0.61618 | -2.25033 | H | 2.6406 | 4.08556 | -2.40508 |
| C | 1.74285 | -3.01021 | -1.89801 | H | 2.42973 | 1.22831 | -4.82119 |
| C | 2.26345 | -4.19257 | -1.07134 | H | 3.9115 | 2.1045 | -4.42376 |
| C | 1.49562 | -3.47373 | -3.34427 | H | 2.39736 | 2.99568 | -4.66162 |
| C | 2.50968 | 1.9583 | -2.75176 | H | -1.88504 | -3.50994 | -1.3823 |
| C | 3.07244 | 3.16627 | -1.99591 | H | -3.42669 | -4.37237 | -1.37411 |
| C | 2.82951 | 2.07587 | -4.25276 | H | -2.98768 | -3.33202 | -0.00631 |
| C | -3.64653 | -2.2869 | -1.82035 | H | -5.48666 | -3.3979 | -1.66914 |
| C | -2.9327 | -3.43536 | -1.09104 | H | -5.77332 | -1.70125 | -2.04147 |
| C | -5.14632 | -2.37831 | -1.4567 | H | -5.31283 | -2.18699 | -0.39251 |
| C | -3.46089 | -2.51779 | -3.32853 | H | -3.8957 | -1.73968 | -3.95957 |
| C | -4.077 | 0.79553 | -1.75982 | H | -3.96355 | -3.45738 | -3.58612 |
| C | -5.26294 | 0.96214 | -0.78362 | H | -2.40563 | -2.63973 | -3.58893 |
| C | -4.61039 | 0.65689 | -3.19408 | H | -5.9788 | 0.13866 | -0.83676 |
| C | -3.2405 | 2.07685 | -1.67387 | H | -5.79582 | 1.88362 | -1.04562 |
| C | -0.84558 | 1.58505 | 0.78542 | H | -4.91294 | 1.04588 | 0.24807 |
| C | -1.85878 | 1.80405 | 1.72429 | H | -3.81373 | 0.62172 | -3.94378 |
| C | -2.49494 | 3.0351 | 1.88686 | H | -5.20727 | 1.54891 | -3.41626 |
| C | -2.14066 | 4.11971 | 1.10071 | H | -5.2626 | -0.20689 | -3.33444 |
| C | -1.16839 | 3.94459 | 0.12874 | H | -3.83607 | 2.91227 | -2.05895 |
| C | -0.58726 | 2.69588 | -0.01033 | H | -2.31102 | 2.03357 | -2.24896 |
| C | -0.30629 | -0.91212 | 1.72451 | H | -3.00197 | 2.2947 | -0.63630 |

**Supplementary Table 4.** Computed Cartesian coordinates (x, y, z) for the optimized structure of **3aB$^{1}$**.

E1 = -3632.107711, G1 = -3631.470267, $\nu_{imag}$ = 16.3$i$ cm$^{-1}$ , E2 = -3633.034084

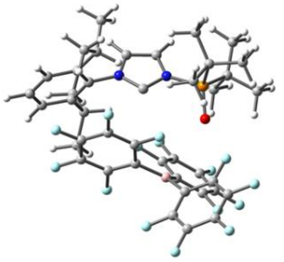

| | | | | | | | |
|---|---|---|---|---|---|---|---|
| P | -0.13714 | 2.6722 | 1.40295 | C | 3.73741 | -2.54199 | -0.49014 |
| F | -0.61682 | 0.30191 | -1.86936 | C | 4.90266 | -3.04831 | 0.069 |
| F | -0.79411 | 2.60594 | -3.12901 | C | 5.55294 | -2.30927 | 1.04793 |
| F | 1.44917 | 4.04023 | -3.68298 | C | 5.0389 | -1.0801 | 1.44526 |
| F | 3.8901 | 3.07742 | -2.97934 | C | 3.88038 | -0.59954 | 0.85131 |
| F | 4.10332 | 0.75431 | -1.72546 | C | 0.62572 | -1.82174 | -0.50947 |
| F | 3.41652 | 0.58142 | 1.25969 | C | -0.17819 | -2.3744 | -1.50267 |
| F | 5.66236 | -0.37649 | 2.38826 | C | -1.20651 | -3.26448 | -1.23501 |
| F | 6.66605 | -2.77449 | 1.60382 | C | -1.4411 | -3.6494 | 0.08014 |
| F | 5.39511 | -4.22213 | -0.31993 | C | -0.6606 | -3.12511 | 1.09991 |
| F | 3.13293 | -3.27014 | -1.44 | C | 0.35716 | -2.23641 | 0.7919 |
| F | 1.09304 | -1.78391 | 1.81025 | B | 1.83727 | -0.86309 | -0.79606 |
| F | -0.86799 | -3.50095 | 2.36226 | H | -4.84873 | 1.91113 | 0.07257 |
| F | -2.38139 | -4.54315 | 0.36061 | H | -3.03042 | 3.8188 | 0.80412 |
| F | -1.93614 | -3.77782 | -2.22271 | H | -5.02708 | -3.4474 | 1.29721 |
| F | 0.0536 | -2.07286 | -2.7857 | H | -5.53456 | -3.88328 | -1.06603 |
| O | 0.9785 | 2.02101 | 0.65559 | H | -4.88732 | -2.25673 | -2.80154 |
| N | -3.12636 | 0.61058 | 0.30804 | H | -2.77024 | -0.84636 | 2.44312 |
| N | -1.6898 | 2.08329 | 0.86308 | H | -3.18947 | -3.31937 | 2.85717 |
| C | -1.8187 | 0.72279 | 0.6572 | H | -4.81501 | -2.89718 | 3.43354 |
| C | -3.80096 | 1.82704 | 0.31448 | H | -3.39044 | -2.28034 | 4.27323 |
| C | -2.90052 | 2.75911 | 0.67474 | H | -5.78118 | -0.57618 | 2.9371 |
| C | -3.75326 | -0.62522 | -0.06388 | H | -4.6874 | 0.74022 | 2.47126 |
| C | -4.09981 | -1.54161 | 0.94518 | H | -4.51189 | -0.0358 | 4.05196 |
| C | -4.75128 | -2.70943 | 0.55275 | H | -2.95118 | 0.86689 | -2.08586 |
| C | -5.03638 | -2.96037 | -0.78499 | H | -3.64887 | -1.12461 | -4.29383 |
| C | -4.6724 | -2.04471 | -1.75872 | H | -2.13195 | -1.16093 | -3.38394 |
| C | -4.02085 | -0.85579 | -1.42007 | H | -2.54772 | 0.25505 | -4.36174 |
| C | -3.78402 | -1.26207 | 2.40963 | H | -5.38776 | 1.43883 | -2.15427 |
| C | -3.79664 | -2.5187 | 3.28486 | H | -5.63243 | 0.22705 | -3.41886 |
| C | -4.74695 | -0.21695 | 2.99681 | H | -4.62336 | 1.65091 | -3.73978 |
| C | -3.65392 | 0.14396 | -2.50635 | H | -0.46995 | 0.13282 | 2.70731 |
| C | -2.95469 | -0.51794 | -3.70108 | H | 0.19058 | 0.43011 | 4.33584 |
| C | -4.89753 | 0.91168 | -2.97914 | H | 1.22595 | 0.57708 | 2.89351 |
| C | -0.14782 | 2.24173 | 3.21245 | H | 1.02575 | 2.6216 | 4.97839 |
| C | 0.22204 | 0.74869 | 3.2866 | H | 0.69142 | 4.09597 | 4.06992 |
| C | 0.93305 | 3.0338 | 3.96667 | H | 1.90963 | 2.93818 | 3.48121 |
| C | -1.52631 | 2.46141 | 3.8543 | H | -1.88012 | 3.49349 | 3.76384 |
| C | -0.13013 | 4.50914 | 1.05529 | H | -1.45848 | 2.22918 | 4.92375 |
| C | 1.33893 | 4.96283 | 1.20504 | H | -2.28089 | 1.80056 | 3.41626 |
| C | -1.00935 | 5.3808 | 1.96467 | H | 1.68562 | 4.93026 | 2.24048 |
| C | -0.50615 | 4.71157 | -0.42645 | H | 1.42076 | 6.00078 | 0.86097 |
| C | 1.75053 | 0.39172 | -1.70774 | H | 2.0038 | 4.34163 | 0.59994 |
| C | 2.87468 | 1.16333 | -2.03545 | H | -2.07164 | 5.12641 | 1.9291 |
| C | 2.7926 | 2.37857 | -2.69881 | H | -0.91487 | 6.42593 | 1.64627 |
| C | 1.54821 | 2.86885 | -3.0703 | H | -0.6922 | 5.3308 | 3.00985 |
| C | 0.40632 | 2.13401 | -2.78851 | H | -0.38179 | 5.77115 | -0.67889 |
| C | 0.5239 | 0.92811 | -2.12098 | H | -1.53263 | 4.42719 | -0.66639 |
| C | 3.19704 | -1.31467 | -0.12567 | H | 0.15994 | 4.13439 | -1.07087 |

**Supplementary Table 5.** Computed Cartesian coordinates (x, y, z) for the optimized structure of **TS1a**.

E1 = -3632.131637, G1 = -3631.489347, E2 = -3633.062887

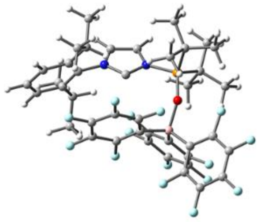

| | | | | | | | |
|---|---|---|---|---|---|---|---|
| P | 0.37913 | -0.11904 | 2.4473 | C | 4.46795 | -1.50782 | -2.43663 |
| F | -0.71272 | 1.90586 | -0.26791 | C | 5.22863 | -2.21955 | -1.51847 |
| F | -0.54818 | 4.52499 | -0.48082 | C | 4.83831 | -2.23722 | -0.1891 |
| F | 1.88795 | 5.74511 | -0.69529 | C | 3.69419 | -1.54965 | 0.20231 |
| F | 4.14695 | 4.22141 | -0.68072 | C | 0.31802 | -0.75698 | -1.26639 |
| F | 4.01308 | 1.60469 | -0.49347 | C | -0.51263 | -0.14716 | -2.20125 |
| F | 3.41347 | -1.61257 | 1.51967 | C | -1.63755 | -0.75806 | -2.74286 |
| F | 5.56243 | -2.90928 | 0.70869 | C | -1.93245 | -2.07012 | -2.40926 |
| F | 6.32254 | -2.86983 | -1.90901 | C | -1.06697 | -2.75888 | -1.57448 |
| F | 4.83482 | -1.46936 | -3.71723 | C | 0.04303 | -2.10626 | -1.06267 |
| F | 2.6494 | -0.14405 | -2.92163 | B | 1.49436 | -0.06076 | -0.35998 |
| F | 0.86876 | -2.85741 | -0.31431 | H | -4.19684 | 1.69072 | 2.22964 |
| F | -1.3044 | -4.03986 | -1.27389 | H | -1.85453 | 1.78059 | 3.65771 |
| F | -3.00379 | -2.67755 | -2.91795 | H | -5.87527 | -2.47577 | -1.32301 |
| F | -2.42235 | -0.0961 | -3.5954 | H | -6.58737 | -0.64533 | -2.80073 |
| F | -0.2722 | 1.09807 | -2.642 | H | -5.62952 | 1.60992 | -2.54018 |
| O | 1.12903 | -0.38444 | 1.15037 | H | -3.01598 | -2.29255 | 0.89441 |
| N | -3.05987 | 0.2919 | 1.02031 | H | -3.91756 | -3.90512 | -0.79356 |
| N | -1.22832 | 0.35785 | 2.10321 | H | -5.419 | -4.02836 | 0.15083 |
| C | -1.82248 | -0.24817 | 1.01051 | H | -3.88414 | -4.53976 | 0.85701 |
| C | -3.26121 | 1.17557 | 2.07827 | H | -5.84048 | -2.34312 | 2.07526 |
| C | -2.10874 | 1.22417 | 2.77149 | H | -4.53022 | -1.27692 | 2.60906 |
| C | -4.02077 | 0.03382 | -0.01909 | H | -4.39044 | -3.03003 | 2.83166 |
| C | -4.53586 | -1.26865 | -0.15747 | H | -2.98471 | 2.47567 | -0.03208 |
| C | -5.46305 | -1.48433 | -1.17399 | H | -3.929 | 3.07082 | -2.87023 |
| C | -5.86318 | -0.45171 | -2.01523 | H | -2.42853 | 2.23279 | -2.4436 |
| C | -5.32661 | 0.81574 | -1.8643 | H | -2.67519 | 3.91221 | -1.94492 |
| C | -4.3819 | 1.08642 | -0.87057 | H | -5.28974 | 3.18555 | 0.67996 |
| C | -4.09802 | -2.3945 | 0.77042 | H | -5.66939 | 3.57883 | -1.00033 |
| C | -4.34925 | -3.79371 | 0.20428 | H | -4.4031 | 4.48089 | -0.15013 |
| C | -4.75109 | -2.24797 | 2.15332 | H | -0.94898 | -2.55267 | 1.73406 |
| C | -3.79412 | 2.48635 | -0.76364 | H | -0.43826 | -3.70426 | 2.98755 |
| C | -3.17187 | 2.94773 | -2.08774 | H | 0.73582 | -3.07508 | 1.81511 |
| C | -4.8504 | 3.48898 | -0.27736 | H | 1.33683 | -3.08128 | 4.58328 |
| C | 0.18974 | -1.69734 | 3.41181 | H | 1.73592 | -1.40959 | 4.969 |
| C | -0.13601 | -2.82066 | 2.41365 | H | 2.32978 | -2.14669 | 3.46201 |
| C | 1.48297 | -2.08607 | 4.14884 | H | -0.87894 | -0.73784 | 5.10584 |
| C | -0.9854 | -1.56516 | 4.39964 | H | -1.0351 | -2.48895 | 4.98638 |
| C | 1.21264 | 1.25099 | 3.39898 | H | -1.93913 | -1.45349 | 3.87573 |
| C | 2.7331 | 0.99318 | 3.31607 | H | 3.03955 | 0.0755 | 3.82181 |
| C | 0.78368 | 1.3128 | 4.87703 | H | 3.24259 | 1.83256 | 3.80257 |
| C | 0.94324 | 2.62107 | 2.7444 | H | 3.07658 | 0.94044 | 2.28045 |
| C | 1.62631 | 1.57227 | -0.47497 | H | -0.28025 | 1.52972 | 5.00771 |
| C | 2.84264 | 2.2535 | -0.53844 | H | 1.33899 | 2.12737 | 5.35449 |
| C | 2.9497 | 3.64064 | -0.62011 | H | 1.01399 | 0.39944 | 5.42771 |
| C | 1.80441 | 4.42051 | -0.62716 | H | 1.29873 | 3.39875 | 3.4291 |
| C | 0.56948 | 3.79575 | -0.53515 | H | -0.11056 | 2.8164 | 2.5318 |
| C | 0.51452 | 2.41438 | -0.45013 | H | 1.50297 | 2.72112 | 1.8166 |
| C | 2.89388 | -0.83602 | -0.67827 | | | | |
| C | 3.33394 | -0.84001 | -2.00026 | | | | |

**Supplementary Table 6.** Computed Cartesian coordinates (x, y, z) for the optimized structure of **2a'B[1]**.

E1 = -3632.110439, G1 = -3631.465385, $\nu_{imag}$ = 19.9$i$ $cm^{-1}$ ,E2 = -3633.041473

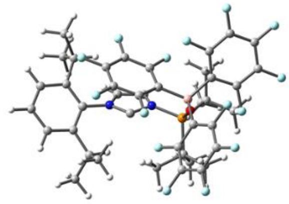

| | | | |
|---|---|---|---|
| P | -0.53435 | 0.23664 | 0.00000 |
| F | -0.64251 | 2.99309 | -2.14106 |
| F | 0.27448 | 5.49131 | -2.21585 |
| F | 2.78402 | 5.99702 | -3.18315 |
| F | 4.32317 | 3.92484 | -4.04981 |
| F | 3.44065 | 1.44257 | -3.94883 |
| F | 3.12857 | 0.43156 | -1.19402 |
| F | 4.91509 | -1.53619 | -1.10014 |
| F | 4.763 | -3.61921 | -2.86063 |
| F | 2.76044 | -3.66875 | -4.70577 |
| F | 0.95185 | -1.74585 | -4.79537 |
| F | -0.86242 | -1.82468 | -2.68951 |
| F | -3.15002 | -2.30137 | -3.90413 |
| F | -4.27731 | -0.38873 | -5.53822 |
| F | -2.95832 | 1.94864 | -5.94036 |
| F | -0.61488 | 2.39193 | -4.84763 |
| O | 0.33439 | 0.42411 | -1.23578 |
| N | -4.13746 | 0.0984 | -1.13406 |
| N | -2.12946 | -0.22979 | -0.44344 |
| C | -2.88369 | 0.61506 | -1.24944 |
| C | -4.18895 | -0.96579 | -0.2326 |
| C | -2.93988 | -1.18298 | 0.20336 |
| C | -5.26775 | 0.57147 | -1.90292 |
| C | -6.09535 | -0.35365 | -2.58092 |
| C | -7.15183 | 0.15188 | -3.34056 |
| C | -7.38382 | 1.51222 | -3.45713 |
| C | -6.56672 | 2.39885 | -2.77753 |
| C | -5.5129 | 1.95759 | -1.97645 |
| C | -5.95796 | -1.87261 | -2.50565 |
| C | -6.14777 | -2.5719 | -3.86055 |
| C | -6.97882 | -2.44767 | -1.50654 |
| C | -4.69328 | 3.01023 | -1.24914 |
| C | -3.77684 | 3.7464 | -2.23375 |
| C | -5.56885 | 3.98485 | -0.45034 |
| C | 0.20689 | -1.22684 | 0.91124 |
| C | 0.32178 | -2.3893 | -0.09545 |
| C | 1.64253 | -0.83021 | 1.32233 |
| C | -0.55348 | -1.72698 | 2.17314 |
| C | -0.56276 | 1.77681 | 1.06723 |
| C | 0.79017 | 2.48725 | 0.86766 |
| C | -0.75592 | 1.44446 | 2.55847 |
| C | -1.71454 | 2.71651 | 0.68006 |
| C | 1.36839 | 2.07305 | -2.95277 |
| C | 2.62005 | 2.38843 | -3.47382 |
| C | 3.10817 | 3.69069 | -3.5546 |
| C | 2.32792 | 4.74926 | -3.11831 |
| C | 1.05741 | 4.49029 | -2.62254 |
| C | 0.61693 | 3.17891 | -2.57015 |
| C | 1.88824 | -0.59269 | -2.93632 |
| C | 1.88353 | -1.6499 | -3.83959 |
| C | 2.83199 | -2.67026 | -3.82609 |
| C | 3.85466 | -2.64782 | -2.89205 |
| C | 3.92694 | -1.58999 | -1.99472 |
| C | 2.96828 | -0.59294 | -2.05533 |
| C | -0.61437 | 0.32378 | -3.65327 |
| C | -1.204 | 1.23292 | -4.52774 |
| C | -2.43621 | 1.02109 | -5.13891 |
| C | -3.11332 | -0.16735 | -4.92992 |
| C | -2.54363 | -1.1265 | -4.10617 |
| C | -1.3352 | -0.85506 | -3.49163 |
| B | 0.74851 | 0.56942 | -2.79289 |
| H | -5.10078 | -1.47541 | 0.02422 |
| H | -2.57227 | -1.94002 | 0.87038 |
| H | -7.79916 | -0.54083 | -3.86754 |
| H | -8.19926 | 1.87779 | -4.07369 |
| H | -6.74784 | 3.46561 | -2.86536 |
| H | -4.94774 | -2.12136 | -2.17305 |
| H | -5.53984 | -2.1132 | -4.63911 |
| H | -7.19427 | -2.55665 | -4.18411 |
| H | -5.85332 | -3.62265 | -3.76961 |
| H | -7.99556 | -2.30603 | -1.89023 |
| H | -6.94521 | -1.95978 | -0.52641 |
| H | -6.81823 | -3.52214 | -1.36424 |
| H | -4.05248 | 2.49959 | -0.53423 |
| H | -4.36248 | 4.26591 | -3.00128 |
| H | -3.10633 | 3.03663 | -2.72371 |
| H | -3.16552 | 4.49237 | -1.71287 |
| H | -6.2496 | 3.4505 | 0.22103 |
| H | -6.17179 | 4.63122 | -1.09741 |
| H | -4.93134 | 4.63788 | 0.15651 |
| H | -0.65104 | -2.72228 | -0.46443 |
| H | 0.79822 | -3.23161 | 0.4187 |
| H | 0.93625 | -2.12185 | -0.95623 |
| H | 2.1597 | -1.73816 | 1.65148 |
| H | 1.66005 | -0.12685 | 2.15889 |
| H | 2.21069 | -0.40311 | 0.49718 |
| H | -1.33782 | -1.06425 | 2.53766 |
| H | 0.15622 | -1.86153 | 2.99506 |
| H | -1.00822 | -2.70480 | 1.98688 |
| H | 1.63916 | 1.87728 | 1.18458 |
| H | 0.78778 | 3.40097 | 1.47231 |
| H | 0.95266 | 2.76674 | -0.17266 |
| H | -1.73008 | 0.98371 | 2.75029 |
| H | -0.73575 | 2.39181 | 3.10826 |
| H | 0.02614 | 0.80850 | 2.97673 |
| H | -1.58296 | 3.65397 | 1.23300 |
| H | -2.67763 | 2.28984 | 0.97350 |
| H | -1.7411 | 2.94139 | -0.38210 |

**Supplementary Table 7.** Computed Cartesian coordinates (x, y, z) for the optimized structure of **TS2a**.

E1 = -3632.138152, G1 = -3631.497191, E2 = -3633.06490

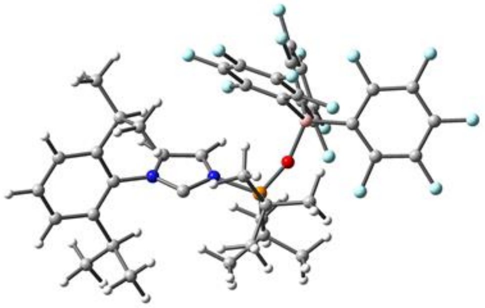

| | | | |
|---|---|---|---|
| P | 0.45919 | -1.39893 | -1.0866 |
| F | 0.30563 | 1.41541 | 1.59481 |
| F | 1.53911 | 3.67033 | 1.02445 |
| F | 0.85473 | 5.11121 | -1.181 |
| F | -1.20712 | 4.25039 | -2.75486 |
| F | -2.47062 | 2.00865 | -2.21267 |
| F | -2.89587 | -2.15369 | -1.76468 |
| F | -5.36394 | -2.36457 | -2.65274 |
| F | -7.23594 | -0.46979 | -2.05802 |
| F | -6.53008 | 1.67082 | -0.52148 |
| F | -4.03326 | 1.91018 | 0.38698 |
| F | -2.58063 | -2.46053 | 1.12931 |
| F | -3.09182 | -3.11516 | 3.64977 |
| F | -3.11749 | -1.19935 | 5.5954 |
| F | -2.60131 | 1.38985 | 4.92853 |
| F | -2.05189 | 2.06291 | 2.43858 |
| O | -0.85688 | -0.99472 | -0.40592 |
| N | 3.75869 | -0.41358 | 0.43649 |
| N | 1.75564 | -0.82975 | -0.12738 |
| C | 3.00723 | -0.59385 | -0.6713 |
| C | 3.03832 | -0.557 | 1.62416 |
| C | 1.7697 | -0.83192 | 1.27558 |
| C | 5.15281 | -0.06667 | 0.40407 |
| C | 5.50138 | 1.28951 | 0.47979 |
| C | 6.86144 | 1.60132 | 0.44788 |
| C | 7.82261 | 0.60327 | 0.34906 |
| C | 7.44548 | -0.73178 | 0.28 |
| C | 6.09885 | -1.09589 | 0.30996 |
| C | 4.44262 | 2.38174 | 0.5211 |
| C | 4.10103 | 2.82299 | -0.91081 |
| C | 4.84457 | 3.5842 | 1.38103 |
| C | 5.67343 | -2.54912 | 0.17479 |
| C | 5.5606 | -2.91686 | -1.3129 |
| C | 6.59422 | -3.52473 | 0.91511 |
| C | 0.6047 | -3.25279 | -0.96818 |
| C | 0.29569 | -3.65287 | 0.48786 |
| C | -0.40909 | -3.94232 | -1.89463 |
| C | 2.03394 | -3.72451 | -1.28978 |
| C | 0.63528 | -0.67544 | -2.79385 |
| C | -0.77695 | -0.66972 | -3.41333 |
| C | 1.58713 | -1.48485 | -3.69064 |
| C | 1.17894 | 0.76597 | -2.72378 |
| C | -1.17021 | 1.56822 | -0.26182 |
| C | -1.5057 | 2.36963 | -1.3535 |
| C | -0.85155 | 3.55496 | -1.67625 |
| C | 0.19499 | 3.99644 | -0.88248 |
| C | 0.55213 | 3.25052 | 0.22968 |
| C | -0.11996 | 2.07043 | 0.50621 |
| C | -3.32075 | -0.0902 | -0.65135 |
| C | -4.32282 | 0.83898 | -0.3677 |
| C | -5.62703 | 0.73958 | -0.82513 |
| C | -5.99031 | -0.34983 | -1.60684 |
| C | -5.03713 | -1.30767 | -1.90737 |
| C | -3.73978 | -1.16384 | -1.42365 |
| C | -2.15243 | -0.1855 | 1.65852 |
| C | -2.24628 | 0.76167 | 2.67754 |
| C | -2.5551 | 0.44004 | 3.99557 |
| C | -2.82522 | -0.87587 | 4.33892 |
| C | -2.80057 | -1.84874 | 3.35058 |
| C | -2.49702 | -1.47979 | 2.04747 |
| B | -1.86396 | 0.11965 | 0.06936 |
| H | 3.49676 | -0.42121 | 2.59086 |
| H | 0.87883 | -0.97367 | 1.86523 |
| H | 7.17581 | 2.63867 | 0.49859 |
| H | 8.8756 | 0.86818 | 0.32473 |
| H | 8.20918 | -1.49896 | 0.20143 |
| H | 3.53055 | 1.96471 | 0.95999 |
| H | 3.7708 | 1.96908 | -1.50971 |
| H | 4.98066 | 3.25983 | -1.39793 |
| H | 3.30661 | 3.57617 | -0.89978 |
| H | 5.6679 | 4.15095 | 0.93223 |
| H | 5.15084 | 3.2757 | 2.38618 |
| H | 3.99493 | 4.26601 | 1.47462 |
| H | 4.67672 | -2.64868 | 0.62065 |
| H | 6.53667 | -2.8256 | -1.80344 |
| H | 4.85742 | -2.24873 | -1.82004 |
| H | 5.21363 | -3.95016 | -1.43174 |
| H | 6.73719 | -3.22993 | 1.95974 |
| H | 7.58024 | -3.59476 | 0.44264 |
| H | 6.15762 | -4.52898 | 0.89937 |
| H | 1.06211 | -3.29225 | 1.18 |
| H | 0.29829 | -4.74732 | 0.54152 |
| H | -0.68293 | -3.30186 | 0.81212 |
| H | -0.29249 | -5.02578 | -1.7803 |
| H | -0.24586 | -3.70754 | -2.95024 |
| H | -1.43423 | -3.68321 | -1.63112 |
| H | 2.32296 | -3.56107 | -2.32806 |
| H | 2.0778 | -4.80297 | -1.09975 |
| H | 2.77654 | -3.24367 | -0.64817 |
| H | -1.24363 | -1.65659 | -3.42729 |
| H | -0.68017 | -0.32749 | -4.44939 |
| H | -1.45004 | 0.01766 | -2.89895 |
| H | 2.58617 | -1.54289 | -3.25255 |
| H | 1.66658 | -0.9538 | -4.64595 |
| H | 1.21616 | -2.48972 | -3.90841 |
| H | 0.90612 | 1.27634 | -3.65404 |
| H | 2.26347 | 0.75415 | -2.62237 |
| H | 0.77085 | 1.33976 | -1.89384 |

**Supplementary Table 8.** Computed Cartesian coordinates (x, y, z) for the optimized structure of **2aB[1]**.

E1 = -1306.58334, G1 = -1306.156539, E2 = -1306.864588

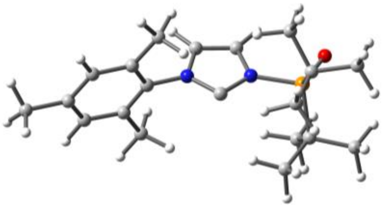

| | | | |
|---|---|---|---|
| P | -2.60302100 | -0.01870800 | 0.38368700 |
| O | -3.40038000 | -0.08922100 | 1.64719100 |
| N | 1.20165500 | -0.11808600 | 0.73334100 |
| N | -0.91713700 | -0.13122100 | 0.79516600 |
| C | 0.12719300 | -0.00462000 | -0.09003700 |
| C | 0.85184900 | -0.29966400 | 2.07209800 |
| C | -0.49519000 | -0.30501100 | 2.11432100 |
| C | 2.55596400 | -0.04697500 | 0.27656000 |
| C | 3.13710800 | 1.20834200 | 0.07481600 |
| C | 4.46644100 | 1.25356300 | -0.33958400 |
| C | 5.20712500 | 0.09076400 | -0.55626600 |
| C | 4.58798000 | -1.14147000 | -0.35325300 |
| C | 3.25963700 | -1.23404900 | 0.06151400 |
| C | -2.90377300 | -1.50406500 | -0.69276200 |
| C | -2.59145900 | -2.71005800 | 0.21695300 |
| C | -4.39690300 | -1.52193200 | -1.06227600 |
| C | -2.04468600 | -1.60091500 | -1.96190000 |
| C | -2.77487200 | 1.64165300 | -0.43448700 |
| C | -4.27311300 | 1.99465400 | -0.43360800 |
| C | -2.20561900 | 1.71953600 | -1.85639100 |
| C | -2.03236500 | 2.64327900 | 0.47221100 |
| H | 1.59313100 | -0.40106500 | 2.84946500 |
| H | -1.19960300 | -0.40666500 | 2.92442200 |
| H | 4.93661600 | 2.22202600 | -0.49695300 |
| H | 5.15014700 | -2.05692500 | -0.52478200 |
| H | -1.52359900 | -2.76307700 | 0.45453400 |
| H | -2.86454000 | -3.63120500 | -0.31136500 |
| H | -3.15587600 | -2.66565400 | 1.15268500 |
| H | -4.63709000 | -2.48232100 | -1.53400600 |
| H | -4.64331300 | -0.73480200 | -1.78285400 |
| H | -5.03333500 | -1.40308000 | -0.18000300 |
| H | -2.35505500 | -0.88341200 | -2.72479900 |
| H | -2.16714500 | -2.60466400 | -2.38799700 |
| H | -0.98486800 | -1.43206400 | -1.75211900 |
| H | -4.85519700 | 1.33011800 | -1.07951700 |
| H | -4.39679600 | 3.01541500 | -0.81520900 |
| H | -4.69219400 | 1.94323100 | 0.57475800 |
| H | -1.18258600 | 1.33319900 | -1.89841900 |
| H | -2.19803100 | 2.76937900 | -2.17546700 |
| H | -2.82620100 | 1.16777600 | -2.56934000 |
| H | -2.25988600 | 3.66074100 | 0.13252100 |
| H | -0.94898300 | 2.50204400 | 0.42053700 |
| H | -2.34991700 | 2.55362500 | 1.51617200 |
| C | 2.59038800 | -2.56942100 | 0.25996300 |
| H | 1.65276800 | -2.62021300 | -0.30392600 |
| H | 3.24011300 | -3.38400900 | -0.07130700 |
| H | 2.34047800 | -2.74208500 | 1.31307500 |
| C | 2.33450100 | 2.46634800 | 0.28006400 |
| H | 1.52577300 | 2.52551600 | -0.45669000 |
| H | 1.86688400 | 2.48806500 | 1.27058200 |
| H | 2.96393500 | 3.35446900 | 0.17692100 |
| C | 6.63763400 | 0.17068300 | -1.02824300 |
| H | 7.18492400 | -0.74946200 | -0.80220000 |
| H | 6.68349400 | 0.32517400 | -2.11295500 |
| H | 7.16564700 | 1.00561400 | -0.55631700 |

**Supplementary Table 9.** Computed Cartesian coordinates (x, y, z) for the optimized structure of **1b**.

E1 = -3514.247507, G1 = -3513.683249, E2 = -3515.147462

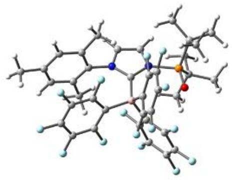

| | | | |
|---|---|---|---|
| P | -3.14312 | -0.58985 | -1.00253 |
| F | -0.14466 | 2.63213 | -1.0971 |
| F | -1.33282 | 4.88129 | -0.39256 |
| F | -2.80644 | 4.99545 | 1.91973 |
| F | -3.03406 | 2.75488 | 3.45028 |
| F | -1.83182 | 0.53811 | 2.79974 |
| F | -0.66518 | -2.64962 | -0.30661 |
| F | -0.85554 | -4.68117 | 1.38228 |
| F | -0.25145 | -4.34562 | 4.03251 |
| F | 0.54754 | -1.89153 | 4.91535 |
| F | 0.71357 | 0.16229 | 3.22846 |
| F | 2.28273 | -1.82063 | 0.32987 |
| F | 4.85941 | -1.49528 | 0.65455 |
| F | 5.92428 | 0.98446 | 1.03123 |
| F | 4.24804 | 3.14397 | 1.12298 |
| F | 1.68437 | 2.87886 | 0.83346 |
| O | -3.06373 | -0.43879 | 0.47089 |
| N | 0.57912 | -0.14791 | -2.28066 |
| N | -1.5174 | -0.33013 | -1.7606 |
| C | -0.26396 | -0.15371 | -1.21242 |
| C | -0.11844 | -0.2876 | -3.4648 |
| C | -1.41545 | -0.40044 | -3.14398 |
| C | 2.02671 | -0.21029 | -2.35465 |
| C | 2.6026 | -1.48707 | -2.4158 |
| C | 3.99109 | -1.57175 | -2.4651 |
| C | 4.79533 | -0.43401 | -2.48768 |
| C | 4.17376 | 0.81018 | -2.55326 |
| C | 2.78747 | 0.95312 | -2.50959 |
| C | -3.72208 | -2.33382 | -1.49352 |
| C | -3.60716 | -3.20762 | -0.22565 |
| C | -5.21843 | -2.32683 | -1.86723 |
| C | -2.934 | -2.99095 | -2.63578 |
| C | -4.16587 | 0.78484 | -1.77337 |
| C | -5.53281 | 0.77847 | -1.05079 |
| C | -4.37092 | 0.76467 | -3.29897 |
| C | -3.4712 | 2.10044 | -1.3986 |
| C | -0.79615 | 1.47196 | 0.85295 |
| C | -1.61331 | 1.56704 | 1.98317 |
| C | -2.28247 | 2.73297 | 2.34868 |
| C | -2.1726 | 3.87715 | 1.57448 |
| C | -1.41287 | 3.82247 | 0.41812 |
| C | -0.78449 | 2.63321 | 0.09037 |
| C | -0.07248 | -1.11033 | 1.39453 |
| C | 0.27947 | -1.00736 | 2.7394 |
| C | 0.21718 | -2.06406 | 3.6359 |
| C | -0.18559 | -3.31634 | 3.19067 |
| C | -0.49762 | -3.48106 | 1.85178 |
| C | -0.42311 | -2.38693 | 1.00273 |
| C | 1.77646 | 0.50027 | 0.54543 |
| C | 2.38386 | 1.73498 | 0.77875 |
| C | 3.75487 | 1.91805 | 0.94318 |
| C | 4.60702 | 0.83023 | 0.90901 |
| C | 4.06134 | -0.42692 | 0.71595 |
| C | 2.69205 | -0.55934 | 0.54827 |
| B | 0.13767 | 0.17819 | 0.39909 |
| H | 0.39192 | -0.30521 | -4.41391 |
| H | -2.26729 | -0.54575 | -3.78153 |
| H | 4.45588 | -2.554 | -2.46213 |
| H | 4.78325 | 1.7077 | -2.6306 |
| H | -2.58105 | -3.49164 | -0.01515 |
| H | -4.17572 | -4.12832 | -0.40023 |
| H | -4.01641 | -2.70438 | 0.65239 |
| H | -5.50001 | -3.35563 | -2.11837 |
| H | -5.46033 | -1.70747 | -2.7329 |
| H | -5.84267 | -2.01894 | -1.02447 |
| H | -3.15217 | -2.53954 | -3.60947 |
| H | -3.23658 | -4.04247 | -2.69628 |
| H | -1.85519 | -2.96719 | -2.46709 |
| H | -6.22138 | 0.03097 | -1.44632 |
| H | -5.99835 | 1.75948 | -1.19887 |
| H | -5.41547 | 0.62434 | 0.02566 |
| H | -3.50415 | 1.17553 | -3.8238 |
| H | -5.22124 | 1.41241 | -3.5403 |
| H | -4.59219 | -0.22348 | -3.71315 |
| H | -4.06471 | 2.93518 | -1.78927 |
| H | -2.46817 | 2.17901 | -1.82525 |
| H | -3.40807 | 2.20764 | -0.31459 |
| C | 2.18925 | 2.32739 | -2.63506 |
| H | 2.07991 | 2.80997 | -1.66181 |
| H | 2.83841 | 2.95613 | -3.25096 |
| H | 1.19897 | 2.30472 | -3.09468 |
| C | 1.76591 | -2.73892 | -2.44306 |
| H | 1.00839 | -2.74404 | -1.6601 |
| H | 1.2518 | -2.8441 | -3.40675 |
| H | 2.39684 | -3.62034 | -2.30602 |
| C | 6.2938 | -0.542 | -2.39419 |
| H | 6.79094 | 0.2847 | -2.91063 |
| H | 6.59722 | -0.50726 | -1.34135 |
| H | 6.65913 | -1.48361 | -2.81465 |

**Supplementary Table 10.** Computed Cartesian coordinates (x, y, z) for the optimized structure of **3bB[1]**.

E1 = -3514.199582, $\nu_{imag}$ = 43.6$i$ cm$^{-1}$ , G1 = -3513.642864, E2 = -3515.094816

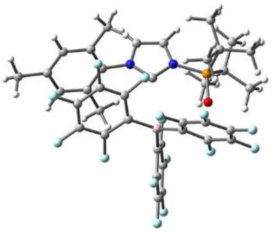

| | | | |
|---|---|---|---|
| P | -2.42515 | -1.93632 | -0.83602 |
| F | -0.62665 | 0.43736 | -2.4746 |
| F | -1.23414 | 2.26718 | -4.23966 |
| F | -1.28038 | 4.91292 | -3.57264 |
| F | -0.70448 | 5.65764 | -1.00954 |
| F | -0.11855 | 3.79231 | 0.82679 |
| F | -2.85717 | 1.91482 | 0.16655 |
| F | -4.7971 | 1.63976 | 1.89482 |
| F | -4.28071 | 0.88942 | 4.45642 |
| F | -1.71113 | 0.41355 | 5.22524 |
| F | 0.25477 | 0.62275 | 3.51864 |
| F | 0.9768 | -1.26136 | 2.01537 |
| F | 3.47115 | -1.65291 | 2.78423 |
| F | 5.42206 | 0.11228 | 2.10499 |
| F | 4.7994 | 2.29157 | 0.60129 |
| F | 2.31301 | 2.75591 | -0.13808 |
| O | -2.55668 | -0.51758 | -0.41265 |
| N | 1.35222 | -2.03198 | -1.35523 |
| N | -0.76512 | -2.25735 | -1.26136 |
| C | 0.25183 | -1.4519 | -0.79805 |
| C | 1.03956 | -3.14127 | -2.13657 |
| C | -0.29366 | -3.29007 | -2.0735 |
| C | 2.73133 | -1.67757 | -1.1576 |
| C | 3.50457 | -2.51258 | -0.34008 |
| C | 4.86441 | -2.23654 | -0.21367 |
| C | 5.45657 | -1.15926 | -0.87118 |
| C | 4.65541 | -0.35764 | -1.68176 |
| C | 3.29116 | -0.59683 | -1.84612 |
| C | -2.75193 | -3.11424 | 0.56182 |
| C | -2.0419 | -2.49368 | 1.77889 |
| C | -4.25956 | -3.16564 | 0.86177 |
| C | -2.19789 | -4.52558 | 0.31845 |
| C | -3.43823 | -2.26342 | -2.37246 |
| C | -4.77126 | -1.5177 | -2.14262 |
| C | -3.74647 | -3.73375 | -2.69378 |
| C | -2.73132 | -1.6056 | -3.57278 |
| C | -0.33746 | 2.02675 | -0.71645 |
| C | -0.38216 | 3.39064 | -0.42663 |
| C | -0.68573 | 4.37145 | -1.35416 |
| C | -0.97916 | 3.99509 | -2.6591 |
| C | -0.94983 | 2.65237 | -2.99392 |
| C | -0.62706 | 1.69907 | -2.03251 |
| C | -1.18039 | 1.08075 | 1.65675 |
| C | -0.96904 | 0.79539 | 3.01516 |
| C | -1.98713 | 0.70969 | 3.95546 |
| C | -3.29448 | 0.96351 | 3.5721 |
| C | -3.55239 | 1.33785 | 2.26267 |
| C | -2.50962 | 1.43111 | 1.35164 |
| C | 1.47436 | 0.8006 | 0.94347 |
| C | 2.51972 | 1.66231 | 0.6069 |
| C | 3.83961 | 1.45283 | 0.99105 |
| C | 4.16204 | 0.34433 | 1.75084 |
| C | 3.16241 | -0.55059 | 2.10034 |
| C | 1.86464 | -0.31671 | 1.68675 |
| B | -0.04101 | 1.093 | 0.54981 |
| H | 1.7979 | -3.70414 | -2.65822 |
| H | -0.93188 | -4.03056 | -2.52409 |
| H | 5.46984 | -2.86412 | 0.43551 |
| H | 5.10113 | 0.49087 | -2.19646 |
| H | -0.97729 | -2.33518 | 1.59637 |
| H | -2.14929 | -3.17037 | 2.63499 |
| H | -2.48557 | -1.52972 | 2.03813 |
| H | -4.41287 | -3.69986 | 1.80681 |
| H | -4.82843 | -3.69514 | 0.09166 |
| H | -4.67792 | -2.16035 | 0.97801 |
| H | -2.62708 | -5.00653 | -0.56573 |
| H | -2.44268 | -5.15372 | 1.18314 |
| H | -1.10855 | -4.51689 | 0.21384 |
| H | -5.36172 | -1.95858 | -1.3347 |
| H | -5.36635 | -1.57875 | -3.06139 |
| H | -4.59762 | -0.46548 | -1.9066 |
| H | -2.85552 | -4.32753 | -2.91688 |
| H | -4.37885 | -3.76544 | -3.58906 |
| H | -4.29637 | -4.2323 | -1.89133 |
| H | -3.39739 | -1.66468 | -4.44172 |
| H | -1.79402 | -2.10483 | -3.83463 |
| H | -2.51141 | -0.55335 | -3.3835 |
| C | 2.46902 | 0.30946 | -2.72113 |
| H | 2.04929 | 1.13223 | -2.13249 |
| H | 3.0915 | 0.75005 | -3.50507 |
| H | 1.6334 | -0.21338 | -3.18906 |
| C | 2.87643 | -3.65271 | 0.4213 |
| H | 1.99714 | -3.31152 | 0.97551 |
| H | 2.55019 | -4.45805 | -0.24628 |
| H | 3.58651 | -4.07196 | 1.13783 |
| C | 6.91321 | -0.82627 | -0.67673 |
| H | 7.42048 | -0.68706 | -1.63721 |
| H | 7.0151 | 0.10521 | -0.10969 |
| H | 7.43456 | -1.61354 | -0.125 |

**Supplementary Table 11.** Computed Cartesian coordinates (x, y, z) for the optimized structure of **TS1b**.

E1 = -3514.221921, G1 = -3513.665699, E2 = -3515.122036

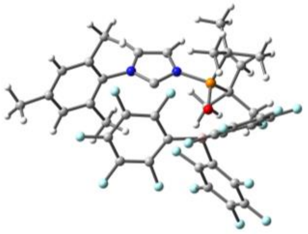

| | | | | | | | |
|---|---|---|---|---|---|---|---|
| P | 0.67184 | 0.24728 | 2.33175 | C | 3.82775 | -3.66831 | -0.84041 |
| F | -0.13311 | 2.45243 | -0.418 | C | 4.115 | -2.55377 | -0.06442 |
| F | 0.94412 | 4.85288 | -0.78544 | C | 3.27258 | -1.457 | -0.12652 |
| F | 3.45128 | 5.0886 | -1.85035 | C | -0.36944 | -0.35112 | -1.40291 |
| F | 4.83438 | 2.83963 | -2.52257 | C | -0.9611 | 0.43175 | -2.39086 |
| F | 3.79826 | 0.44439 | -2.137 | C | -2.25671 | 0.23215 | -2.86167 |
| F | 3.64993 | -0.39224 | 0.60903 | C | -3.0093 | -0.82286 | -2.37609 |
| F | 5.19091 | -2.54039 | 0.72565 | C | -2.44944 | -1.65977 | -1.42375 |
| F | 4.61973 | -4.73615 | -0.80441 | C | -1.16762 | -1.4068 | -0.9705 |
| F | 2.43401 | -4.69629 | -2.42936 | B | 1.11091 | -0.10737 | -0.76285 |
| F | 0.85163 | -2.59283 | -2.52208 | H | -3.96322 | 1.16521 | 3.74184 |
| F | -0.69102 | -2.27503 | -0.06792 | H | -1.34035 | 1.19576 | 4.56912 |
| F | -3.14983 | -2.69194 | -0.94163 | H | -6.5418 | -2.05077 | 0.22332 |
| F | -4.23757 | -1.04708 | -2.83938 | H | -5.8378 | 1.85468 | -1.38828 |
| F | -2.76606 | 1.0368 | -3.79827 | H | -0.3871 | -2.45155 | 2.25284 |
| F | -0.30355 | 1.44558 | -2.97513 | H | 0.90535 | -3.39109 | 3.02574 |
| O | 0.95695 | 0.06647 | 0.8589 | H | 1.20855 | -2.53613 | 1.49973 |
| N | -3.06095 | 0.38107 | 1.92809 | H | 2.8975 | -2.3463 | 3.91778 |
| N | -1.02568 | 0.41339 | 2.53973 | H | 3.04806 | -0.61313 | 4.19273 |
| C | -1.82802 | 0.08079 | 1.46824 | H | 3.21841 | -1.26842 | 2.55683 |
| C | -3.05909 | 0.8764 | 3.22801 | H | 0.67701 | -0.48982 | 5.30833 |
| C | -1.77172 | 0.89735 | 3.62787 | H | 0.74431 | -2.24289 | 5.1619 |
| C | -4.20953 | 0.23044 | 1.08151 | H | -0.65887 | -1.37379 | 4.535 |
| C | -4.91661 | -0.97176 | 1.1094 | H | 3.49798 | 1.17356 | 2.6091 |
| C | -5.98553 | -1.1163 | 0.22644 | H | 3.25169 | 2.924 | 2.67186 |
| C | -6.33156 | -0.11057 | -0.67385 | H | 2.75481 | 2.00627 | 1.23825 |
| C | -5.5893 | 1.07124 | -0.67618 | H | 0.63266 | 1.78061 | 5.0375 |
| C | -4.51594 | 1.26209 | 0.18949 | H | 2.00815 | 2.84216 | 4.77007 |
| C | 1.14537 | -1.27349 | 3.29036 | H | 2.26628 | 1.10417 | 4.88682 |
| C | 0.6856 | -2.48099 | 2.45596 | H | 1.08468 | 3.94349 | 2.7923 |
| C | 2.66836 | -1.36253 | 3.4934 | H | -0.40269 | 3.0419 | 3.11506 |
| C | 0.42756 | -1.32671 | 4.65131 | H | 0.30496 | 3.02453 | 1.50044 |
| C | 1.42615 | 1.82873 | 2.97511 | C | -3.66821 | 2.50507 | 0.12841 |
| C | 2.8163 | 1.97943 | 2.32737 | H | -2.65438 | 2.2584 | -0.20391 |
| C | 1.58444 | 1.86685 | 4.50605 | H | -4.09011 | 3.23118 | -0.5713 |
| C | 0.54063 | 3.02055 | 2.56232 | H | -3.57784 | 2.98603 | 1.10896 |
| C | 1.7933 | 1.31289 | -1.18946 | C | -4.48082 | -2.10011 | 2.00662 |
| C | 3.0494 | 1.48776 | -1.76064 | H | -3.51858 | -2.49519 | 1.66246 |
| C | 3.61837 | 2.74002 | -1.98717 | H | -4.35412 | -1.77673 | 3.0454 |
| C | 2.91835 | 3.88654 | -1.65024 | H | -5.20881 | -2.91523 | 1.98802 |
| C | 1.64481 | 3.76439 | -1.10884 | C | -7.43992 | -0.31757 | -1.67333 |
| C | 1.11867 | 2.49972 | -0.91403 | H | -7.95963 | 0.61962 | -1.89523 |
| C | 2.10633 | -1.3977 | -0.88575 | H | -7.0275 | -0.70014 | -2.61394 |
| C | 1.88541 | -2.52254 | -1.67716 | H | -8.17668 | -1.04191 | -1.31298 |
| C | 2.71215 | -3.64411 | -1.66104 | | | | |

**Supplementary Table 12.** Computed Cartesian coordinates (x, y, z) for the optimized structure of **2b'B[1]**.

E1 = -3514.207895, $\nu_{imag}$ = 7.6$i$ cm$^{-1}$ , G1 = -3513.649833, E2 = -3515.109541

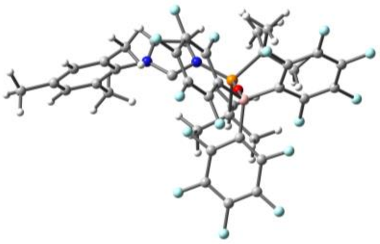

| | | | |
|---|---|---|---|
| P | 0.07669 | -2.12983 | -0.72666 |
| F | -1.01734 | 0.29875 | 1.81464 |
| F | -1.2108 | -0.04947 | 4.4398 |
| F | 1.02798 | 0.03653 | 6.01038 |
| F | 3.45441 | 0.47946 | 4.8506 |
| F | 3.66759 | 0.80076 | 2.23982 |
| F | 3.40962 | -1.42862 | 0.78843 |
| F | 5.73135 | -1.74243 | -0.43355 |
| F | 6.54635 | 0.03543 | -2.3392 |
| F | 4.93461 | 2.1366 | -2.98166 |
| F | 2.60163 | 2.46286 | -1.81519 |
| F | -0.2282 | 0.68735 | -2.31434 |
| F | -1.57633 | 2.78635 | -3.18059 |
| F | -1.66944 | 5.08394 | -1.72395 |
| F | -0.31882 | 5.22059 | 0.64581 |
| F | 1.071 | 3.14653 | 1.52773 |
| O | 0.70628 | -0.8366 | -0.25289 |
| N | -3.52823 | -1.1863 | -1.10933 |
| N | -1.50223 | -1.82411 | -1.33906 |
| C | -2.28912 | -0.94013 | -0.61226 |
| C | -3.54263 | -2.18679 | -2.08049 |
| C | -2.27624 | -2.61171 | -2.2192 |
| C | -4.71867 | -0.55608 | -0.61161 |
| C | -4.8979 | 0.81669 | -0.80012 |
| C | -6.07787 | 1.3924 | -0.32578 |
| C | -7.05937 | 0.64051 | 0.32057 |
| C | -6.84004 | -0.7211 | 0.49099 |
| C | -5.67774 | -1.34343 | 0.0354 |
| C | 1.13544 | -2.81247 | -2.12017 |
| C | 1.50218 | -1.62388 | -3.02699 |
| C | 2.42696 | -3.39512 | -1.50628 |
| C | 0.50961 | -3.90251 | -3.01048 |
| C | -0.16693 | -3.30931 | 0.6922 |
| C | 1.06914 | -3.27958 | 1.60906 |
| C | -0.42902 | -4.73598 | 0.17674 |
| C | -1.39352 | -2.8812 | 1.52048 |
| C | 1.34176 | 0.45554 | 1.86589 |
| C | 2.44242 | 0.54682 | 2.71416 |
| C | 2.35871 | 0.40286 | 4.0953 |
| C | 1.12224 | 0.18527 | 4.69152 |
| C | -0.01149 | 0.14171 | 3.89426 |
| C | 0.12341 | 0.30453 | 2.52233 |
| C | 2.84518 | 0.5148 | -0.45229 |
| C | 3.31602 | 1.4017 | -1.41989 |
| C | 4.54985 | 1.26057 | -2.05475 |
| C | 5.375 | 0.19578 | -1.72989 |
| C | 4.96075 | -0.70807 | -0.76114 |
| C | 3.73452 | -0.5136 | -0.1489 |
| C | 0.4466 | 1.77168 | -0.30786 |
| C | 0.39849 | 2.98458 | 0.37868 |
| C | -0.30263 | 4.09867 | -0.06877 |
| C | -0.98165 | 4.03501 | -1.27796 |
| C | -0.9319 | 2.86596 | -2.01466 |
| C | -0.23925 | 1.77174 | -1.51938 |
| B | 1.35547 | 0.53363 | 0.23176 |
| H | -4.44571 | -2.48071 | -2.59042 |
| H | -1.88277 | -3.37417 | -2.86919 |
| H | -6.22709 | 2.45987 | -0.46507 |
| H | -7.58825 | -1.32277 | 1.00307 |
| H | 0.6123 | -1.15627 | -3.46348 |
| H | 2.12322 | -2.00516 | -3.84409 |
| H | 2.0639 | -0.85552 | -2.49365 |
| H | 3.15297 | -3.52375 | -2.31663 |
| H | 2.26188 | -4.37386 | -1.05784 |
| H | 2.8768 | -2.74591 | -0.75766 |
| H | -0.007 | -4.6957 | -2.46479 |
| H | 1.33 | -4.37954 | -3.56702 |
| H | -0.16302 | -3.4777 | -3.7621 |
| H | 1.98068 | -3.61124 | 1.11575 |
| H | 0.88366 | -3.95695 | 2.45012 |
| H | 1.23994 | -2.2824 | 2.0101 |
| H | -1.27804 | -4.76969 | -0.51416 |
| H | -0.68134 | -5.36814 | 1.03449 |
| H | 0.44482 | -5.18094 | -0.309 |
| H | -1.41173 | -3.49765 | 2.42874 |
| H | -2.32581 | -3.06463 | 0.97357 |
| H | -1.37834 | -1.83019 | 1.80877 |
| C | -5.48088 | -2.82401 | 0.27396 |
| H | -4.4464 | -3.0532 | 0.55674 |
| H | -6.13451 | -3.17475 | 1.07712 |
| H | -5.71348 | -3.41731 | -0.6216 |
| C | -3.84206 | 1.67069 | -1.44951 |
| H | -3.01803 | 1.83848 | -0.75087 |
| H | -3.41588 | 1.19119 | -2.33823 |
| H | -4.2544 | 2.6396 | -1.74143 |
| C | -8.31178 | 1.30226 | 0.84552 |
| H | -9.10347 | 0.5694 | 1.02015 |
| H | -8.1095 | 1.80985 | 1.79708 |
| H | -8.68821 | 2.05423 | 0.14368 |

**Supplementary Table 13.** Computed Cartesian coordinates (x, y, z) for the optimized structure of **TS2b**.

E1 = -3514.226865, G1 = -3513.675420, E2 = -3515.123885

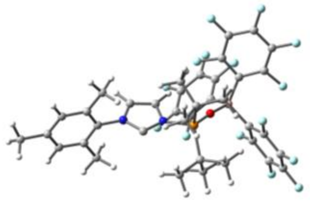

| | | | |
|---|---|---|---|
| P | 0.6622 | 0.32846 | -1.50732 |
| F | -0.35418 | 2.00205 | 1.64449 |
| F | -1.07227 | 4.53601 | 1.94657 |
| F | -3.56961 | 5.36345 | 1.19906 |
| F | -5.31064 | 3.56097 | 0.13683 |
| F | -4.6118 | 1.03605 | -0.20146 |
| F | -2.9185 | 0.55768 | -2.2388 |
| F | -4.23517 | -1.04029 | -3.89469 |
| F | -4.88966 | -3.58214 | -3.13228 |
| F | -4.1629 | -4.46293 | -0.65928 |
| F | -2.82519 | -2.91401 | 0.99289 |
| F | 0.2951 | -2.16927 | 0.66204 |
| F | 1.16358 | -3.21523 | 2.93632 |
| F | -0.00134 | -2.5239 | 5.30844 |
| F | -2.05501 | -0.73393 | 5.31666 |
| F | -2.91716 | 0.34727 | 3.06352 |
| O | -0.53252 | 0.17793 | -0.57462 |
| N | 4.1033 | 0.08564 | 0.00376 |
| N | 2.05307 | 0.08048 | -0.53989 |
| C | 3.31879 | 0.0606 | -1.09703 |
| C | 3.38099 | 0.15325 | 1.19591 |
| C | 2.07984 | 0.16641 | 0.85882 |
| C | 5.53484 | 0.03551 | -0.0442 |
| C | 6.24779 | 1.22066 | -0.23189 |
| C | 7.6405 | 1.14638 | -0.24631 |
| C | 8.31131 | -0.06445 | -0.08148 |
| C | 7.55914 | -1.2263 | 0.09951 |
| C | 6.16673 | -1.20063 | 0.11946 |
| C | 0.6303 | -0.94318 | -2.87163 |
| C | 0.214 | -2.28479 | -2.24316 |
| C | -0.42065 | -0.51787 | -3.91578 |
| C | 1.97546 | -1.15168 | -3.59171 |
| C | 0.79348 | 2.09448 | -2.07908 |
| C | -0.60303 | 2.61758 | -2.45546 |
| C | 1.76668 | 2.24001 | -3.26119 |
| C | 1.34779 | 2.9289 | -0.90682 |
| C | -2.40478 | 1.39692 | 0.61143 |
| C | -3.67984 | 1.85704 | 0.29606 |
| C | -4.08228 | 3.177 | 0.47957 |
| C | -3.20161 | 4.09756 | 1.0259 |
| C | -1.93348 | 3.67664 | 1.40124 |
| C | -1.58428 | 2.34994 | 1.20793 |
| C | -2.75767 | -1.08957 | -0.54467 |
| C | -3.13254 | -2.3861 | -0.19749 |
| C | -3.84619 | -3.22919 | -1.04785 |

| | | | |
|---|---|---|---|
| C | -4.22103 | -2.78549 | -2.30511 |
| C | -3.89152 | -1.4919 | -2.68832 |
| C | -3.19382 | -0.68706 | -1.80484 |
| C | -1.29992 | -0.78742 | 1.73786 |
| C | -1.88333 | -0.49959 | 2.97065 |
| C | -1.46309 | -1.06453 | 4.17014 |
| C | -0.42185 | -1.97978 | 4.17035 |
| C | 0.17022 | -2.32588 | 2.96472 |
| C | -0.29359 | -1.74588 | 1.79327 |
| B | -1.80738 | -0.10232 | 0.34168 |
| H | 3.86435 | 0.18323 | 2.15984 |
| H | 1.18832 | 0.24778 | 1.45573 |
| H | 8.21354 | 2.05977 | -0.38894 |
| H | 8.06994 | -2.17822 | 0.22706 |
| H | 0.94749 | -2.63034 | -1.51205 |
| H | 0.16152 | -3.02758 | -3.04682 |
| H | -0.76279 | -2.23436 | -1.76002 |
| H | -0.58016 | -1.36143 | -4.59617 |
| H | -0.07783 | 0.32525 | -4.52114 |
| H | -1.38382 | -0.25757 | -3.48096 |
| H | 2.35389 | -0.24432 | -4.06399 |
| H | 1.80313 | -1.89601 | -4.37846 |
| H | 2.75081 | -1.51606 | -2.91892 |
| H | -1.03935 | 2.08486 | -3.30289 |
| H | -0.50594 | 3.6716 | -2.73874 |
| H | -1.29993 | 2.552 | -1.61668 |
| H | 2.73833 | 1.78834 | -3.03678 |
| H | 1.91869 | 3.30983 | -3.44423 |
| H | 1.37587 | 1.80432 | -4.18435 |
| H | 1.28706 | 3.98649 | -1.1864 |
| H | 2.39784 | 2.69373 | -0.70918 |
| H | 0.78475 | 2.79473 | 0.01676 |
| C | 5.35794 | -2.45943 | 0.2975 |
| H | 4.64301 | -2.58174 | -0.52331 |
| H | 6.0065 | -3.33891 | 0.3248 |
| H | 4.77597 | -2.43865 | 1.22604 |
| C | 5.52951 | 2.52992 | -0.4304 |
| H | 4.96151 | 2.51871 | -1.3675 |
| H | 4.81472 | 2.72362 | 0.37741 |
| H | 6.23734 | 3.36216 | -0.46688 |
| C | 9.81753 | -0.12931 | -0.12259 |
| H | 10.20608 | -0.8134 | 0.63877 |
| H | 10.16545 | -0.49291 | -1.09684 |
| H | 10.26611 | 0.85453 | 0.04348 |

**Supplementary Table 14.** Computed Cartesian coordinates (x, y, z) for the optimized structure of **2bB[1]**.

E1 = -1503.093802, G1 = -1502.528122, E2 = -1503.426000

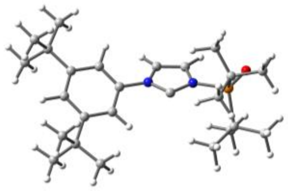

| | | | |
|---|---|---|---|
| P | 3.74122700 | -0.56269000 | 0.13208700 |
| O | 4.55042600 | -1.71917600 | 0.62572700 |
| N | -0.05546200 | -0.80930700 | 0.55087100 |
| N | 2.06715800 | -0.87683800 | 0.49073400 |
| C | 1.00976600 | -0.12612500 | 0.04622700 |
| C | 0.32334800 | -1.94040700 | 1.27734500 |
| C | 1.66871600 | -1.98746000 | 1.23649500 |
| C | -1.40437900 | -0.40433500 | 0.33508200 |
| C | -2.41192800 | -1.35945000 | 0.25755300 |
| C | -3.73593500 | -0.96687900 | 0.05408900 |
| C | -4.00021400 | 0.39732200 | -0.07946700 |
| C | -2.99765500 | 1.37005300 | -0.02198300 |
| C | -1.68750600 | 0.94995900 | 0.19688900 |
| C | 4.12240600 | 0.98830700 | 1.08534100 |
| C | 3.99717500 | 0.57278300 | 2.56531700 |
| C | 5.58072600 | 1.37797000 | 0.78988000 |
| C | 3.19548900 | 2.18362100 | 0.82398900 |
| C | 3.81591100 | -0.41459600 | -1.71850500 |
| C | 5.29031800 | -0.59827900 | -2.12360500 |
| C | 3.26640100 | 0.90432500 | -2.27612300 |
| C | 3.00022500 | -1.59381900 | -2.28404700 |
| H | -0.38659400 | -2.58017500 | 1.77646200 |
| H | 2.38397100 | -2.68024900 | 1.65030000 |
| H | 2.96312900 | 0.31510800 | 2.81915200 |
| H | 4.29742700 | 1.41730400 | 3.19700900 |
| H | 4.63688300 | -0.28258500 | 2.79824700 |
| H | 5.89140900 | 2.16183600 | 1.49103800 |
| H | 5.69330200 | 1.78187600 | -0.22195000 |
| H | 6.25976100 | 0.52744500 | 0.90738800 |
| H | 3.34172500 | 2.61555700 | -0.16837800 |
| H | 3.42302500 | 2.96403700 | 1.56088000 |
| H | 2.14133600 | 1.91124900 | 0.91648000 |
| H | 5.92427700 | 0.21893500 | -1.76641000 |
| H | 5.35611300 | -0.61300800 | -3.21821200 |
| H | 5.69390300 | -1.53727100 | -1.73557400 |
| H | 2.25982400 | 1.10511200 | -1.89705300 |
| H | 3.22030800 | 0.83100900 | -3.36986000 |
| H | 3.91996700 | 1.74859600 | -2.03335200 |
| H | 3.17811200 | -1.65808400 | -3.36412500 |
| H | 1.92831300 | -1.45053600 | -2.12322000 |
| H | 3.30257000 | -2.54481700 | -1.83324700 |
| H | -5.02618800 | 0.71524500 | -0.24225000 |
| H | -2.15241800 | -2.40885700 | 0.32864400 |
| H | -0.86068300 | 1.64597000 | 0.25022300 |
| C | -4.88533600 | -1.97848200 | -0.04689800 |
| C | -5.55812600 | -1.84729300 | -1.42678400 |
| C | -4.40300200 | -3.42689200 | 0.11516600 |
| C | -5.91816000 | -1.69049900 | 1.05962100 |
| H | -5.98102400 | -0.84905500 | -1.57945300 |
| H | -4.83629700 | -2.03324900 | -2.22946400 |
| H | -6.37334200 | -2.57432400 | -1.52227800 |
| H | -3.93139200 | -3.59069300 | 1.09086200 |
| H | -5.25677000 | -4.10937000 | 0.04336600 |
| H | -3.68915500 | -3.70855500 | -0.66693000 |
| H | -6.74277400 | -2.41098700 | 1.00519100 |
| H | -5.45825500 | -1.77035600 | 2.05067700 |
| H | -6.34486900 | -0.68658900 | 0.96637200 |
| C | -3.36248800 | 2.84976800 | -0.19654000 |
| C | -2.13081200 | 3.76323700 | -0.12202900 |
| C | -4.02568800 | 3.04876100 | -1.57307300 |
| C | -4.34306300 | 3.26963300 | 0.91479500 |
| H | -1.63176600 | 3.69191800 | 0.85069500 |
| H | -1.40011800 | 3.52391400 | -0.90225500 |
| H | -2.43785600 | 4.80554600 | -0.26152900 |
| H | -4.94958900 | 2.46896600 | -1.66783800 |
| H | -4.27705200 | 4.10522700 | -1.72397000 |
| H | -3.34936800 | 2.73911300 | -2.37713200 |
| H | -4.61066700 | 4.32711500 | 0.80419700 |
| H | -5.26866300 | 2.68541300 | 0.88326000 |
| H | -3.89132900 | 3.13171100 | 1.90326600 |

**Supplementary Table 15.** Computed Cartesian coordinates (x, y, z) for the optimized structure of **1c**.

E1 = -3710.769372, G1 = -3710.067587, E2 = -3711.720449

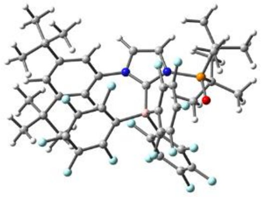

| | | | |
|---|---|---|---|
| P | -3.41292 | -0.4798 | -1.52976 |
| F | 0.0268 | 2.57353 | -0.81392 |
| F | -1.0869 | 4.9426 | -0.49017 |
| F | -3.22586 | 5.24295 | 1.20103 |
| F | -4.16733 | 3.06932 | 2.54082 |
| F | -3.04457 | 0.73083 | 2.29712 |
| F | -0.97478 | -2.70441 | -0.36906 |
| F | -1.80604 | -4.73353 | 1.12867 |
| F | -2.17595 | -4.40587 | 3.81957 |
| F | -1.67294 | -1.97664 | 4.9505 |
| F | -0.86218 | 0.07112 | 3.44212 |
| F | 1.45055 | -2.04979 | 0.62047 |
| F | 3.86539 | -2.06941 | 1.65657 |
| F | 4.95014 | 0.21379 | 2.70174 |
| F | 3.45852 | 2.49245 | 2.73548 |
| F | 1.03646 | 2.5454 | 1.7836 |
| O | -3.65704 | -0.33703 | -0.07457 |
| N | 0.50674 | -0.21055 | -1.93437 |
| N | -1.64814 | -0.39944 | -1.89347 |
| C | -0.54949 | -0.19917 | -1.08499 |
| C | 0.09862 | -0.41034 | -3.23936 |
| C | -1.23753 | -0.52512 | -3.21442 |
| C | 1.93245 | -0.11672 | -1.70016 |
| C | 2.66844 | -1.28565 | -1.81106 |
| C | 4.05587 | -1.24682 | -1.65635 |
| C | 4.64716 | -0.00148 | -1.43491 |
| C | 3.91552 | 1.18716 | -1.33746 |
| C | 2.53221 | 1.11512 | -1.48922 |
| C | -4.05353 | -2.13993 | -2.15581 |
| C | -3.8578 | -3.16343 | -1.02125 |
| C | -5.57544 | -1.98095 | -2.35679 |
| C | -3.42847 | -2.70566 | -3.43728 |
| C | -4.08968 | 0.98211 | -2.49645 |
| C | -5.50585 | 1.25779 | -1.94139 |
| C | -4.14835 | 0.83787 | -4.02534 |
| C | -3.23326 | 2.20533 | -2.13921 |
| C | -1.36999 | 1.50706 | 0.7736 |
| C | -2.4846 | 1.70918 | 1.59167 |
| C | -3.1104 | 2.94657 | 1.73699 |
| C | -2.63689 | 4.05803 | 1.05858 |
| C | -1.54944 | 3.90605 | 0.21347 |
| C | -0.97895 | 2.65133 | 0.08464 |
| C | -1.05724 | -1.1677 | 1.43638 |
| C | -1.16705 | -1.0771 | 2.82203 |
| C | -1.55486 | -2.13498 | 3.63211 |
| C | -1.80236 | -3.37651 | 3.06163 |
| C | -1.62437 | -3.53672 | 1.69691 |
| C | -1.23587 | -2.44559 | 0.93325 |
| C | 1.06019 | 0.2416 | 1.12922 |
| C | 1.65498 | 1.35867 | 1.71378 |
| C | 2.94106 | 1.36313 | 2.24495 |
| C | 3.70565 | 0.21145 | 2.23024 |
| C | 3.15509 | -0.9372 | 1.68856 |
| C | 1.87315 | -0.89571 | 1.16584 |
| B | -0.50536 | 0.10849 | 0.56805 |
| H | 0.81021 | -0.44425 | -4.04757 |
| H | -1.9261 | -0.68958 | -4.0216 |
| H | -2.82272 | -3.48551 | -0.94272 |
| H | -4.46566 | -4.04678 | -1.2481 |
| H | -4.17614 | -2.76039 | -0.05694 |
| H | -5.99597 | -2.97426 | -2.54858 |
| H | -5.83609 | -1.35013 | -3.21002 |
| H | -6.06176 | -1.58664 | -1.45958 |
| H | -3.58894 | -2.07041 | -4.31496 |
| H | -3.90608 | -3.66921 | -3.6488 |
| H | -2.35758 | -2.89283 | -3.31906 |
| H | -6.25261 | 0.55056 | -2.30521 |
| H | -5.81173 | 2.25707 | -2.27178 |
| H | -5.51089 | 1.24394 | -0.84826 |
| H | -3.159 | 0.90782 | -4.48587 |
| H | -4.73681 | 1.67247 | -4.42342 |
| H | -4.63021 | -0.08382 | -4.36187 |
| H | -3.62477 | 3.07721 | -2.67554 |
| H | -2.17982 | 2.09138 | -2.41046 |
| H | -3.30196 | 2.41146 | -1.07111 |
| H | 5.72424 | 0.04785 | -1.32587 |
| H | 2.14104 | -2.21947 | -1.96045 |
| H | 1.91488 | 1.99554 | -1.41321 |
| C | 4.9015 | -2.52232 | -1.75877 |
| C | 6.12037 | -2.45587 | -0.82043 |
| C | 4.07619 | -3.76395 | -1.37827 |
| C | 5.39146 | -2.66365 | -3.21247 |
| H | 6.83947 | -1.68922 | -1.12628 |
| H | 5.8128 | -2.25185 | 0.20893 |
| H | 6.64999 | -3.41451 | -0.83821 |
| H | 3.29318 | -3.9799 | -2.11315 |
| H | 4.72777 | -4.64333 | -1.33961 |
| H | 3.60625 | -3.63791 | -0.39847 |
| H | 5.99574 | -3.57149 | -3.32588 |
| H | 4.54656 | -2.72731 | -3.90779 |
| H | 6.00582 | -1.8047 | -3.50555 |
| C | 4.63092 | 2.52891 | -1.13097 |
| C | 3.65183 | 3.65097 | -0.75093 |
| C | 5.68959 | 2.42654 | -0.01738 |
| C | 5.32677 | 2.9034 | -2.45451 |
| H | 2.93486 | 3.86499 | -1.55086 |
| H | 3.08992 | 3.41521 | 0.15865 |
| H | 4.2107 | 4.57416 | -0.56474 |
| H | 6.46314 | 1.68538 | -0.24056 |
| H | 6.19125 | 3.39359 | 0.09968 |
| H | 5.23946 | 2.16802 | 0.94393 |
| H | 5.84546 | 3.86391 | -2.35399 |
| H | 6.06465 | 2.14674 | -2.74349 |
| H | 4.59678 | 2.99153 | -3.26697 |

**Supplementary Table 16.** Computed Cartesian coordinates (x, y, z) for the optimized structure of **3cB**[1].

E1 = -3710.717019, $\nu_{imag}$ = 29.9$i$ cm$^{-1}$ , G1 = -3710.021538, E2 = -3711.661928

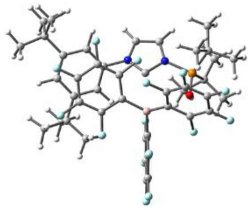

| | | | |
|---|---|---|---|
| P | 2.51398 | -1.92074 | 1.63123 |
| F | 0.23833 | 1.18248 | 2.08771 |
| F | 0.36555 | 3.40808 | 3.46763 |
| F | 1.18308 | 5.73778 | 2.30768 |
| F | 1.86834 | 5.75321 | -0.33289 |
| F | 1.73182 | 3.49329 | -1.7661 |
| F | 1.17628 | -0.29654 | -3.60674 |
| F | 3.4333 | -1.08831 | -4.7043 |
| F | 5.8113 | -0.61985 | -3.46867 |
| F | 5.86731 | 0.71165 | -1.10141 |
| F | 3.64165 | 1.59709 | -0.01448 |
| F | 0.0025 | -1.70469 | -1.77148 |
| F | -2.42925 | -2.2929 | -2.63231 |
| F | -4.29117 | -0.33036 | -2.96796 |
| F | -3.64893 | 2.24656 | -2.38904 |
| F | -1.25752 | 2.86008 | -1.44628 |
| O | 2.75456 | -0.57683 | 1.03808 |
| N | -1.25037 | -1.98543 | 1.21083 |
| N | 0.82901 | -2.33148 | 1.49902 |
| C | -0.04058 | -1.40348 | 0.97507 |
| C | -1.14879 | -3.21637 | 1.85268 |
| C | 0.16472 | -3.43707 | 2.0427 |
| C | -2.50498 | -1.36106 | 0.93777 |
| C | -3.54367 | -2.10736 | 0.40067 |
| C | -4.78693 | -1.51022 | 0.17089 |
| C | -4.93812 | -0.16533 | 0.51412 |
| C | -3.90114 | 0.59817 | 1.06658 |
| C | -2.66952 | -0.02056 | 1.26476 |
| C | 3.39912 | -3.24485 | 0.67664 |
| C | 3.06084 | -2.96045 | -0.79809 |
| C | 4.91121 | -3.05022 | 0.88642 |
| C | 2.99807 | -4.69056 | 1.00243 |
| C | 2.85377 | -1.88727 | 3.46374 |
| C | 4.12439 | -1.03428 | 3.65132 |
| C | 3.05419 | -3.2524 | 4.13411 |
| C | 1.67088 | -1.15279 | 4.12408 |
| C | 0.99375 | 2.22398 | 0.08015 |
| C | 1.40699 | 3.43976 | -0.46547 |
| C | 1.47836 | 4.62196 | 0.25097 |
| C | 1.129 | 4.61688 | 1.59581 |
| C | 0.71901 | 3.43145 | 2.1819 |
| C | 0.65608 | 2.26565 | 1.42522 |
| C | 2.24959 | 0.5776 | -1.66212 |
| C | 3.51114 | 0.87081 | -1.11499 |
| C | 4.70113 | 0.44983 | -1.69044 |
| C | 4.67845 | -0.22935 | -2.90041 |
| C | 3.46366 | -0.47473 | -3.52233 |
| C | 2.28807 | -0.06545 | -2.90736 |
| C | -0.50386 | 0.60079 | -1.50354 |
| C | -1.49187 | 1.5655 | -1.70184 |
| C | -2.7557 | 1.27504 | -2.19897 |
| C | -3.08141 | -0.03421 | -2.50279 |
| C | -2.12591 | -1.02832 | -2.34066 |
| C | -0.86603 | -0.69743 | -1.87233 |
| B | 0.91888 | 0.99656 | -0.93391 |
| H | -2.01233 | -3.79818 | 2.13519 |
| H | 0.6675 | -4.27013 | 2.50489 |
| H | 1.98629 | -3.02804 | -0.98859 |
| H | 3.57035 | -3.69785 | -1.42974 |
| H | 3.39632 | -1.96403 | -1.08737 |
| H | 5.45544 | -3.6829 | 0.17542 |
| H | 5.2307 | -3.33946 | 1.89308 |
| H | 5.20906 | -2.01151 | 0.70902 |
| H | 3.14518 | -4.95349 | 2.05355 |
| H | 3.62379 | -5.3655 | 0.40645 |
| H | 1.95771 | -4.88987 | 0.72982 |
| H | 5.01238 | -1.51193 | 3.22671 |
| H | 4.30114 | -0.89624 | 4.72462 |
| H | 4.00966 | -0.05321 | 3.18479 |
| H | 2.1816 | -3.90557 | 4.03711 |
| H | 3.21733 | -3.09409 | 5.20682 |
| H | 3.93139 | -3.77932 | 3.74674 |
| H | 1.92267 | -0.95176 | 5.17205 |
| H | 0.75456 | -1.75148 | 4.10718 |
| H | 1.46531 | -0.19856 | 3.63227 |
| H | -5.89826 | 0.30769 | 0.35567 |
| H | -3.36749 | -3.1456 | 0.13856 |
| H | -1.82151 | 0.50237 | 1.68424 |
| C | -5.94419 | -2.35026 | -0.38589 |
| C | -7.12415 | -1.47722 | -0.83795 |
| C | -5.48355 | -3.18388 | -1.59612 |
| C | -6.42823 | -3.29851 | 0.72849 |
| H | -7.58791 | -0.94502 | -0.0003 |
| H | -6.80987 | -0.74305 | -1.58737 |
| H | -7.89707 | -2.10988 | -1.28736 |
| H | -4.69989 | -3.901 | -1.33348 |
| H | -6.32892 | -3.75713 | -1.99335 |
| H | -5.09772 | -2.54109 | -2.39049 |
| H | -7.26363 | -3.91328 | 0.37264 |
| H | -5.6266 | -3.97226 | 1.05135 |
| H | -6.7664 | -2.73277 | 1.60379 |
| C | -4.14615 | 2.03403 | 1.55132 |
| C | -2.84093 | 2.84555 | 1.60948 |
| C | -5.12891 | 2.78235 | 0.63439 |
| C | -4.74443 | 1.94971 | 2.96949 |
| H | -2.14299 | 2.46446 | 2.36126 |
| H | -2.33795 | 2.85025 | 0.63861 |
| H | -3.06406 | 3.88387 | 1.87762 |
| H | -6.13287 | 2.34657 | 0.65579 |
| H | -5.22554 | 3.82134 | 0.96813 |
| H | -4.77869 | 2.7872 | -0.4011 |
| H | -4.92889 | 2.95486 | 3.36727 |
| H | -5.6945 | 1.40375 | 2.96368 |
| H | -4.05991 | 1.43211 | 3.65054 |

**Supplementary Table 17.** Computed Cartesian coordinates (x, y, z) for the optimized structure of **TS1c**.

E1 = -3710.735116, G1 = -3710.031185, E2 = -3711.686283

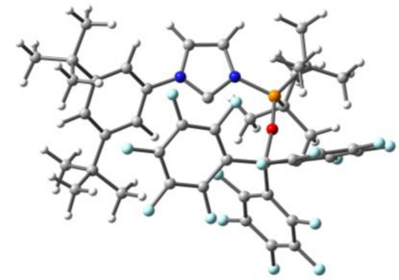

| | | | |
|---|---|---|---|
| P | 1.49137 | -1.0437 | 2.1779 |
| F | 0.05717 | 2.1523 | 0.77125 |
| F | 0.72842 | 4.61114 | 1.49527 |
| F | 3.08398 | 5.70712 | 0.64031 |
| F | 4.73465 | 4.24243 | -0.95601 |
| F | 4.10607 | 1.78431 | -1.66457 |
| F | 1.45055 | -1.33914 | -3.28881 |
| F | 3.28312 | -2.99203 | -4.21255 |
| F | 5.67027 | -3.29349 | -2.9359 |
| F | 6.17872 | -1.83465 | -0.68045 |
| F | 4.36911 | -0.13332 | 0.25032 |
| F | -0.01702 | -2.18145 | -0.67747 |
| F | -2.52411 | -2.39731 | -1.52004 |
| F | -3.73924 | -0.29063 | -2.77755 |
| F | -2.35242 | 2.03476 | -3.12072 |
| F | 0.09833 | 2.29597 | -2.21418 |
| O | 1.66986 | -0.40077 | 0.82158 |
| N | -2.2464 | -0.98263 | 2.05981 |
| N | -0.18229 | -1.32603 | 2.43952 |
| C | -1.02702 | -0.65306 | 1.58407 |
| C | -2.19408 | -1.79697 | 3.18534 |
| C | -0.88768 | -2.0079 | 3.44768 |
| C | -3.42151 | -0.54152 | 1.37682 |
| C | -4.40501 | -1.4608 | 1.04198 |
| C | -5.50189 | -1.04913 | 0.28563 |
| C | -5.57184 | 0.29654 | -0.08496 |
| C | -4.58418 | 1.23084 | 0.23881 |
| C | -3.49086 | 0.78686 | 0.98164 |
| C | 2.36277 | -2.68897 | 2.23656 |
| C | 2.07294 | -3.41497 | 0.9107 |
| C | 3.88196 | -2.46681 | 2.3679 |
| C | 1.88868 | -3.6043 | 3.38074 |
| C | 1.98967 | 0.15058 | 3.5102 |
| C | 3.27203 | 0.87936 | 3.06959 |
| C | 2.20407 | -0.53974 | 4.86731 |
| C | 0.85385 | 1.17919 | 3.68056 |
| C | 2.08152 | 1.78708 | -0.40686 |
| C | 3.24385 | 2.40942 | -0.85306 |
| C | 3.59555 | 3.71299 | -0.51031 |
| C | 2.75785 | 4.46385 | 0.29749 |
| C | 1.56514 | 3.90332 | 0.73397 |
| C | 1.25293 | 2.60718 | 0.35822 |
| C | 2.7834 | -0.6793 | -1.42613 |
| C | 4.04132 | -0.84151 | -0.84855 |
| C | 5.01277 | -1.70838 | -1.31719 |
| C | 4.75584 | -2.45202 | -2.46219 |
| C | 3.53923 | -2.29718 | -3.10506 |
| C | 2.58569 | -1.42159 | -2.58711 |
| C | 0.16229 | 0.08785 | -1.31608 |
| C | -0.4919 | 1.11142 | -1.99215 |
| C | -1.78644 | 0.99917 | -2.49035 |
| C | -2.49251 | -0.17766 | -2.32196 |
| C | -1.87037 | -1.24257 | -1.68798 |
| C | -0.57236 | -1.09023 | -1.23113 |
| B | 1.65493 | 0.22938 | -0.6707 |
| H | -3.07788 | -2.13637 | 3.70352 |
| H | -0.42342 | -2.55931 | 4.24761 |
| H | 1.00684 | -3.61025 | 0.78181 |
| H | 2.59744 | -4.37698 | 0.93315 |
| H | 2.42404 | -2.851 | 0.04568 |
| H | 4.3795 | -3.43126 | 2.21781 |
| H | 4.16644 | -2.10571 | 3.36018 |
| H | 4.27293 | -1.77359 | 1.62352 |
| H | 1.94524 | -3.14179 | 4.36924 |
| H | 2.54641 | -4.48052 | 3.39715 |
| H | 0.87318 | -3.96944 | 3.20974 |
| H | 4.12333 | 0.20444 | 2.95401 |
| H | 3.53019 | 1.61744 | 3.83704 |
| H | 3.12522 | 1.40417 | 2.12301 |
| H | 1.30753 | -1.07094 | 5.20549 |
| H | 2.41605 | 0.23506 | 5.61243 |
| H | 3.04881 | -1.23279 | 4.87012 |
| H | 1.20705 | 1.96346 | 4.3594 |
| H | -0.03761 | 0.72534 | 4.12434 |
| H | 0.56297 | 1.64588 | 2.74052 |
| H | -6.42017 | 0.62438 | -0.67596 |
| H | -4.27024 | -2.49899 | 1.32281 |
| H | -2.66247 | 1.43745 | 1.23131 |
| C | -6.55928 | -2.03554 | -0.22253 |
| C | -6.42642 | -2.13062 | -1.75551 |
| C | -6.3707 | -3.44106 | 0.36559 |
| C | -7.97004 | -1.54485 | 0.15112 |
| H | -6.61598 | -1.16483 | -2.2348 |
| H | -5.41545 | -2.44418 | -2.03389 |
| H | -7.14387 | -2.85685 | -2.15609 |
| H | -6.42079 | -3.43275 | 1.46083 |
| H | -7.16552 | -4.10104 | 0.00176 |
| H | -5.41542 | -3.88336 | 0.06229 |
| H | -8.72325 | -2.25436 | -0.21064 |
| H | -8.07956 | -1.45398 | 1.23759 |
| H | -8.19631 | -0.57037 | -0.29265 |
| C | -4.7368 | 2.70143 | -0.17033 |
| C | -3.40574 | 3.4653 | -0.07944 |
| C | -5.25518 | 2.81898 | -1.6156 |
| C | -5.75194 | 3.35411 | 0.78832 |
| H | -3.02512 | 3.51859 | 0.94593 |
| H | -2.63356 | 3.0123 | -0.70874 |
| H | -3.553 | 4.49496 | -0.42272 |
| H | -6.26782 | 2.41862 | -1.72864 |
| H | -5.29196 | 3.87406 | -1.9089 |
| H | -4.59916 | 2.29293 | -2.31288 |
| H | -5.89503 | 4.41123 | 0.53442 |
| H | -6.7255 | 2.85455 | 0.73006 |
| H | -5.40329 | 3.29455 | 1.82551 |

**Supplementary Table 18.** Computed Cartesian coordinates (x, y, z) for the optimized structure of **2c'B¹**.

E1 = -3710.723658, $\nu_{imag}$ = 15.15$i$ cm$^{-1}$, G1 = -3710.031185, E2 = -3711.672561

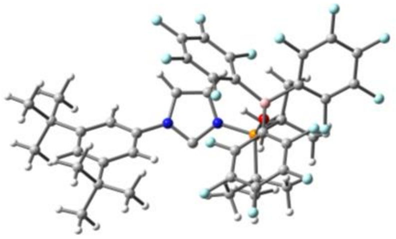

| | | | |
|---|---|---|---|
| P | 0.89051 | -0.5853 | 2.18786 |
| F | -0.35474 | 1.59613 | -0.44808 |
| F | -0.69986 | 4.22423 | -0.29975 |
| F | 1.45263 | 5.90608 | -0.39952 |
| F | 3.95508 | 4.86163 | -0.64604 |
| F | 4.3301 | 2.25505 | -0.78181 |
| F | 3.45775 | -1.32235 | -2.83884 |
| F | 5.87152 | -2.39971 | -2.77907 |
| F | 7.46702 | -2.13101 | -0.58816 |
| F | 6.56655 | -0.69954 | 1.55958 |
| F | 4.17614 | 0.44147 | 1.50176 |
| F | 1.56882 | -2.69033 | -0.58557 |
| F | -0.26609 | -3.9739 | -1.9954 |
| F | -1.63867 | -2.67375 | -3.98005 |
| F | -1.12384 | -0.03802 | -4.44386 |
| F | 0.63538 | 1.27743 | -2.98692 |
| O | 1.64971 | -0.26718 | 0.9076 |
| N | -2.64705 | -1.2379 | 1.1021 |
| N | -0.6252 | -1.28525 | 1.78092 |
| C | -1.75886 | -0.49708 | 1.80091 |
| C | -2.11662 | -2.44665 | 0.65768 |
| C | -0.84161 | -2.48799 | 1.07878 |
| C | -3.92292 | -0.72022 | 0.72329 |
| C | -5.03708 | -1.54757 | 0.68138 |
| C | -6.26894 | -1.0257 | 0.27926 |
| C | -6.32756 | 0.32991 | -0.05439 |
| C | -5.21307 | 1.17337 | -0.01473 |
| C | -3.99558 | 0.62233 | 0.37349 |
| C | 1.81226 | -1.93253 | 3.1266 |
| C | 2.44254 | -2.92681 | 2.13161 |
| C | 2.98729 | -1.36533 | 3.94986 |
| C | 0.8478 | -2.69487 | 4.05516 |
| C | 0.62115 | 0.95887 | 3.22392 |
| C | 1.97617 | 1.70355 | 3.23471 |
| C | 0.162 | 0.59145 | 4.64619 |
| C | -0.41743 | 1.9618 | 2.67505 |
| C | 2.00506 | 1.7703 | -0.58454 |
| C | 3.06513 | 2.67042 | -0.64833 |
| C | 2.89788 | 4.05278 | -0.58917 |
| C | 1.62675 | 4.58994 | -0.46803 |
| C | 0.53476 | 3.73334 | -0.42265 |
| C | 0.74982 | 2.36803 | -0.49073 |
| C | 3.64766 | -0.4425 | -0.63631 |
| C | 4.52865 | -0.29778 | 0.43274 |
| C | 5.79375 | -0.85998 | 0.48388 |
| C | 6.25467 | -1.58458 | -0.60698 |
| C | 5.43786 | -1.72057 | -1.71794 |
| C | 4.16725 | -1.15054 | -1.7161 |
| C | 1.11547 | -0.60604 | -1.60882 |
| C | 0.43571 | -0.00748 | -2.66821 |
| C | -0.48471 | -0.67926 | -3.46603 |
| C | -0.74274 | -2.02317 | -3.24219 |
| C | -0.04372 | -2.67888 | -2.24197 |
| C | 0.87757 | -1.96892 | -1.48931 |
| B | 2.12503 | 0.14313 | -0.55595 |
| H | -2.67589 | -3.14814 | 0.05851 |
| H | -0.08418 | -3.22675 | 0.89281 |
| H | 1.70962 | -3.54831 | 1.61921 |
| H | 3.08981 | -3.60021 | 2.70401 |
| H | 3.05218 | -2.42353 | 1.37925 |
| H | 3.49368 | -2.21164 | 4.42683 |
| H | 2.67944 | -0.68791 | 4.74753 |
| H | 3.71696 | -0.85993 | 3.31378 |
| H | 0.38998 | -2.05539 | 4.81257 |
| H | 1.42015 | -3.46919 | 4.57839 |
| H | 0.04609 | -3.19052 | 3.50157 |
| H | 2.822 | 1.10947 | 3.57499 |
| H | 1.87616 | 2.55557 | 3.9155 |
| H | 2.21188 | 2.08805 | 2.23911 |
| H | -0.77585 | 0.02749 | 4.61956 |
| H | -0.0325 | 1.52666 | 5.18257 |
| H | 0.90183 | 0.0353 | 5.22435 |
| H | -0.21568 | 2.92377 | 3.16165 |
| H | -1.43297 | 1.64182 | 2.89026 |
| H | -0.34868 | 2.10277 | 1.60254 |
| H | -7.28186 | 0.74813 | -0.36134 |
| H | -4.93704 | -2.58571 | 0.97807 |
| H | -3.08471 | 1.20576 | 0.40633 |
| C | -7.54289 | -1.8788 | 0.21491 |
| C | -8.12724 | -1.8209 | -1.20972 |
| C | -7.27691 | -3.35068 | 0.56063 |
| C | -8.57207 | -1.32363 | 1.21817 |
| H | -8.39577 | -0.8003 | -1.50012 |
| H | -7.40645 | -2.1996 | -1.94252 |
| H | -9.03437 | -2.43337 | -1.2716 |
| H | -6.89978 | -3.46905 | 1.58271 |
| H | -8.21068 | -3.91831 | 0.48716 |
| H | -6.55787 | -3.80514 | -0.13062 |
| H | -9.49579 | -1.91317 | 1.18257 |
| H | -8.17815 | -1.36331 | 2.23965 |
| H | -8.82939 | -0.2824 | 0.99841 |
| C | -5.34711 | 2.6508 | -0.40213 |
| C | -4.0126 | 3.40193 | -0.28775 |
| C | -5.83871 | 2.75358 | -1.85839 |
| C | -6.36669 | 3.32437 | 0.53619 |
| H | -3.61849 | 3.3818 | 0.73439 |
| H | -3.2476 | 2.99051 | -0.95434 |
| H | -4.15875 | 4.45189 | -0.56324 |
| H | -6.81061 | 2.26773 | -1.99614 |
| H | -5.94724 | 3.80552 | -2.14683 |
| H | -5.12602 | 2.28262 | -2.54397 |
| H | -6.47238 | 4.38579 | 0.28361 |
| H | -7.35688 | 2.86287 | 0.45863 |
| H | -6.04186 | 3.2511 | 1.57986 |

**Supplementary Table 19.** Computed Cartesian coordinates (x, y, z) for the optimized structure of **TS2c**.

E1 = -3710.736615, G1 = -3710.045490, E2 = -3711.684290

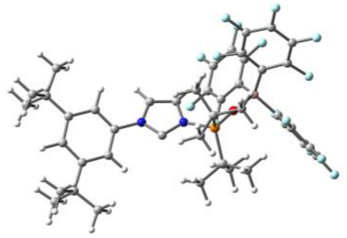

| | | | | | | | |
|---|---|---|---|---|---|---|---|
| P | 0.11257 | 1.21079 | -0.85211 | C | 0.89486 | -1.70526 | 3.46869 |
| F | 1.09931 | -2.33434 | -1.0436 | C | 1.27815 | -0.89241 | 2.41183 |
| F | 1.66327 | -3.67317 | -3.26396 | B | 2.64062 | -0.27097 | 0.20883 |
| F | 4.0752 | -3.3344 | -4.50632 | H | -3.00738 | -2.00947 | 1.05734 |
| F | 5.89334 | -1.60838 | -3.446 | H | -0.36213 | -1.37947 | 0.64633 |
| F | 5.34896 | -0.24516 | -1.24827 | H | -0.06213 | 2.44404 | 1.84389 |
| F | 3.85982 | 0.30268 | 2.96112 | H | 0.68735 | 4.01626 | 1.52939 |
| F | 5.24164 | 2.45026 | 3.58828 | H | 1.62148 | 2.52765 | 1.29789 |
| F | 5.83282 | 4.33705 | 1.71596 | H | 1.30249 | 4.75027 | -0.65819 |
| F | 4.99367 | 3.97667 | -0.86074 | H | 0.75726 | 3.96561 | -2.13914 |
| F | 3.62719 | 1.80621 | -1.53158 | H | 2.11581 | 3.29332 | -1.22805 |
| F | 0.6824 | 0.31085 | 2.35774 | H | -1.66113 | 3.7177 | -1.31027 |
| F | -0.03383 | -1.30167 | 4.33575 | H | -1.04567 | 4.73164 | 0.00331 |
| F | 1.15673 | -3.74096 | 4.63007 | H | -1.94118 | 3.23863 | 0.35978 |
| F | 3.08353 | -4.50921 | 2.86393 | H | 1.7331 | 2.10556 | -3.24584 |
| F | 3.79513 | -2.93088 | 0.86591 | H | 1.15295 | 0.94501 | -4.44428 |
| O | 1.32421 | 0.44327 | -0.34145 | H | 2.00216 | 0.3747 | -2.99694 |
| N | -3.3028 | -0.08695 | 0.07292 | H | -2.03887 | 1.96123 | -2.76986 |
| N | -1.25666 | 0.43498 | -0.172 | H | -1.23635 | 1.73263 | -4.33704 |
| C | -2.5272 | 0.90587 | -0.42625 | H | -0.69453 | 3.02275 | -3.26551 |
| C | -2.55697 | -1.14895 | 0.59002 | H | -0.61322 | -0.61708 | -4.01784 |
| C | -1.26166 | -0.83328 | 0.42209 | H | -1.6854 | -0.50711 | -2.6145 |
| C | -4.72987 | -0.03766 | 0.05188 | H | -0.06847 | -1.21171 | -2.4463 |
| C | -5.46139 | -1.21643 | -0.03421 | H | -8.55638 | 0.12585 | 0.01322 |
| C | -6.85654 | -1.17292 | -0.03974 | H | -4.93944 | -2.16176 | -0.12095 |
| C | -7.47113 | 0.07889 | 0.02169 | H | -4.7374 | 2.08342 | 0.17409 |
| C | -6.75043 | 1.27469 | 0.09113 | C | -7.71766 | -2.44063 | -0.11614 |
| C | -5.35944 | 1.19975 | 0.11632 | C | -8.58019 | -2.39605 | -1.39191 |
| C | 0.14638 | 2.98251 | -0.26943 | C | -6.8704 | -3.72033 | -0.15309 |
| C | 0.63242 | 2.97573 | 1.19061 | C | -8.6323 | -2.50606 | 1.12233 |
| C | 1.14481 | 3.77666 | -1.13476 | H | -9.24734 | -1.52791 | -1.40083 |
| C | -1.21725 | 3.69608 | -0.31443 | H | -7.95032 | -2.34555 | -2.28679 |
| C | -0.08159 | 0.98574 | -2.68998 | H | -9.20209 | -3.29625 | -1.46054 |
| C | 1.29132 | 1.11485 | -3.37077 | H | -6.2488 | -3.82168 | 0.74383 |
| C | -1.07488 | 1.99841 | -3.2861 | H | -7.52864 | -4.59449 | -0.19788 |
| C | -0.64388 | -0.42819 | -2.93913 | H | -6.21942 | -3.75362 | -1.03413 |
| C | 3.15246 | -1.14375 | -1.07513 | H | -9.24593 | -3.41407 | 1.0924 |
| C | 4.38356 | -1.04481 | -1.71599 | H | -8.04073 | -2.52283 | 2.04418 |
| C | 4.7053 | -1.76316 | -2.86442 | H | -9.31045 | -1.64822 | 1.17365 |
| C | 3.78544 | -2.64826 | -3.4049 | C | -7.5004 | 2.61142 | 0.147 |
| C | 2.56072 | -2.81738 | -2.7742 | C | -6.54452 | 3.81067 | 0.21447 |
| C | 2.29115 | -2.08728 | -1.62808 | C | -8.36829 | 2.75763 | -1.11771 |
| C | 3.62254 | 0.94766 | 0.67459 | C | -8.40084 | 2.63883 | 1.39674 |
| C | 3.99389 | 1.92857 | -0.24176 | H | -5.92119 | 3.78311 | 1.11532 |
| C | 4.71556 | 3.06658 | 0.07291 | H | -5.8861 | 3.85458 | -0.66026 |
| C | 5.13939 | 3.25379 | 1.38195 | H | -7.12313 | 4.74036 | 0.24175 |
| C | 4.83329 | 2.2926 | 2.33068 | H | -9.11117 | 1.95726 | -1.19671 |
| C | 4.09254 | 1.16865 | 1.96802 | H | -8.90691 | 3.71225 | -1.09872 |
| C | 2.21565 | -1.24973 | 1.44859 | H | -7.74839 | 2.73192 | -2.0206 |
| C | 2.81815 | -2.48758 | 1.66815 | H | -8.93843 | 3.59241 | 1.4558 |
| C | 2.47708 | -3.33217 | 2.71926 | H | -9.14573 | 1.83671 | 1.37918 |
| C | 1.50143 | -2.94291 | 3.62398 | H | -7.8054 | 2.52482 | 2.30909 |

**Supplementary Table 20.** Computed Cartesian coordinates (x, y, z) for the optimized structure of **2cB[1]**.

E1 = -3632.123208, G1 = -3631.485906, E2 = -3633.049463

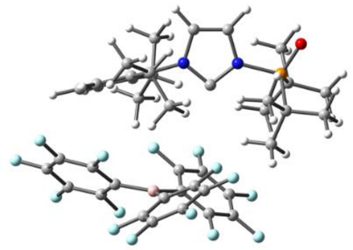

| | | | | | | | |
|---|---|---|---|---|---|---|---|
| P | 4.59168200 | 0.90715400 | -1.04816300 | C | -3.89976500 | 2.40700300 | 0.12110700 |
| F | -0.23304200 | -1.51311200 | 1.16599100 | C | -4.32680500 | 3.55926000 | -0.52213100 |
| F | 1.91938300 | -1.57817000 | 2.73348100 | C | -3.38612600 | 4.40291100 | -1.10039000 |
| F | 2.62835300 | 0.61888200 | 4.16689000 | C | -2.03443400 | 4.09111900 | -1.02020600 |
| F | 1.11809600 | 2.86849000 | 4.04951500 | C | -1.64452000 | 2.94308600 | -0.34929000 |
| F | -1.07695900 | 2.93681200 | 2.53920900 | C | -2.94870500 | -0.58110200 | 0.80687700 |
| F | -0.33384900 | 2.69756400 | -0.30365900 | C | -3.01315500 | -1.52739100 | 1.83647700 |
| F | -1.13061600 | 4.89172500 | -1.57747300 | C | -3.69713400 | -2.72778700 | 1.72600800 |
| F | -3.77749900 | 5.50236900 | -1.72605800 | C | -4.36214400 | -3.02207300 | 0.54358600 |
| F | -5.61941800 | 3.86169700 | -0.59145700 | C | -4.35337300 | -2.10530600 | -0.49614800 |
| F | -4.84398500 | 1.63605200 | 0.66453200 | C | -3.66758600 | -0.91115600 | -0.34622000 |
| F | -3.69460300 | -0.08836500 | -1.39947900 | B | -2.08614400 | 0.72774900 | 0.93144200 |
| F | -4.95761400 | -2.39898400 | -1.64549400 | H | 2.48214700 | -2.62806700 | -3.65776100 |
| F | -4.98470000 | -4.18297700 | 0.39836900 | H | 4.56993000 | -0.79542600 | -3.39017800 |
| F | -3.71419400 | -3.59656400 | 2.73174300 | H | -2.51477800 | -2.54259300 | -2.81520400 |
| F | -2.41036600 | -1.29696300 | 3.00472600 | H | -2.58903200 | -4.48349100 | -1.30885000 |
| O | 5.55305900 | 1.17561300 | -2.16133000 | H | -0.62284100 | -5.09337900 | 0.04223000 |
| N | 1.87329500 | -1.70390700 | -1.79235200 | H | 0.57498400 | -0.75476900 | -3.71104800 |
| N | 3.46998700 | -0.31775500 | -1.58680400 | H | 0.13166600 | 0.42836900 | -1.52478200 |
| C | 2.32070000 | -0.73031400 | -0.95352300 | H | -1.58736300 | 0.49683700 | -1.97093600 |
| C | 2.70252700 | -1.89400800 | -2.89839000 | H | -0.38823300 | 1.35908100 | -2.94469900 |
| C | 3.71270700 | -1.01283800 | -2.77339400 | H | -2.46987500 | -0.68222900 | -3.97935800 |
| C | 0.65165700 | -2.43830500 | -1.61955000 | H | -1.40916900 | -1.70073300 | -4.97644800 |
| C | -0.46554000 | -2.05007500 | -2.37623300 | H | -1.26814900 | 0.06618100 | -5.03125600 |
| C | -1.62889600 | -2.80849600 | -2.24836400 | H | 2.36021600 | -2.94329500 | 0.33264900 |
| C | -1.67359200 | -3.90377500 | -1.39356300 | H | 1.09821300 | -5.55488300 | 1.30066100 |
| C | -0.56247000 | -4.24921200 | -0.63666400 | H | 0.76639000 | -3.95003200 | 1.98985500 |
| C | 0.62549100 | -3.52177700 | -0.73119000 | H | 2.40088200 | -4.62871100 | 2.04838400 |
| C | -0.41578000 | -0.80200800 | -3.24583600 | H | 3.14274700 | -4.26360900 | -1.62401300 |
| C | -0.56878700 | 0.44645900 | -2.36482600 | H | 2.32778800 | -5.70606700 | -0.98908200 |
| C | -1.45228200 | -0.78844700 | -4.37202700 | H | 3.70187200 | -5.00163700 | -0.11430800 |
| C | 1.84796400 | -3.88121900 | 0.09913600 | H | 5.47572300 | -1.86222300 | -0.57256800 |
| C | 1.49832600 | -4.54311900 | 1.43457900 | H | 6.79837900 | -1.56801200 | 0.56794800 |
| C | 2.81214000 | -4.76332200 | -0.70818100 | H | 6.82956700 | -0.80668500 | -1.03810900 |
| C | 5.44240300 | 0.10516100 | 0.39751800 | H | 7.10606600 | 0.57388700 | 1.69293800 |
| C | 6.18033100 | -1.10540900 | -0.21058000 | H | 5.99103500 | 1.92656800 | 1.48657100 |
| C | 6.47431600 | 1.09464900 | 0.96351000 | H | 7.12043500 | 1.49968300 | 0.17843200 |
| C | 4.51526900 | -0.38869600 | 1.51307500 | H | 4.13156400 | 0.42949000 | 2.12527900 |
| C | 3.55423100 | 2.39852000 | -0.66806800 | H | 5.08292600 | -1.05002700 | 2.17861000 |
| C | 4.50955500 | 3.60279300 | -0.57746900 | H | 3.66414400 | -0.94680200 | 1.11781500 |
| C | 2.71909200 | 2.28082400 | 0.61066800 | H | 5.18000000 | 3.53979400 | 0.28483300 |
| C | 2.62509000 | 2.60388700 | -1.87998900 | H | 3.91296100 | 4.51597300 | -0.46546500 |
| C | -0.76069900 | 0.71261300 | 1.77087500 | H | 5.11937800 | 3.69228700 | -1.48033200 |
| C | -0.35521300 | 1.81324500 | 2.53354300 | H | 2.10943800 | 1.37268600 | 0.59652500 |
| C | 0.77151300 | 1.79780300 | 3.34061200 | H | 2.05093000 | 3.14702400 | 0.67942100 |
| C | 1.54473300 | 0.64591600 | 3.40286500 | H | 3.34839600 | 2.28170800 | 1.50659800 |
| C | 1.18819900 | -0.46940200 | 2.65919900 | H | 2.09002800 | 3.55269400 | -1.75717300 |
| C | 0.06256800 | -0.41493400 | 1.85429200 | H | 1.88108600 | 1.80846500 | -1.95463200 |
| C | -2.55273800 | 2.05168700 | 0.22907900 | H | 3.19328800 | 2.64593200 | -2.81474600 |

**Supplementary Table 21.** Computed Cartesian coordinates (x, y, z) for the optimized structure of **FLP-1aB[1]**.

E1 = -3633.291848, $\nu_{imag}$ = 7.33$i$ cm$^{-1}$, G1 = -3632.641226, E2 = -3634.219597

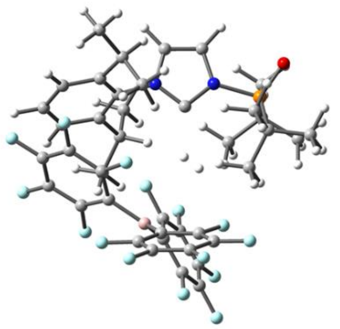

| | | | | | | | |
|---|---|---|---|---|---|---|---|
| P | -4.09611200 | -1.92847600 | -1.73350800 | C | 5.86093000 | -0.97283100 | -1.19815900 |
| F | -0.25390900 | 0.20289800 | 1.31850500 | C | 5.62211100 | -2.17299100 | -1.85398800 |
| F | -1.80369600 | -1.29975300 | 2.90327900 | C | 4.39069100 | -2.80221000 | -1.71835400 |
| F | -0.81622500 | -3.57165800 | 4.00345200 | C | 3.41820000 | -2.22315500 | -0.91770800 |
| F | 1.76222600 | -4.30432900 | 3.54971200 | C | 2.30775100 | 1.21053800 | 0.58887400 |
| F | 3.33221700 | -2.80172900 | 2.00723000 | C | 1.97466300 | 1.96617900 | 1.71822800 |
| F | 2.25500600 | -2.87799600 | -0.82593800 | C | 1.87181600 | 3.34798900 | 1.69829400 |
| F | 4.15962800 | -3.94992200 | -2.34781700 | C | 2.08607900 | 4.02603100 | 0.50662700 |
| F | 6.56633000 | -2.71829400 | -2.60607500 | C | 2.38717700 | 3.31888000 | -0.64760200 |
| F | 7.04117300 | -0.37371900 | -1.31885300 | C | 2.50620500 | 1.93990300 | -0.58778600 |
| F | 5.14149400 | 0.73622300 | 0.19519600 | B | 2.49197500 | -0.34581800 | 0.63825100 |
| F | 2.79602300 | 1.31670800 | -1.73147700 | H | -4.56696000 | 2.92756700 | -1.64430600 |
| F | 2.54099800 | 3.96723700 | -1.80048500 | H | -5.54060300 | 0.48348800 | -2.55801600 |
| F | 1.98924400 | 5.34734700 | 0.46451000 | H | -0.23964100 | 5.28016400 | -1.16535300 |
| F | 1.55910500 | 4.02433600 | 2.79945700 | H | -0.32191300 | 5.65762700 | 1.26512000 |
| F | 1.76496100 | 1.36934800 | 2.89452700 | H | -1.48813900 | 4.07678400 | 2.73394100 |
| O | -4.95394500 | -2.11621400 | -2.94263000 | H | -2.31058300 | 2.79059000 | -2.94051100 |
| N | -3.01246500 | 1.63830700 | -0.84514600 | H | -0.41696000 | 1.31781100 | -2.02859600 |
| N | -3.77043800 | -0.22814200 | -1.52437400 | H | 0.71207600 | 2.52078000 | -2.67710700 |
| C | -2.71891700 | 0.31129500 | -0.82025600 | H | -0.36536800 | 1.64767400 | -3.77625100 |
| C | -4.19683900 | 1.92045600 | -1.53037200 | H | -0.03092100 | 4.79922400 | -3.28361100 |
| C | -4.67496200 | 0.73951700 | -1.96765300 | H | -1.75586600 | 5.22192100 | -3.32386900 |
| C | -2.20723600 | 2.67412100 | -0.25905100 | H | -1.07212000 | 4.11807200 | -4.53145000 |
| C | -1.48143700 | 3.52207700 | -1.11450400 | H | -2.80892800 | 0.85304200 | 1.58451500 |
| C | -0.81016500 | 4.60135500 | -0.54012300 | H | -2.54113400 | 2.67554800 | 4.02526500 |
| C | -0.84742500 | 4.81224600 | 0.83286500 | H | -1.25827800 | 1.60926600 | 3.41465100 |
| C | -1.51347500 | 3.91983800 | 1.66116100 | H | -2.79094200 | 0.92918300 | 3.98785600 |
| C | -2.20570000 | 2.82736200 | 1.13532500 | H | -4.89507800 | 2.11651700 | 1.10187800 |
| C | -1.35557100 | 3.19923600 | -2.59625900 | H | -4.60930600 | 3.14883600 | 2.51685400 |
| C | -0.29337100 | 2.10115700 | -2.78188300 | H | -4.95083100 | 1.42044200 | 2.72988400 |
| C | -1.03695000 | 4.40977700 | -3.47653400 | H | -6.45214000 | -0.74068600 | -0.45202700 |
| C | -2.93336700 | 1.84080700 | 2.03612300 | H | -7.03421200 | -2.09521500 | 0.52535500 |
| C | -2.34133800 | 1.76641200 | 3.44609100 | H | -6.91647500 | -2.25925600 | -1.24517900 |
| C | -4.43633800 | 2.15142600 | 2.09545700 | H | -5.77053800 | -4.15547500 | 0.77317300 |
| C | -5.00889700 | -2.36866600 | -0.17128200 | H | -4.09927000 | -4.32759600 | 0.23132300 |
| C | -6.44086700 | -1.83219700 | -0.35891000 | H | -5.42909600 | -4.37436900 | -0.95039000 |
| C | -5.07123500 | -3.89864300 | -0.03137600 | H | -3.34987100 | -1.97532700 | 1.23115900 |
| C | -4.40732000 | -1.74268900 | 1.09354800 | H | -4.95130100 | -2.12067900 | 1.96810600 |
| C | -2.43464600 | -2.73589200 | -1.92257700 | H | -4.50644700 | -0.65330600 | 1.08400000 |
| C | -2.67959800 | -4.16431800 | -2.44623900 | H | -3.13857400 | -4.81372400 | -1.69585400 |
| C | -1.59046800 | -2.77134700 | -0.64099300 | H | -1.71270600 | -4.60582300 | -2.71552200 |
| C | -1.69279200 | -1.94770800 | -3.01885900 | H | -3.31745000 | -4.15680600 | -3.33446500 |
| C | 1.60531500 | -1.22913800 | 1.58048400 | H | -1.44882200 | -1.75998900 | -0.25224100 |
| C | 2.07013700 | -2.40436600 | 2.17964100 | H | -0.60897600 | -3.20235700 | -0.87309200 |
| C | 1.27617700 | -3.19842600 | 2.99464800 | H | -2.04690400 | -3.39880200 | 0.13229000 |
| C | -0.04165600 | -2.82478700 | 3.23157000 | H | -0.75605700 | -2.46955900 | -3.24843500 |
| C | -0.54291600 | -1.65961600 | 2.66567800 | H | -1.45116700 | -0.93963900 | -2.68101000 |
| C | 0.27730600 | -0.89040600 | 1.85658200 | H | -2.28523700 | -1.88489400 | -3.93721800 |
| C | 3.60508400 | -1.00922800 | -0.25295800 | H | 0.54493500 | -0.47194000 | -1.38418100 |
| C | 4.85659700 | -0.41356700 | -0.42179700 | H | 0.84778300 | -1.14111000 | -1.49048400 |

**Supplementary Table 22.** Computed Cartesian coordinates (x, y, z) for the optimized structure of **TS3a**.

E1 = -3633.295361, G1 = -3632.644662, E2 = -3634.224111

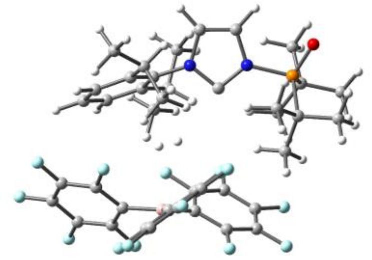

| | | | | | | | |
|---|---|---|---|---|---|---|---|
| P | 4.66933 | 0.46603 | -0.83011 | C | -3.66804 | 3.7907 | -0.82145 |
| F | -0.60041 | -1.60638 | 1.69825 | C | -2.56996 | 4.37563 | -1.44125 |
| F | 1.60027 | -1.81582 | 3.18363 | C | -1.29759 | 3.86456 | -1.21975 |
| F | 2.64162 | 0.38727 | 4.39658 | C | -1.14384 | 2.78127 | -0.36852 |
| F | 1.428 | 2.79643 | 4.11753 | C | -3.07481 | -0.31463 | 1.01259 |
| F | -0.77497 | 3.02936 | 2.62458 | C | -3.43137 | -1.1106 | 2.10354 |
| F | 0.10046 | 2.33658 | -0.18748 | C | -4.29937 | -2.18721 | 2.00179 |
| F | -0.24288 | 4.41702 | -1.81259 | C | -4.84287 | -2.50491 | 0.76316 |
| F | -2.73726 | 5.41954 | -2.23954 | C | -4.52955 | -1.73339 | -0.34709 |
| F | -4.88529 | 4.28453 | -1.02626 | C | -3.66769 | -0.65814 | -0.20518 |
| F | -4.5634 | 2.16306 | 0.5701 | B | -2.02561 | 0.85088 | 1.12706 |
| F | -3.38826 | 0.0288 | -1.31609 | H | 2.42171 | -2.55449 | -3.92551 |
| F | -5.03928 | -2.04516 | -1.53698 | H | 4.67763 | -1.03027 | -3.31701 |
| F | -5.6567 | -3.54346 | 0.63987 | H | -2.63091 | -2.06923 | -3.48291 |
| F | -4.60866 | -2.91689 | 3.0693 | H | -3.09152 | -3.79875 | -1.80323 |
| F | -2.93889 | -0.84686 | 3.31583 | H | -1.33928 | -4.524 | -0.24165 |
| O | 5.76977 | 0.7069 | -1.81271 | H | 0.75375 | -0.77541 | -4.24613 |
| N | 1.71712 | -1.64016 | -2.08775 | H | 0.20726 | 0.84352 | -2.48166 |
| N | 3.44544 | -0.49653 | -1.61843 | H | -1.5061 | 0.79446 | -2.9251 |
| C | 2.18733 | -0.77982 | -1.14513 | H | -0.31174 | 1.46233 | -4.05915 |
| C | 2.63791 | -1.88534 | -3.10723 | H | -2.19587 | -0.61343 | -5.0266 |
| C | 3.73206 | -1.15702 | -2.81471 | H | -1.07147 | -1.83862 | -5.64522 |
| C | 0.41955 | -2.25156 | -2.02591 | H | -0.78669 | -0.11638 | -5.96055 |
| C | -0.56555 | -1.81664 | -2.92677 | H | 1.70048 | -2.7408 | 0.34248 |
| C | -1.83449 | -2.38962 | -2.82041 | H | 0.37754 | -5.45425 | 0.82782 |
| C | -2.1001 | -3.36011 | -1.86433 | H | -0.06833 | -3.94516 | 1.65574 |
| C | -1.10602 | -3.77111 | -0.98602 | H | 1.55175 | -4.61894 | 1.84835 |
| C | 0.177 | -3.2263 | -1.0411 | H | 2.86366 | -3.88917 | -1.55263 |
| C | -0.2947 | -0.70791 | -3.94012 | H | 1.97199 | -5.37773 | -1.19282 |
| C | -0.48657 | 0.68159 | -3.31029 | H | 3.14583 | -4.7381 | -0.02789 |
| C | -1.13894 | -0.83469 | -5.21295 | H | 5.0325 | -2.46973 | -0.60712 |
| C | 1.26211 | -3.65928 | -0.05983 | H | 6.29914 | -2.50699 | 0.62975 |
| C | 0.73807 | -4.46432 | 1.13259 | H | 6.5651 | -1.60838 | -0.88034 |
| C | 2.37672 | -4.45702 | -0.75603 | H | 6.82057 | -0.5844 | 2.03518 |
| C | 5.23669 | -0.6266 | 0.56534 | H | 5.97923 | 0.96026 | 1.88206 |
| C | 5.81863 | -1.87633 | -0.12743 | H | 7.14059 | 0.45649 | 0.63163 |
| C | 6.3623 | 0.10475 | 1.3158 | H | 3.90245 | -0.29039 | 2.2732 |
| C | 4.14334 | -1.06835 | 1.5468 | H | 4.50116 | -1.93661 | 2.11241 |
| C | 3.82306 | 2.04019 | -0.333 | H | 3.22293 | -1.35232 | 1.03072 |
| C | 4.93031 | 3.07866 | -0.07234 | H | 5.53787 | 2.827 | 0.80268 |
| C | 2.92324 | 1.90808 | 0.90042 | H | 4.45999 | 4.05045 | 0.11902 |
| C | 2.99381 | 2.49996 | -1.54699 | H | 5.59555 | 3.1734 | -0.93438 |
| C | -0.79342 | 0.72089 | 2.0925 | H | 2.19451 | 1.10158 | 0.77738 |
| C | -0.22599 | 1.81818 | 2.74245 | H | 2.37785 | 2.84827 | 1.04258 |
| C | 0.91061 | 1.7209 | 3.53115 | H | 3.50859 | 1.7334 | 1.80894 |
| C | 1.53046 | 0.48734 | 3.67846 | H | 2.57948 | 3.49309 | -1.34085 |
| C | 1.00288 | -0.63401 | 3.05308 | H | 2.15858 | 1.82415 | -1.73905 |
| C | -0.14281 | -0.50114 | 2.28604 | H | 3.61173 | 2.56408 | -2.44854 |
| C | -2.21728 | 2.14419 | 0.25579 | H | -0.22553 | -0.15007 | -0.48169 |
| C | -3.47642 | 2.69123 | 0.00189 | H | -0.94124 | -0.27688 | -0.64635 |

**Supplementary Table 23.** Computed Cartesian coordinates (x, y, z) for the optimized structure of **6aB[1]**.

E1 = -3633.293398, $\nu_{\mathrm{imag}}$ = 322.9$i$ cm$^{-1}$, G1 = -3632.639005, E2 = -3634.222025

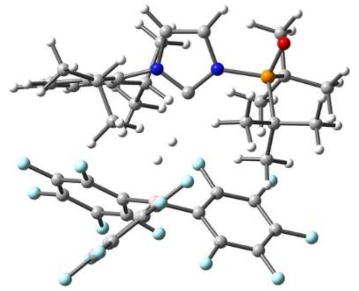

| | | | |
|---|---|---|---|
| P | 4.31921 | -1.23864 | -0.44076 |
| F | 0.4763 | 1.02537 | 2.38523 |
| F | 2.42295 | 2.66468 | 3.20175 |
| F | 3.11553 | 4.82209 | 1.69334 |
| F | 1.78632 | 5.33216 | -0.62525 |
| F | -0.18394 | 3.75102 | -1.42904 |
| F | 0.52544 | 0.73972 | -2.31445 |
| F | -0.2696 | 0.6777 | -4.86073 |
| F | -2.84485 | 1.32603 | -5.50319 |
| F | -4.58821 | 2.01849 | -3.53318 |
| F | -3.81293 | 2.05216 | -0.98851 |
| F | -2.92349 | -0.89975 | -0.27181 |
| F | -4.93873 | -1.53986 | 1.38355 |
| F | -5.33459 | -0.11627 | 3.65801 |
| F | -3.74298 | 2.01965 | 4.21533 |
| F | -1.80072 | 2.72442 | 2.55462 |
| O | 5.37391 | -2.16398 | -0.95176 |
| N | 0.85343 | -2.95434 | -0.06019 |
| N | 2.81607 | -2.16511 | -0.37095 |
| C | 1.5207 | -1.78264 | -0.15342 |
| C | 1.69779 | -4.04964 | -0.21618 |
| C | 2.93237 | -3.55559 | -0.41966 |
| C | -0.54236 | -3.13987 | 0.24194 |
| C | -1.37111 | -3.64809 | -0.77023 |
| C | -2.66911 | -4.01093 | -0.41382 |
| C | -3.12 | -3.85374 | 0.88917 |
| C | -2.29689 | -3.28771 | 1.85331 |
| C | -0.98754 | -2.91009 | 1.55311 |
| C | -0.89706 | -3.70218 | -2.21663 |
| C | -0.91553 | -2.28065 | -2.80221 |
| C | -1.70407 | -4.65772 | -3.0986 |
| C | -0.08577 | -2.26765 | 2.59919 |
| C | -0.86012 | -1.57748 | 3.72485 |
| C | 0.91898 | -3.27319 | 3.18235 |
| C | 4.61121 | -0.78411 | 1.33444 |
| C | 5.00458 | -2.09588 | 2.03994 |
| C | 5.7968 | 0.19508 | 1.39058 |
| C | 3.39416 | -0.18212 | 2.03973 |
| C | 4.00867 | 0.15899 | -1.61303 |
| C | 5.38977 | 0.6866 | -2.04925 |
| C | 3.1737 | 1.30146 | -1.02673 |
| C | 3.31355 | -0.45269 | -2.84491 |
| C | 0.05111 | 2.29985 | 0.43987 |
| C | 0.43272 | 3.42361 | -0.28982 |
| C | 1.4558 | 4.27593 | 0.11411 |
| C | 2.13398 | 4.02063 | 1.296 |
| C | 1.77741 | 2.91919 | 2.06375 |
| C | 0.76235 | 2.08793 | 1.61978 |
| C | -1.60928 | 1.35352 | -1.52822 |
| C | -2.90666 | 1.69218 | -1.90479 |
| C | -3.33492 | 1.69001 | -3.22735 |
| C | -2.44676 | 1.34277 | -4.23587 |
| C | -1.13731 | 1.01902 | -3.90889 |
| C | -0.75298 | 1.04954 | -2.57846 |
| C | -2.24008 | 0.91528 | 1.07345 |
| C | -2.50846 | 1.63523 | 2.23658 |
| C | -3.53123 | 1.29864 | 3.11639 |
| C | -4.34627 | 0.21265 | 2.83377 |
| C | -4.13305 | -0.5192 | 1.67352 |
| C | -3.08955 | -0.16501 | 0.83283 |
| B | -1.11612 | 1.30101 | -0.00244 |
| H | 1.32724 | -5.06102 | -0.16314 |
| H | 3.88442 | -4.02872 | -0.59793 |
| H | -3.34227 | -4.41299 | -1.1627 |
| H | -4.13313 | -4.14188 | 1.14856 |
| H | -2.68529 | -3.13398 | 2.8541 |
| H | 0.14248 | -4.04889 | -2.23047 |
| H | -0.25742 | -1.61604 | -2.2369 |
| H | -1.92783 | -1.86294 | -2.76463 |
| H | -0.57927 | -2.28254 | -3.84504 |
| H | -2.72154 | -4.28822 | -3.26927 |
| H | -1.77166 | -5.65854 | -2.65891 |
| H | -1.22391 | -4.748 | -4.07811 |
| H | 0.4805 | -1.48222 | 2.09048 |
| H | -1.34949 | -2.29826 | 4.39003 |
| H | -1.62164 | -0.89949 | 3.32876 |
| H | -0.16981 | -0.98508 | 4.33302 |
| H | 1.55635 | -3.71515 | 2.41025 |
| H | 0.39453 | -4.08782 | 3.69526 |
| H | 1.56986 | -2.776 | 3.91045 |
| H | 4.1837 | -2.82182 | 2.0276 |
| H | 5.2344 | -1.8744 | 3.08881 |
| H | 5.88103 | -2.55544 | 1.57637 |
| H | 6.09426 | 0.33273 | 2.43642 |
| H | 5.53231 | 1.18092 | 0.99488 |
| H | 6.66292 | -0.18569 | 0.83984 |
| H | 3.04082 | 0.72274 | 1.54815 |
| H | 3.66934 | 0.08552 | 3.06592 |
| H | 2.56375 | -0.88992 | 2.08919 |
| H | 5.9355 | 1.15592 | -1.2257 |
| H | 5.24005 | 1.44803 | -2.82359 |
| H | 6.00913 | -0.11415 | -2.46098 |
| H | 2.22544 | 0.94969 | -0.61819 |
| H | 2.94167 | 2.01852 | -1.82216 |
| H | 3.71742 | 1.84218 | -0.24516 |
| H | 3.23472 | 0.31715 | -3.62057 |
| H | 2.30345 | -0.79795 | -2.61998 |
| H | 3.8938 | -1.28848 | -3.24839 |
| H | 0.30011 | -0.42083 | -0.07526 |
| H | -0.43709 | -0.02458 | 0. |

**Supplementary Table 24.** Computed Cartesian coordinates (x, y, z) for the optimized structure of **TS4a**.

E1 = -3633.362975, G1 = -3632.702965, E2 = -3634.301313

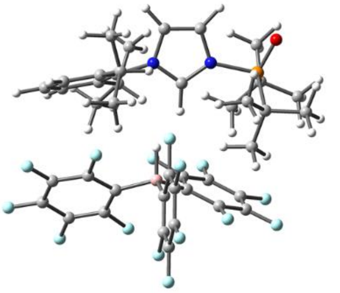

| | | | | | | | |
|---|---|---|---|---|---|---|---|
| P | 4.20202 | 1.13892 | -0.25621 | C | -1.62394 | -1.33881 | 4.17133 |
| F | -0.52898 | -2.05802 | -2.36753 | C | -1.13626 | -0.13492 | 4.66795 |
| F | 1.1433 | -3.93705 | -3.22961 | C | -0.55295 | 0.7699 | 3.79494 |
| F | 2.75801 | -5.25976 | -1.47085 | C | -0.47692 | 0.44949 | 2.44599 |
| F | 2.63144 | -4.63557 | 1.1846 | C | -2.41469 | -1.13922 | -0.24601 |
| F | 0.95209 | -2.81315 | 2.07036 | C | -3.08161 | -2.23761 | -0.77711 |
| F | 0.09111 | 1.39931 | 1.65798 | C | -4.3776 | -2.17438 | -1.28106 |
| F | -0.07642 | 1.93623 | 4.24681 | C | -5.07341 | -0.97568 | -1.23589 |
| F | -1.22662 | 0.14448 | 5.96758 | C | -4.46413 | 0.14022 | -0.67932 |
| F | -2.1862 | -2.21486 | 5.00418 | C | -3.16612 | 0.03317 | -0.20026 |
| F | -1.99118 | -2.78542 | 2.38697 | B | -0.87875 | -1.08553 | 0.30654 |
| F | -2.63276 | 1.14462 | 0.33667 | H | 1.32357 | 4.75763 | -1.97184 |
| F | -5.13584 | 1.29489 | -0.60534 | H | 3.88195 | 3.79359 | -1.36841 |
| F | -6.31809 | -0.89741 | -1.70783 | H | -3.19032 | 4.81771 | -0.07312 |
| F | -4.96053 | -3.25778 | -1.79789 | H | -4.3917 | 3.67525 | -1.89641 |
| F | -2.48973 | -3.43848 | -0.83476 | H | -3.25685 | 2.00471 | -3.28073 |
| O | 5.33437 | 2.09966 | -0.36779 | H | 0.38187 | 4.06421 | 0.81647 |
| N | 0.76128 | 2.82934 | -1.21113 | H | -1.58845 | 3.17865 | 2.11468 |
| N | 2.74727 | 2.07526 | -0.76723 | H | -2.32169 | 4.78934 | 2.03533 |
| C | 1.43987 | 1.78784 | -0.7397 | H | -0.77619 | 4.56184 | 2.86617 |
| C | 1.66153 | 3.82315 | -1.55552 | H | -1.38391 | 6.52732 | 0.37317 |
| C | 2.90068 | 3.35699 | -1.27268 | H | 0.17482 | 6.2396 | -0.40957 |
| C | -0.67354 | 2.96563 | -1.34249 | H | 0.09466 | 6.47171 | 1.34659 |
| C | -1.3077 | 3.8669 | -0.48089 | H | -0.07031 | 0.54359 | -2.50348 |
| C | -2.65943 | 4.12241 | -0.7157 | H | -2.1715 | 0.76115 | -4.69496 |
| C | -3.3377 | 3.47683 | -1.7355 | H | -2.33715 | -0.19042 | -3.19575 |
| C | -2.68921 | 2.53448 | -2.52552 | H | -1.06917 | -0.5783 | -4.36018 |
| C | -1.33763 | 2.24606 | -2.35008 | H | 1.11459 | 2.40004 | -3.74445 |
| C | -0.60818 | 4.518 | 0.70261 | H | -0.21007 | 2.43352 | -4.91746 |
| C | -1.37293 | 4.24278 | 2.00591 | H | 0.82261 | 0.99896 | -4.78408 |
| C | -0.4167 | 6.02514 | 0.48736 | H | 3.99352 | 1.20748 | -3.17583 |
| C | -0.64002 | 1.18126 | -3.19014 | H | 4.81066 | -0.3018 | -3.60191 |
| C | -1.62135 | 0.24456 | -3.89982 | H | 5.68883 | 0.9607 | -2.70598 |
| C | 0.33184 | 1.79367 | -4.21197 | H | 5.49299 | -1.94186 | -1.87939 |
| C | 4.26037 | -0.20859 | -1.52076 | H | 4.97732 | -1.80924 | -0.19876 |
| C | 4.72178 | 0.4666 | -2.82591 | H | 6.27166 | -0.75389 | -0.82249 |
| C | 5.31664 | -1.23166 | -1.06407 | H | 2.50673 | -1.36333 | -0.85335 |
| C | 2.92056 | -0.91364 | -1.7573 | H | 3.07506 | -1.72121 | -2.48119 |
| C | 3.83979 | 0.65237 | 1.49163 | H | 2.16958 | -0.24532 | -2.19081 |
| C | 5.20073 | 0.26822 | 2.10939 | H | 5.62907 | -0.62884 | 1.65325 |
| C | 2.84901 | -0.50551 | 1.64906 | H | 5.04337 | 0.05388 | 3.17212 |
| C | 3.3083 | 1.90813 | 2.21255 | H | 5.92464 | 1.08249 | 2.02192 |
| C | 0.0705 | -2.35477 | -0.09798 | H | 1.88602 | -0.3196 | 1.1689 |
| C | 0.9164 | -3.06238 | 0.74987 | H | 2.64143 | -0.6495 | 2.71433 |
| C | 1.82069 | -4.02894 | 0.31361 | H | 3.24768 | -1.45002 | 1.26821 |
| C | 1.89027 | -4.34794 | -1.03211 | H | 3.31963 | 1.71447 | 3.29067 |
| C | 1.05863 | -3.68096 | -1.92099 | H | 2.27428 | 2.13171 | 1.94158 |
| C | 0.19396 | -2.71029 | -1.44054 | H | 3.93596 | 2.78448 | 2.02139 |
| C | -0.9276 | -0.74019 | 1.89976 | H | 0.96646 | 0.87732 | -0.39173 |
| C | -1.51489 | -1.60955 | 2.81422 | H | -0.38178 | -0.1343 | -0.27484 |

**Supplementary Table 25.** Computed Cartesian coordinates (x, y, z) for the optimized structure of **5aB$^{1}$**.

E1 = -3633.280758, $\nu_{imag}$ = 149.8$i$ cm$^{-1}$, G1 = -3632.634021, E2 = -3634.207909

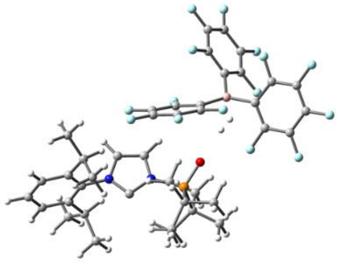

| | | | |
|---|---|---|---|
| P | 1.45507 | 1.9576 | -0.93881 |
| F | -0.5549 | -1.87579 | -0.45638 |
| F | 1.39287 | -2.37266 | 1.31847 |
| F | 1.2895 | -1.2628 | 3.79782 |
| F | -0.80873 | 0.31491 | 4.49267 |
| F | -2.76645 | 0.79837 | 2.76224 |
| F | -2.49652 | 2.54624 | -0.09077 |
| F | -4.35623 | 4.42979 | 0.30829 |
| F | -6.91383 | 3.71682 | 0.90797 |
| F | -7.5761 | 1.08557 | 1.10081 |
| F | -5.73054 | -0.80237 | 0.70487 |
| F | -4.0852 | -0.32645 | -2.6195 |
| F | -5.04366 | -2.45132 | -3.94727 |
| F | -4.96243 | -4.92138 | -2.80544 |
| F | -3.90788 | -5.23093 | -0.32022 |
| F | -2.95076 | -3.12948 | 1.0129 |
| O | 0.08379 | 1.35289 | -1.01881 |
| N | 4.34223 | -0.48849 | -0.45571 |
| N | 2.59443 | 0.66012 | -0.80209 |
| C | 3.87793 | 0.77875 | -0.31935 |
| C | 3.40276 | -1.3639 | -0.99856 |
| C | 2.28995 | -0.63885 | -1.21916 |
| C | 5.63535 | -0.91657 | -0.00664 |
| C | 5.75041 | -1.41374 | 1.29831 |
| C | 7.00958 | -1.8484 | 1.71365 |
| C | 8.10143 | -1.78731 | 0.85704 |
| C | 7.95877 | -1.28153 | -0.42985 |
| C | 6.71945 | -0.83603 | -0.89095 |
| C | 4.56498 | -1.4055 | 2.24925 |
| C | 4.60425 | -0.13255 | 3.10865 |
| C | 4.47422 | -2.66305 | 3.1188 |
| C | 6.5562 | -0.21082 | -2.26764 |
| C | 6.80609 | 1.30324 | -2.17399 |
| C | 7.44067 | -0.85146 | -3.3415 |
| C | 1.91905 | 2.78072 | -2.53832 |
| C | 1.44051 | 1.82181 | -3.64624 |
| C | 1.15911 | 4.11117 | -2.66524 |
| C | 3.42872 | 3.01013 | -2.70012 |
| C | 1.6654 | 2.99196 | 0.58752 |
| C | 0.44727 | 3.93012 | 0.68747 |
| C | 2.96457 | 3.80676 | 0.63632 |
| C | 1.60389 | 2.01116 | 1.77172 |
| C | -1.74833 | -0.514 | 1.06216 |
| C | -1.75817 | 0.02872 | 2.34618 |
| C | -0.75405 | -0.22083 | 3.27646 |
| C | 0.31714 | -1.02661 | 2.92321 |
| C | 0.37482 | -1.5809 | 1.65086 |
| C | -0.64439 | -1.31137 | 0.75346 |
| C | -4.03644 | 0.79302 | 0.25667 |
| C | -5.35554 | 0.47502 | 0.57165 |
| C | -6.33069 | 1.43912 | 0.79456 |
| C | -5.99474 | 2.78247 | 0.70016 |
| C | -4.68862 | 3.14403 | 0.39525 |
| C | -3.74184 | 2.15184 | 0.19068 |
| C | -3.44873 | -1.63759 | -0.76157 |
| C | -3.43519 | -2.91884 | -0.21541 |
| C | -3.93783 | -4.02682 | -0.88478 |
| C | -4.48242 | -3.87076 | -2.15244 |
| C | -4.52338 | -2.6101 | -2.73348 |
| C | -4.02266 | -1.52723 | -2.02594 |
| B | -2.95077 | -0.35007 | 0.03879 |
| H | 3.61249 | -2.41139 | -1.14673 |
| H | 1.31436 | -0.90908 | -1.58763 |
| H | 7.13888 | -2.2377 | 2.71897 |
| H | 9.07376 | -2.13269 | 1.19644 |
| H | 8.82504 | -1.23414 | -1.08204 |
| H | 3.64973 | -1.3676 | 1.65266 |
| H | 4.64629 | 0.7582 | 2.47287 |
| H | 5.48803 | -0.13005 | 3.75796 |
| H | 3.71198 | -0.07282 | 3.74128 |
| H | 5.28166 | -2.71123 | 3.85826 |
| H | 4.51426 | -3.57263 | 2.51026 |
| H | 3.52653 | -2.66028 | 3.6653 |
| H | 5.51433 | -0.35431 | -2.57649 |
| H | 7.83934 | 1.50109 | -1.86509 |
| H | 6.13274 | 1.75759 | -1.44008 |
| H | 6.64261 | 1.78295 | -3.14641 |
| H | 7.30537 | -1.9375 | -3.37908 |
| H | 8.50358 | -0.64626 | -3.17185 |
| H | 7.18715 | -0.4406 | -4.32457 |
| H | 1.95776 | 0.85742 | -3.5947 |
| H | 1.66581 | 2.26973 | -4.62118 |
| H | 0.36445 | 1.64172 | -3.58372 |
| H | 1.28088 | 4.49715 | -3.68416 |
| H | 1.54858 | 4.87001 | -1.97951 |
| H | 0.08734 | 3.98514 | -2.47903 |
| H | 3.84944 | 3.65009 | -1.92141 |
| H | 3.60394 | 3.4968 | -3.66743 |
| H | 3.98145 | 2.06734 | -2.69054 |
| H | 0.43119 | 4.6863 | -0.10139 |
| H | 0.50237 | 4.45873 | 1.64638 |
| H | -0.49226 | 3.37608 | 0.65452 |
| H | 3.83866 | 3.16363 | 0.50705 |
| H | 3.02911 | 4.29788 | 1.61497 |
| H | 2.97621 | 4.59501 | -0.12355 |
| H | 1.57137 | 2.58229 | 2.70682 |
| H | 2.4745 | 1.35209 | 1.80281 |
| H | 0.69691 | 1.40379 | 1.71437 |
| H | -1.684 | 0.57269 | -1.12074 |
| H | -2.31042 | 0.29825 | -1.46158 |

**Supplementary Table 26.** Computed Cartesian coordinates (x, y, z) for the optimized structure of **TS5a**.

E1 = -3633.288363, G1 = -3632.635317, E2 = -3634.217891

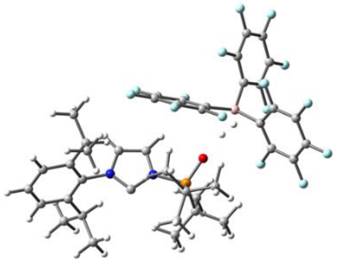

| | | | | | | | |
|---|---|---|---|---|---|---|---|
| P | -1.22611800 | 1.90125600 | 0.41877500 | C | 5.31167800 | 1.44654800 | -2.07959800 |
| F | 0.59257800 | -1.78452000 | 1.43422700 | C | 5.13952500 | 2.80820500 | -1.85813200 |
| F | -1.26061900 | -3.28201400 | 0.19161400 | C | 4.16501700 | 3.23933100 | -0.97076200 |
| F | -1.30764400 | -3.40104500 | -2.53594500 | C | 3.37589400 | 2.29902400 | -0.31936500 |
| F | 0.44309900 | -1.91014200 | -3.97751500 | C | 3.54471800 | -1.12554900 | 1.17810600 |
| F | 2.20659800 | -0.34207900 | -2.77124300 | C | 3.90795400 | -2.43065100 | 0.86202100 |
| F | 2.46775800 | 2.77559200 | 0.54845100 | C | 4.76044900 | -3.18530400 | 1.66031600 |
| F | 3.99299300 | 4.54153100 | -0.74497100 | C | 5.28314700 | -2.63230800 | 2.82121200 |
| F | 5.90624900 | 3.69011800 | -2.49115300 | C | 4.94998900 | -1.33015600 | 3.17219800 |
| F | 6.24944100 | 1.02618300 | -2.92531000 | C | 4.10083000 | -0.61093100 | 2.34476800 |
| F | 4.69414800 | -0.75759800 | -1.65203800 | B | 2.61850100 | -0.19027900 | 0.23020600 |
| F | 3.79686600 | 0.64747900 | 2.71015700 | H | -3.83431300 | -1.93571200 | 1.90956000 |
| F | 5.44467200 | -0.79051700 | 4.28432500 | H | -1.39300700 | -0.61136200 | 2.00542500 |
| F | 6.09777300 | -3.34365000 | 3.59307400 | H | -7.10807400 | -2.81217000 | -1.89613700 |
| F | 5.08081900 | -4.43130700 | 1.31852700 | H | -9.12795600 | -1.83316500 | -0.87393300 |
| F | 3.44913400 | -3.02056100 | -0.24602500 | H | -8.93169400 | -0.10122400 | 0.86548300 |
| O | 0.04950900 | 1.23528500 | 0.87456800 | H | -3.60952200 | -1.77103800 | -0.95483900 |
| N | -4.34836900 | -0.22601200 | 0.67664300 | H | -4.53364700 | -0.86291400 | -3.08030600 |
| N | -2.49608100 | 0.79642100 | 0.76477200 | H | -5.29999800 | -2.42383300 | -3.41717700 |
| C | -3.75166700 | 0.89833300 | 0.20955300 | H | -3.53396800 | -2.31182100 | -3.32989900 |
| C | -3.51091000 | -1.00167400 | 1.47822500 | H | -5.24227400 | -4.28638900 | -1.55024700 |
| C | -2.32882400 | -0.36011300 | 1.53434600 | H | -4.39686400 | -3.94235800 | -0.03241500 |
| C | -5.65183700 | -0.65539600 | 0.26194200 | H | -3.47472100 | -4.14624400 | -1.53062700 |
| C | -5.73325200 | -1.63673300 | -0.73438800 | H | -5.65281000 | 0.91936400 | 2.36069200 |
| C | -7.00735400 | -2.05186000 | -1.12659000 | H | -7.69694100 | 2.53285400 | 0.75560500 |
| C | -8.14495600 | -1.50114800 | -0.55278100 | H | -5.94465900 | 2.47595500 | 0.46139200 |
| C | -8.03189300 | -0.52453700 | 0.43033800 | H | -6.57668700 | 3.18896500 | 1.96690500 |
| C | -6.78160500 | -0.08466500 | 0.86466800 | H | -7.66272700 | -0.09852700 | 3.49323400 |
| C | -4.49820400 | -2.23751200 | -1.38971200 | H | -8.69179000 | 1.10204800 | 2.69060400 |
| C | -4.46681800 | -1.94000500 | -2.89449700 | H | -7.44790000 | 1.63207200 | 3.82091900 |
| C | -4.39898600 | -3.74275500 | -1.10926600 | H | -2.07080800 | 2.13307100 | 3.20085900 |
| C | -6.64654000 | 1.01243300 | 1.90835400 | H | -1.48986800 | 3.75742500 | 3.59508500 |
| C | -6.71884900 | 2.38940800 | 1.23009600 | H | -0.33855400 | 2.51065300 | 3.07070300 |
| C | -7.67345500 | 0.89806500 | 3.03992100 | H | -0.61354300 | 5.28147800 | 1.87006100 |
| C | -1.56134800 | 3.38500900 | 1.47058200 | H | -0.69252300 | 4.90840800 | 0.14648700 |
| C | -1.34633900 | 2.90563300 | 2.92075400 | H | 0.49802800 | 4.08799200 | 1.18002800 |
| C | -0.52483900 | 4.47211200 | 1.13641400 | H | -3.18397700 | 4.36509400 | 0.35118900 |
| C | -2.98751600 | 3.93973700 | 1.33717500 | H | -3.11335200 | 4.73998600 | 2.07622600 |
| C | -1.24254200 | 2.16397100 | -1.41532600 | H | -3.74289600 | 3.17347000 | 1.52907700 |
| C | 0.18302700 | 2.58800600 | -1.82143500 | H | 0.53081700 | 3.48187800 | -1.29595000 |
| C | -2.26853500 | 3.20324400 | -1.88663800 | H | 0.18156000 | 2.80878200 | -2.89474700 |
| C | -1.56498600 | 0.80657500 | -2.06851500 | H | 0.90484700 | 1.78664300 | -1.64702800 |
| C | 1.49992600 | -1.01078900 | -0.60225000 | H | -3.27384400 | 2.94450400 | -1.54254600 |
| C | 1.41296400 | -1.07967100 | -1.98806900 | H | -2.27115300 | 3.20867200 | -2.98310600 |
| C | 0.49101700 | -1.88692000 | -2.64764600 | H | -2.01674300 | 4.21596200 | -1.55647600 |
| C | -0.40971000 | -2.64027700 | -1.91229700 | H | -1.26153500 | 0.83620800 | -3.12114500 |
| C | -0.38371100 | -2.57912100 | -0.52471100 | H | -2.63455000 | 0.59067000 | -2.01446800 |
| C | 0.57501700 | -1.78936600 | 0.09080400 | H | -1.02550600 | -0.01058000 | -1.58552600 |
| C | 3.49397000 | 0.93143600 | -0.52945200 | H | 1.42283800 | 0.62797400 | 0.87081300 |
| C | 4.49783700 | 0.54401900 | -1.41184800 | H | 2.13013900 | 0.47550600 | 1.29043600 |

**Supplementary Table 27.** Computed Cartesian coordinates (x, y, z) for the optimized structure of **7aB[1]**.

E1 = -3633.288355, $\nu_{imag}$ = 219.5$i$ cm$^{-1}$, G1 = -3632.637504, E2 = -3634.218176

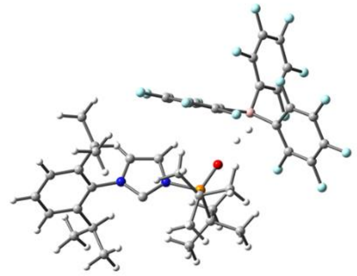

| | | | |
|---|---|---|---|
| P | -1.21932500 | 1.90010000 | 0.40539700 |
| F | 0.58725700 | -1.79350900 | 1.43741200 |
| F | -1.26437300 | -3.29486200 | 0.19872400 |
| F | -1.31187400 | -3.42062600 | -2.52899500 |
| F | 0.43706500 | -1.93046100 | -3.97395000 |
| F | 2.19685800 | -0.35574600 | -2.77179900 |
| F | 2.45654600 | 2.77528800 | 0.55215400 |
| F | 3.97949500 | 4.54034900 | -0.74347300 |
| F | 5.88970800 | 3.68898500 | -2.49364700 |
| F | 6.23124600 | 1.02471100 | -2.92872200 |
| F | 4.67791900 | -0.75855900 | -1.65315900 |
| F | 3.80144800 | 0.65893800 | 2.70400800 |
| F | 5.45817200 | -0.77006700 | 4.27319200 |
| F | 6.11021200 | -3.32563600 | 3.59053300 |
| F | 5.08124100 | -4.42497000 | 1.32642500 |
| F | 3.43940000 | -3.02337900 | -0.23473500 |
| O | 0.05670900 | 1.23164800 | 0.86052700 |
| N | -4.34063700 | -0.22115000 | 0.68217400 |
| N | -2.48679900 | 0.79862700 | 0.76369000 |
| C | -3.74352500 | 0.90064500 | 0.21042500 |
| C | -3.50280600 | -0.99545800 | 1.48472900 |
| C | -2.31951500 | -0.35596300 | 1.53666800 |
| C | -5.64621000 | -0.64898600 | 0.27214000 |
| C | -5.73229900 | -1.63272700 | -0.72135900 |
| C | -7.00845600 | -2.04520100 | -1.10969700 |
| C | -8.14321400 | -1.48983200 | -0.53474300 |
| C | -8.02534500 | -0.51122500 | 0.44584300 |
| C | -6.77289200 | -0.07370300 | 0.87626900 |
| C | -4.50028000 | -2.23856500 | -1.37770500 |
| C | -4.47066600 | -1.94443900 | -2.88319200 |
| C | -4.40476800 | -3.74349900 | -1.09429100 |
| C | -6.63258500 | 1.02520400 | 1.91739000 |
| C | -6.70212100 | 2.40109600 | 1.23662100 |
| C | -7.65756900 | 0.91619800 | 3.05125700 |
| C | -1.54309000 | 3.38979000 | 1.45095000 |
| C | -1.31262600 | 2.92144700 | 2.90226300 |
| C | -0.51326100 | 4.47761800 | 1.09874200 |
| C | -2.97232000 | 3.93925200 | 1.32651400 |
| C | -1.23911300 | 2.15206400 | -1.42946500 |
| C | 0.18573000 | 2.57283000 | -1.84186900 |
| C | -2.26643500 | 3.18928900 | -1.90282500 |
| C | -1.56463000 | 0.79106200 | -2.07350900 |
| C | 1.49349000 | -1.02152300 | -0.60065600 |
| C | 1.40609700 | -1.09430600 | -1.98621700 |
| C | 0.48552100 | -1.90469500 | -2.64401300 |
| C | -0.41431200 | -2.65762700 | -1.90711000 |
| C | -0.38799900 | -2.59310400 | -0.51974500 |
| C | 0.57003800 | -1.80072800 | 0.09356600 |
| C | 3.47852700 | 0.93017900 | -0.52909300 |
| C | 4.48139900 | 0.54304400 | -1.41285600 |
| C | 5.29476200 | 1.44536200 | -2.08155800 |
| C | 5.12363100 | 2.80704400 | -1.85959700 |
| C | 4.15091800 | 3.23801700 | -0.97023400 |
| C | 3.36257400 | 2.29763600 | -0.31801800 |
| C | 3.53918900 | -1.12150900 | 1.18029900 |
| C | 3.90320100 | -2.42742700 | 0.86837000 |
| C | 4.76125700 | -3.17770500 | 1.66477500 |
| C | 5.29003300 | -2.61886300 | 2.81997600 |
| C | 4.95717200 | -1.31554100 | 3.16646600 |
| C | 4.10195300 | -0.60054000 | 2.34122600 |
| B | 2.60531700 | -0.19187400 | 0.23385200 |
| H | -3.82671800 | -1.92772400 | 1.91947800 |
| H | -1.38335500 | -0.60800700 | 2.00662300 |
| H | -7.11303800 | -2.80707300 | -1.87717100 |
| H | -9.12786500 | -1.81980000 | -0.85291100 |
| H | -8.92302400 | -0.08453700 | 0.88205300 |
| H | -3.60941500 | -1.77386000 | -0.94535800 |
| H | -4.53527400 | -0.86762200 | -3.07143900 |
| H | -5.30587400 | -2.42744100 | -3.40337500 |
| H | -3.53941900 | -2.31951300 | -3.31921500 |
| H | -5.25032400 | -4.28567700 | -1.53266800 |
| H | -4.40113100 | -3.94098100 | -0.01706100 |
| H | -3.48233600 | -4.15023000 | -1.51652500 |
| H | -5.63832900 | 0.93013400 | 2.36823000 |
| H | -7.68026100 | 2.54603000 | 0.76271600 |
| H | -5.92844500 | 2.48442300 | 0.46703800 |
| H | -6.55742100 | 3.20166900 | 1.97183600 |
| H | -7.64942900 | -0.07970700 | 3.50610700 |
| H | -8.67591300 | 1.12328400 | 2.70384000 |
| H | -7.42771700 | 1.65058600 | 3.83062500 |
| H | -2.02926100 | 2.14581700 | 3.19383300 |
| H | -1.45676900 | 3.77689600 | 3.57174700 |
| H | -0.30081000 | 2.53491900 | 3.04680200 |
| H | -0.59783000 | 5.29139700 | 1.82790900 |
| H | -0.69177200 | 4.90690500 | 0.10771800 |
| H | 0.51146200 | 4.09787900 | 1.13537400 |
| H | -3.18292700 | 4.34682700 | 0.33590500 |
| H | -3.08979600 | 4.75176600 | 2.05337000 |
| H | -3.72334500 | 3.17508300 | 1.54197300 |
| H | 0.53616600 | 3.46923900 | -1.32257100 |
| H | 0.18055000 | 2.78806300 | -2.91624000 |
| H | 0.90750300 | 1.77163200 | -1.66654600 |
| H | -3.27076600 | 2.93173100 | -1.55502700 |
| H | -2.27168100 | 3.18956700 | -2.99923400 |
| H | -2.01423500 | 4.20355300 | -1.57788700 |
| H | -1.26182900 | 0.81355700 | -3.12646900 |
| H | -2.63455700 | 0.57763300 | -2.01746500 |
| H | -1.02609600 | -0.02401200 | -1.58606000 |
| H | 1.38247700 | 0.64526600 | 0.86979300 |
| H | 2.10083300 | 0.46291400 | 1.27862800 |

**Supplementary Table 28.** Computed Cartesian coordinates (x, y, z) for the optimized structure of **TS6a**.

E1 = -3633.302927, G1 = -3632.649794, E2 = -3634.237746

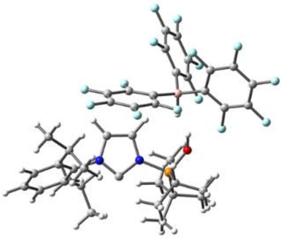

| | | | | | | | |
|---|---|---|---|---|---|---|---|
| P | -1.15094300 | 2.61305600 | -0.57380700 | C | 5.77212100 | -0.20994200 | -1.36001000 |
| F | 0.17673000 | -2.13481700 | 1.41478300 | C | 5.90218200 | 1.10262100 | -1.79651400 |
| F | -1.18357400 | -4.04729900 | 0.19825000 | C | 4.84089200 | 1.97903000 | -1.63239600 |
| F | -0.82761900 | -4.52697600 | -2.47699500 | C | 3.67573900 | 1.52256800 | -1.02833100 |
| F | 0.92433900 | -2.99327700 | -3.88231500 | C | 2.48554900 | -0.48621500 | 1.75820200 |
| F | 2.30015300 | -1.07156400 | -2.69563600 | C | 2.87556300 | -1.68329500 | 2.35282700 |
| F | 2.68119900 | 2.43876000 | -0.91681500 | C | 3.22357800 | -1.79045900 | 3.69403000 |
| F | 4.94254600 | 3.24253100 | -2.05285100 | C | 3.19588700 | -0.66200300 | 4.50222300 |
| F | 7.03175900 | 1.51273600 | -2.37058300 | C | 2.81600300 | 0.55483500 | 3.95580500 |
| F | 6.78519200 | -1.06158700 | -1.51867100 | C | 2.47755400 | 0.61348300 | 2.60979000 |
| F | 4.50917200 | -1.89426700 | -0.37621200 | B | 2.13946200 | -0.28793300 | 0.17757900 |
| F | 2.12313000 | 1.82908300 | 2.14070400 | H | -2.56302200 | -1.43290300 | 1.75435900 |
| F | 2.77931500 | 1.64897900 | 4.72058100 | H | -0.48014600 | 0.33111300 | 1.37594000 |
| F | 3.52624900 | -0.74751600 | 5.78985700 | H | -6.44929700 | -3.39672200 | -1.04996700 |
| F | 3.58061500 | -2.96548200 | 4.21381100 | H | -8.33110500 | -2.33205500 | 0.12063000 |
| F | 2.91116900 | -2.81577500 | 1.64093600 | H | -7.98226900 | -0.28230800 | 1.44205700 |
| O | 0.19897500 | 2.72124100 | 0.22591700 | H | -2.93391500 | -2.41949900 | -0.58204500 |
| N | -3.54123000 | -0.12234600 | 0.32106700 | H | -3.42275900 | -0.80251000 | -2.41413000 |
| N | -1.93871100 | 1.22896200 | 0.00555400 | H | -4.45838200 | -2.00731800 | -3.19835100 |
| C | -3.23866500 | 0.98077200 | -0.39755800 | H | -2.71742600 | -2.31405900 | -3.03489200 |
| C | -2.49344900 | -0.55014000 | 1.13871800 | H | -4.78430500 | -4.31619200 | -2.09581900 |
| C | -1.47252300 | 0.30602500 | 0.95528500 | H | -4.22342500 | -4.57677500 | -0.42961600 |
| C | -4.83826000 | -0.74510600 | 0.28783500 | H | -3.06285000 | -4.49888100 | -1.76214300 |
| C | -5.00037100 | -1.92767500 | -0.44966100 | H | -4.60055100 | 1.24066900 | 1.99216300 |
| C | -6.27942100 | -2.48228400 | -0.49257000 | H | -7.22816900 | 2.29317000 | 0.82853800 |
| C | -7.34283600 | -1.88523300 | 0.17401900 | H | -5.63914700 | 2.39183900 | 0.04648200 |
| C | -7.14435900 | -0.73055600 | 0.91712100 | H | -5.94662600 | 3.27698900 | 1.56193700 |
| C | -5.88182100 | -0.14103700 | 1.00155800 | H | -6.04453400 | 0.17546600 | 3.74418400 |
| C | -3.83299000 | -2.56692700 | -1.18868000 | H | -7.44169300 | 1.06218800 | 3.10921900 |
| C | -3.59179500 | -1.87660100 | -2.54010900 | H | -6.07176800 | 1.94960000 | 3.77802600 |
| C | -3.99282200 | -4.07791500 | -1.37620900 | H | -3.32657000 | 3.03901900 | 1.44334600 |
| C | -5.67535600 | 1.12799200 | 1.81336900 | H | -2.96448500 | 4.72264900 | 1.85632900 |
| C | -6.14911500 | 2.34936000 | 1.01327500 | H | -1.72478600 | 3.48008800 | 2.08707100 |
| C | -6.34920000 | 1.06845300 | 3.18928700 | H | -1.65229800 | 6.17944900 | 0.37556300 |
| C | -2.06636900 | 4.10725200 | 0.00369200 | H | -0.67746400 | 5.53461200 | -0.94762200 |
| C | -2.54447200 | 3.80206900 | 1.43721600 | H | -0.26461500 | 5.13205000 | 0.72889800 |
| C | -1.09208400 | 5.30134900 | 0.03647800 | H | -2.95968400 | 4.84576400 | -1.85312300 |
| C | -3.27224500 | 4.42344000 | -0.89354200 | H | -3.88657900 | 5.17543200 | -0.38665600 |
| C | -0.83051800 | 2.38450900 | -2.37647800 | H | -3.88856100 | 3.53811800 | -1.07159200 |
| C | 0.04376800 | 3.55460600 | -2.86385000 | H | -0.49915900 | 4.50488500 | -2.85429900 |
| C | -2.12470200 | 2.30156000 | -3.20469200 | H | 0.32694100 | 3.35217800 | -3.90221900 |
| C | -0.04802300 | 1.06396200 | -2.51397400 | H | 0.96381700 | 3.65474800 | -2.28199300 |
| C | 1.37143300 | -1.53038700 | -0.55282000 | H | -2.82888500 | 1.57082700 | -2.80018700 |
| C | 1.48978900 | -1.80470300 | -1.91373700 | H | -1.84701800 | 1.99850800 | -4.21991600 |
| C | 0.76982600 | -2.79514700 | -2.57227200 | H | -2.63286700 | 3.26534500 | -3.27621000 |
| C | -0.12701700 | -3.57314200 | -1.85846400 | H | 0.13176000 | 0.88509500 | -3.57889200 |
| C | -0.29826200 | -3.33136900 | -0.50430500 | H | -0.60318500 | 0.20700000 | -2.12032400 |
| C | 0.43661800 | -2.32508200 | 0.10703400 | H | 0.92604000 | 1.10739700 | -2.02487300 |
| C | 3.49133200 | 0.22443200 | -0.57704800 | H | 0.86326900 | 1.98622200 | 0.15864400 |
| C | 4.58521200 | -0.61566400 | -0.76567700 | H | 1.31873700 | 0.62003700 | 0.11425800 |

**Supplementary Table 29.** Computed Cartesian coordinates (x, y, z) for the optimized structure of **8aB$^{1}$**.

## Supplementary References


1. Hoshimoto, Y., Kinoshita, T., Ohashi, M. & Ogoshi, S. A Strategy to Control the Reactivation of Frustrated Lewis Pairs from Shelf-Stable Carbene Borane Complexes. *Angew. Chem. Int. Ed.* **54**, 11666–11671 (2015).
2. Ullrich, M., Lough, A. J. & Stephan, D. W. Reversible, Metal-Free, Heterolytic Activation of $H_2$ at Room Temperature. *J. Am. Chem. Soc.* **131**, 52–53 (2009).
3. Ullrich, M., Lough, A. J. & Stephan, D. W. Dihydrogen Activation by $B(p\text{-}C_6F_4H)_3$ and Phosphines. *Organometallics* **29**, 3647–3654 (2010).
4. Chai, J.-D. & Head-Gordon, M. Long-range corrected hybrid density functionals with damped atom–atom dispersion corrections. *Phys. Chem. Chem. Phys.* **10**, 6615–6620 (2008)
5. Hehre, W. J., Ditchfield, R. & Pople, J. A. Self-Consistent Molecular Orbital Methods. XII. Further Extensions of Gaussian-Type Basis Sets for Use in Molecular Orbital Studies of Organic Molecules. *J. Chem. Phys* **56**, 2257–2261 (1972).
6. Hariharan, P. C. & Pople, J. A. *The Influence of Polarization Functions on Molecular Orbital Hydrogenation Energies*. *Theor. Chim. Acta.* 28 213–222 (1973).
7. Tomasi, J., Mennucci, B. & Cammi, R. Quantum Mechanical Continuum Solvation Models. *Chem. Rev.* **105**, 2999–3094 (2005).
8. Frisch, M. J., Trucks, G. W., Schlegel, H. B., Scuseria, G. E., Robb, M. A., Cheeseman, J. R., Scalmani, G., Barone, V., Mennucci, B., Petersson, G. A., Nakatsuji, H., Caricato, M., Li, X., Hratchian, H. P., Izmaylov, A. F., Bloino, J., Zheng, G., Sonnenberg, J. L., Hada, M., Ehara, M., Toyota, K., Fukuda, R., Hasegawa, J., Ishida, M., Nakajima, T., Honda, Y., Kitao, O., Nakai, H., Vreven, T., Montgomery, J. A., Peralta, Jr., J. E., Ogliaro, F., Bearpark, M., Heyd, J. J., Brothers, E., Kudin, K. N., Staroverov, R. Kobayashi, J. Normand, K. Raghavachari, A. Rendell, J. C. Burant, S. S. Iyengar, V. N., Tomasi, J., Cossi, M., Rega, N., Millam, J. M., Klene, M., Knox, J. E., Cross, J. B., Bakken, V., Adamo, C., Jaramillo, J., Gomperts, R., Stratmann, R. E., Yazyev, O., Austin, A. J., Cammi, R., Pomelli, C., Ochterski, J. W., Martin, R. L., Morokuma, K., Zakrzewski, V. G., Voth, G. A., Salvador, P., Dannenberg, J. J., Dapprich, S., Daniels, A. D., Farkas, Ö., Foresman, J. B., Ortiz, J. V., Cioslowski, J. & Fox, D. J., Gaussian 09, Revision C.02. Gaussian, Inc., Wallingford CT (2009).
9. Keith, T. A. TK Gristmill Software, Overland Park KS, USA (2019) (aim.tkgristmill.com).

**Supplementary Figure 38.** $^{1}$H NMR spectrum of **3aB$^{2}$** (400 MHz, $CD_2Cl_2$).

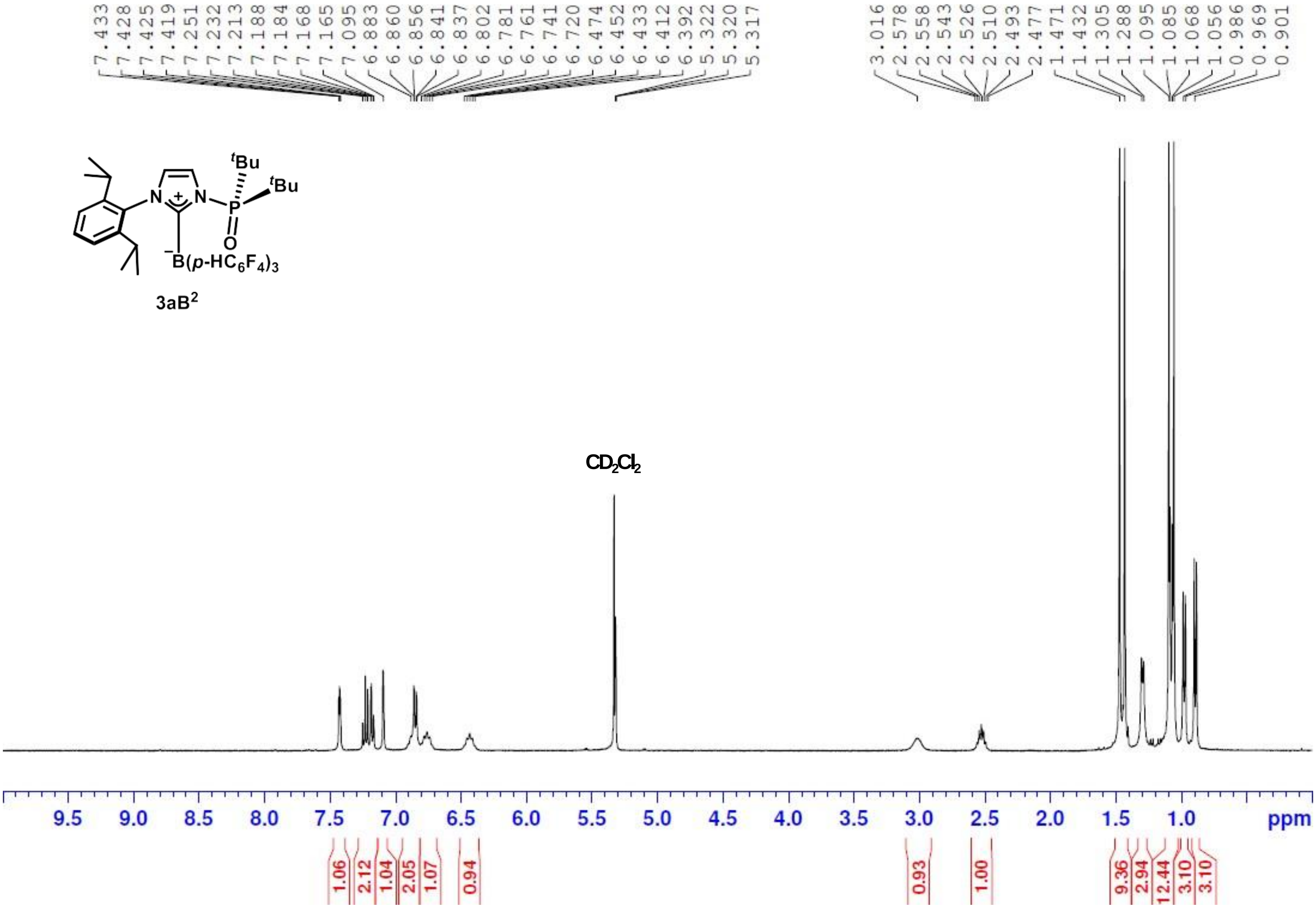

**Supplementary Figure 39.** $^{11}$**B NMR spectrum of 3aB**$^{2}$ **(128 MHz,** $CD_2Cl_2$**).**

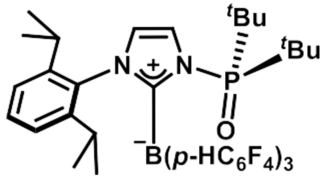


3aB$^{2}$

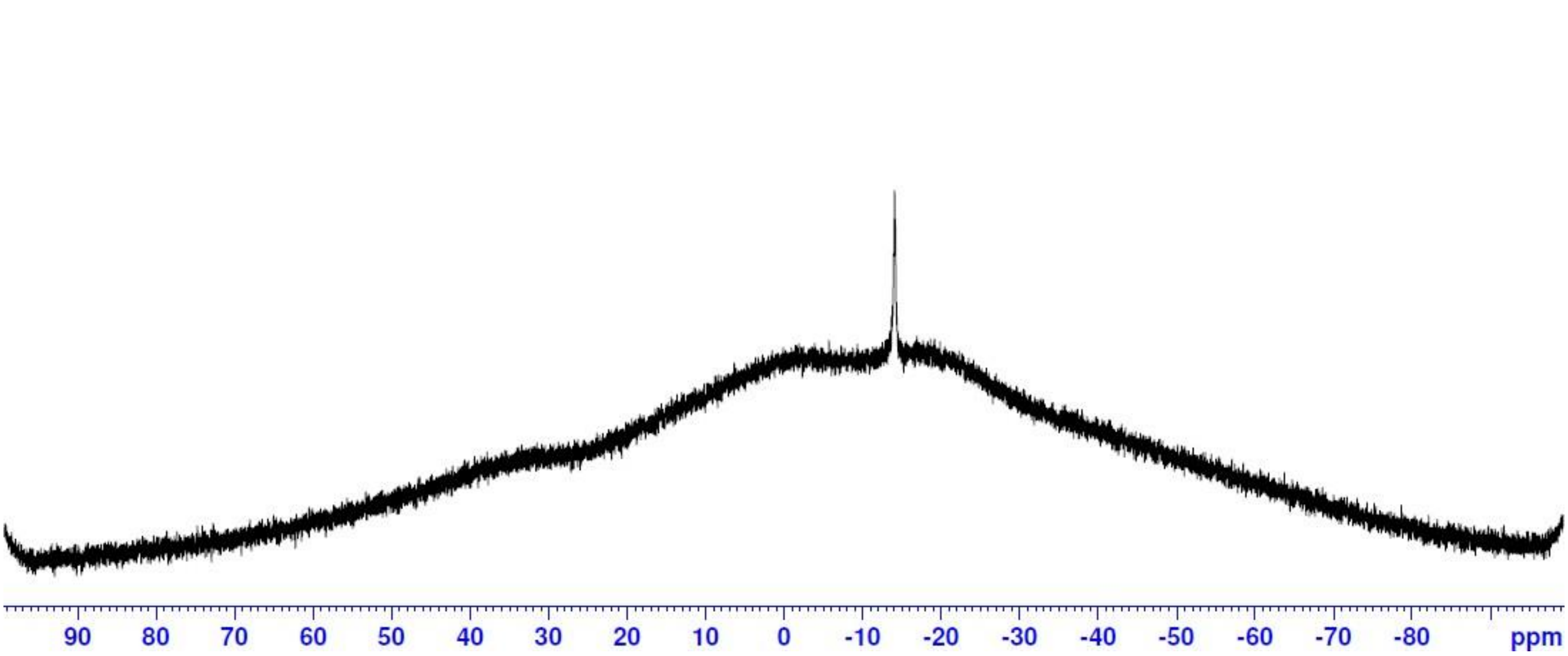

**Supplementary Figure 40.** $^{13}$**C NMR spectrum of 3aB**$^2$ **(100 MHz,** $CD_2Cl_2$**).**

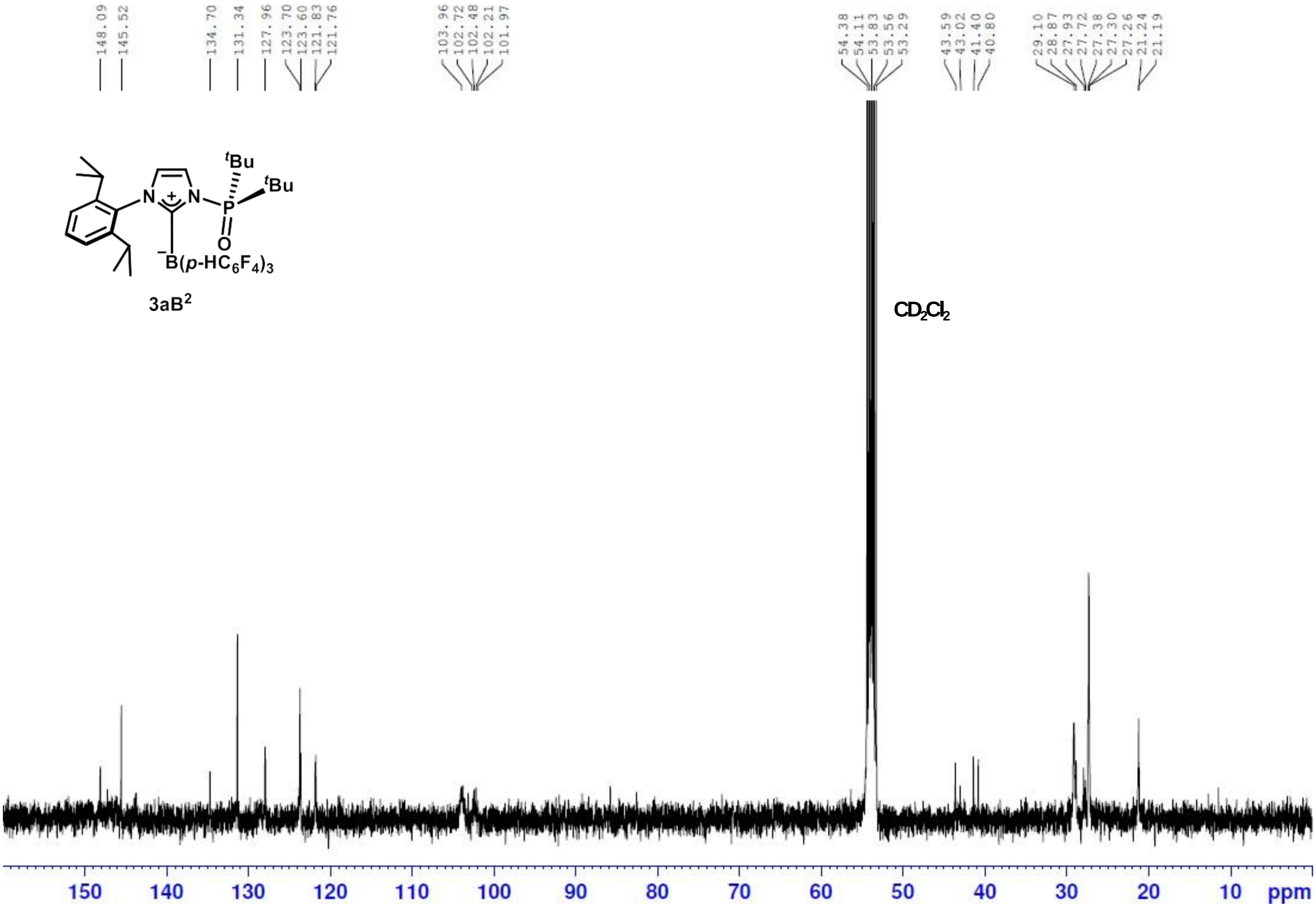

**Supplementary Figure 41.** **$^{19}F$ NMR spectrum of $3aB^2$ (376 MHz, $CD_2Cl_2$).**

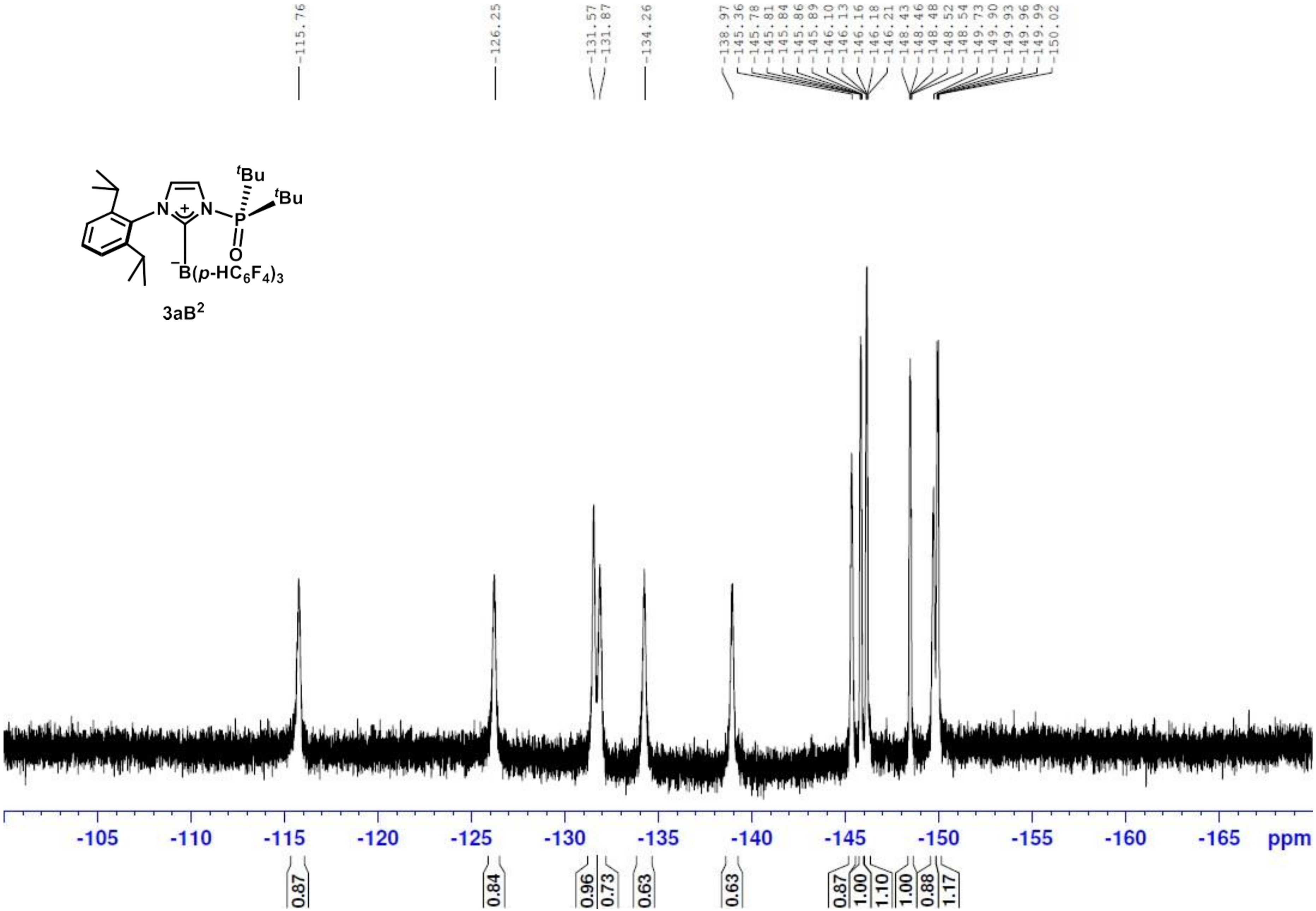

**Supplementary Figure 42. $^{31}$P NMR spectrum of 3aB$^{2}$ (162 MHz, $CD_2Cl_2$).**

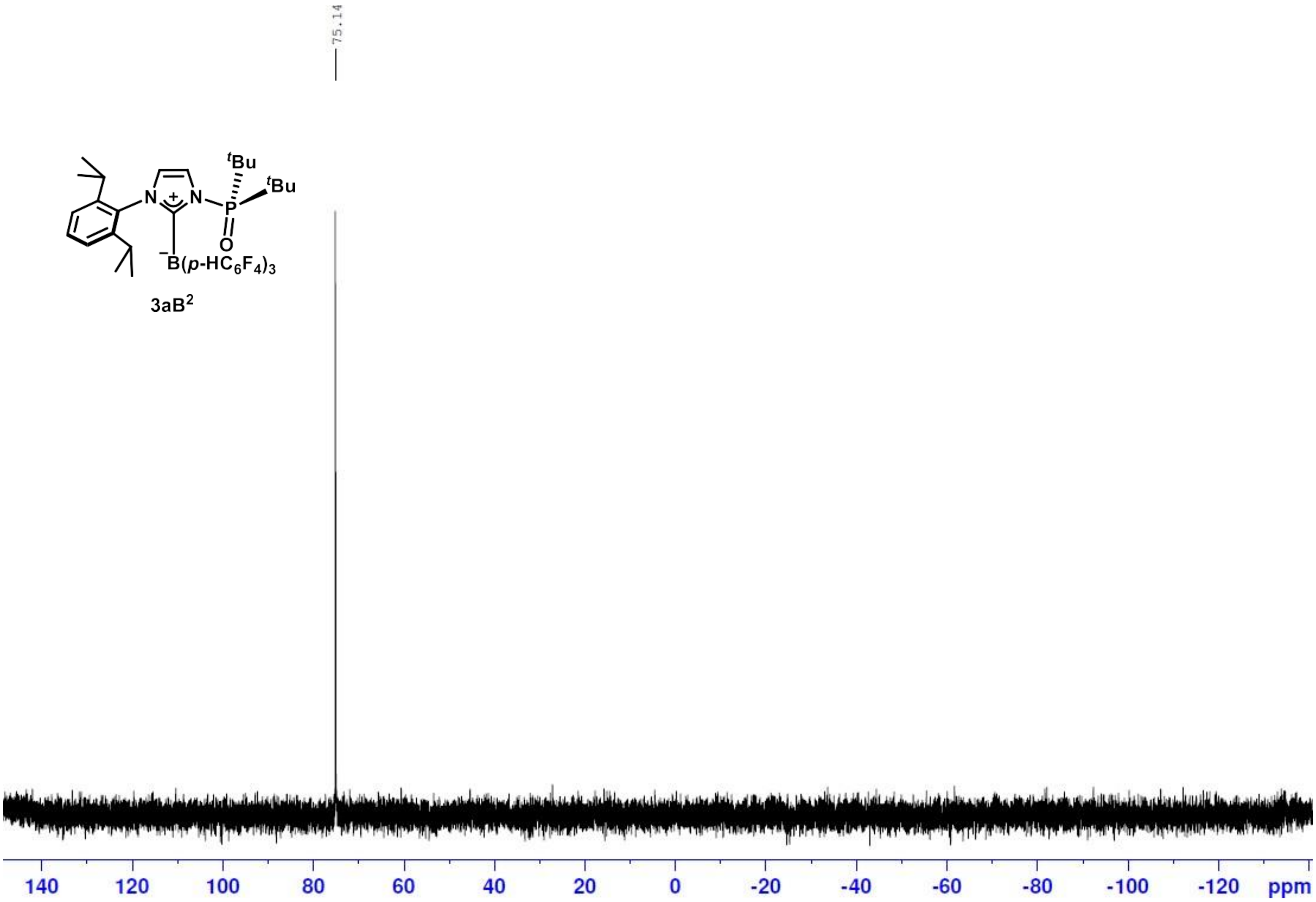

Supplementary Figure 43. $^1$H NMR spectrum of 5aB$^2$ (400 MHz, $CD_2Cl_2$).

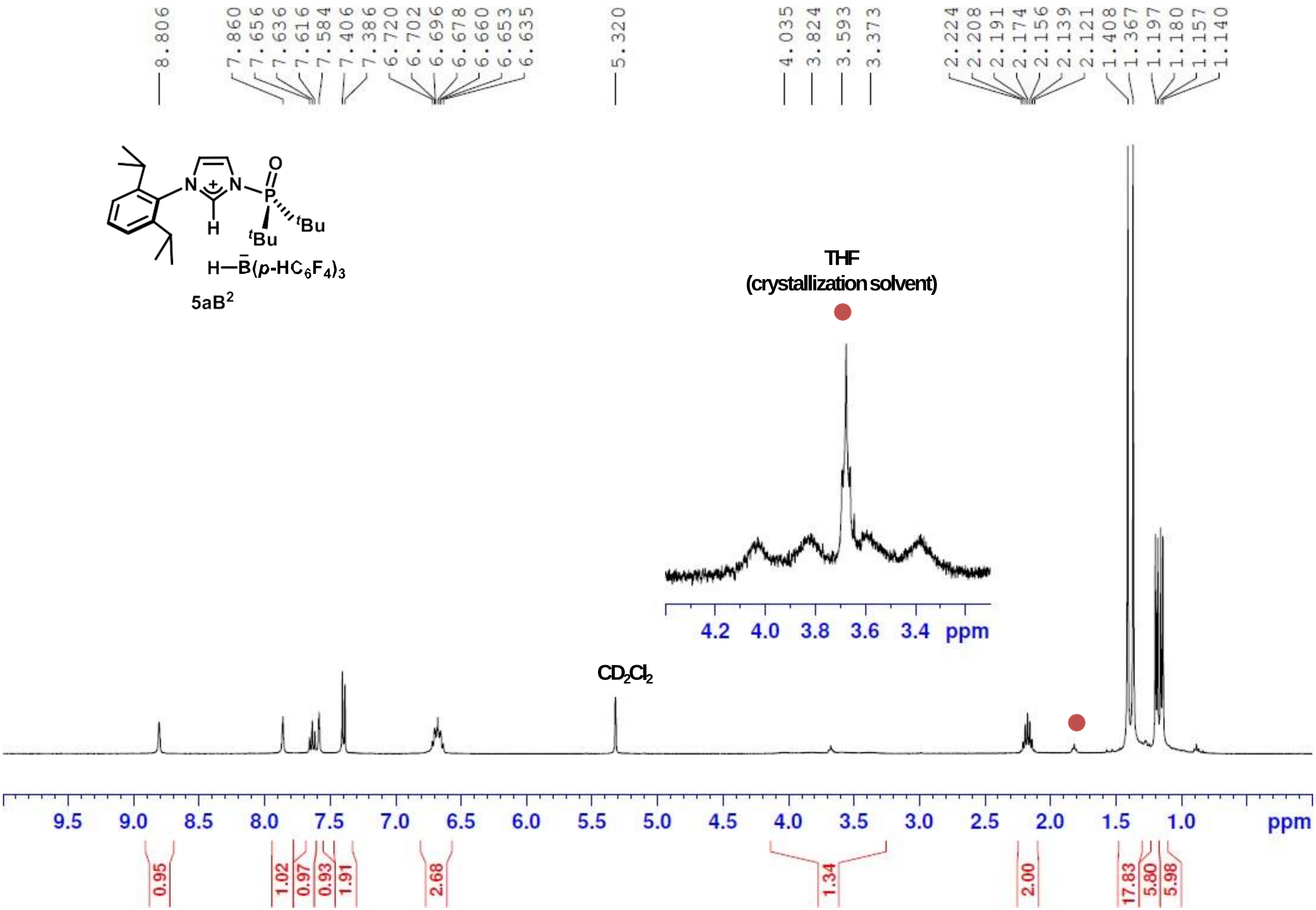

**Supplementary Figure 44.** $^{11}B$ **NMR spectrum of 5aB² (128 MHz, $CD_2Cl_2$).**

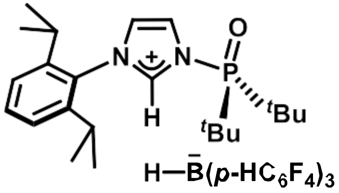


5aB²

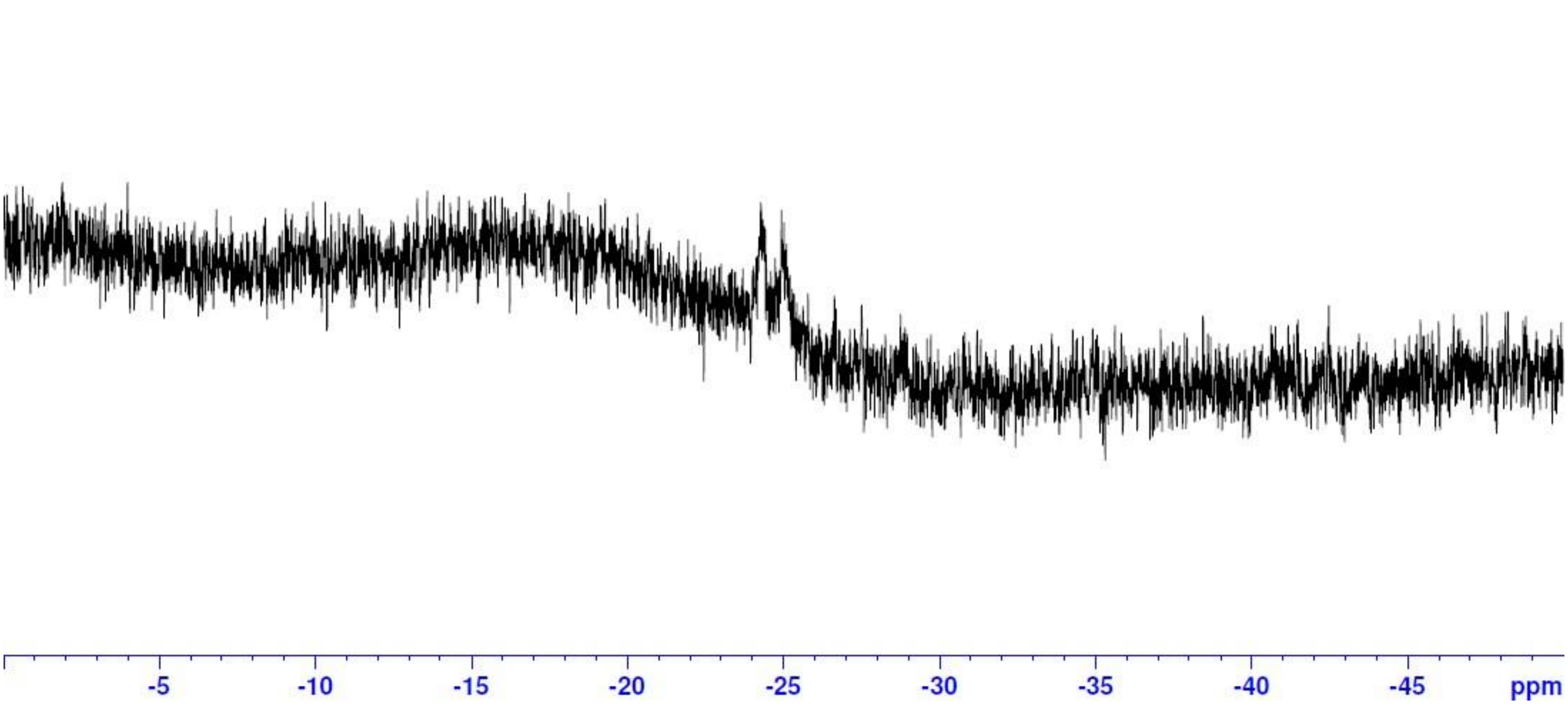

**Supplementary Figure 45.** $^{13}C$ **NMR spectrum of 5aB² (100 MHz,** $CD_2Cl_2$**).**

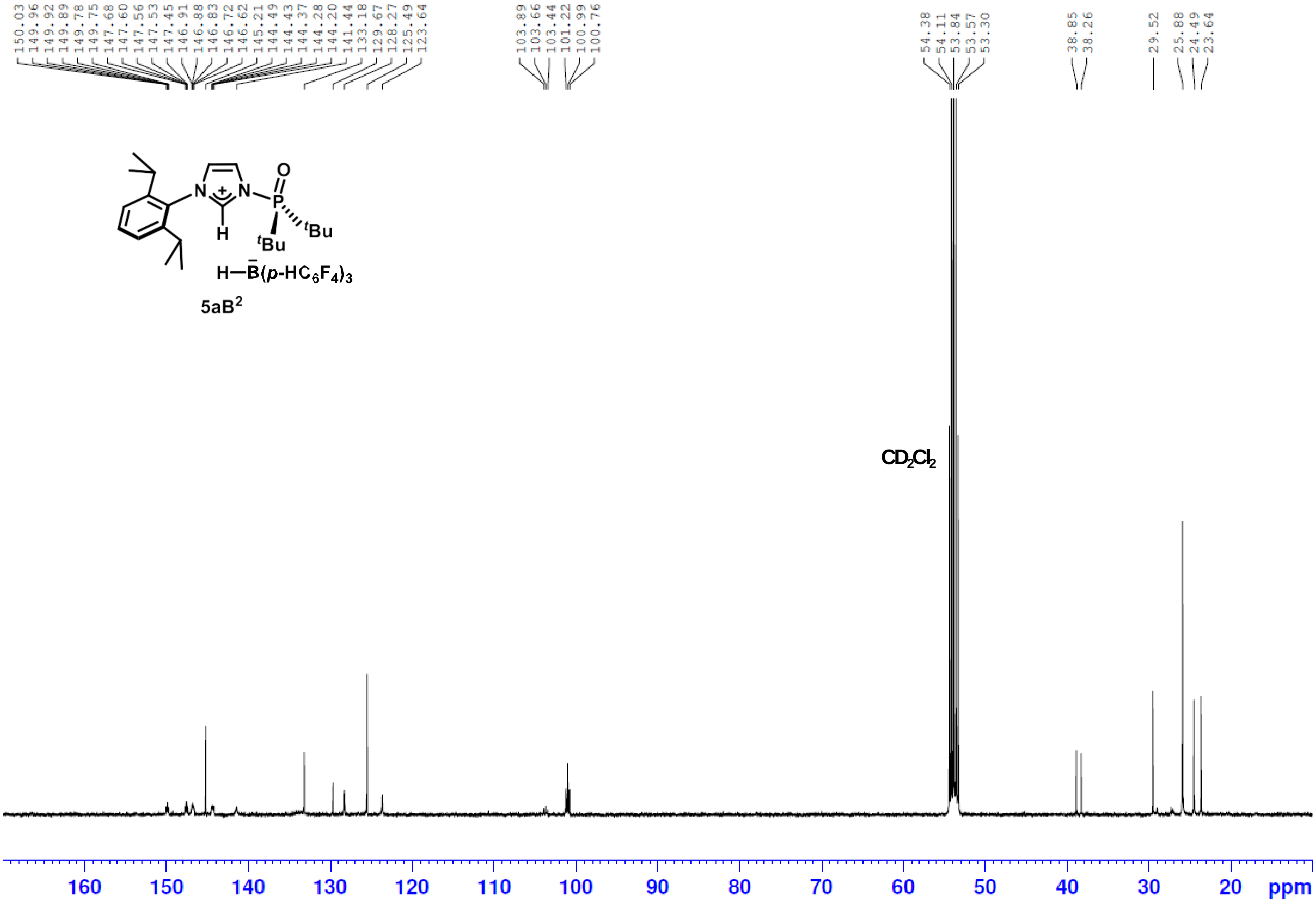

**Supplementary Figure 46.** $^{19}$**F NMR spectrum of 5aB**$^{2}$ **(376 MHz,** $CD_2Cl_2$**).**

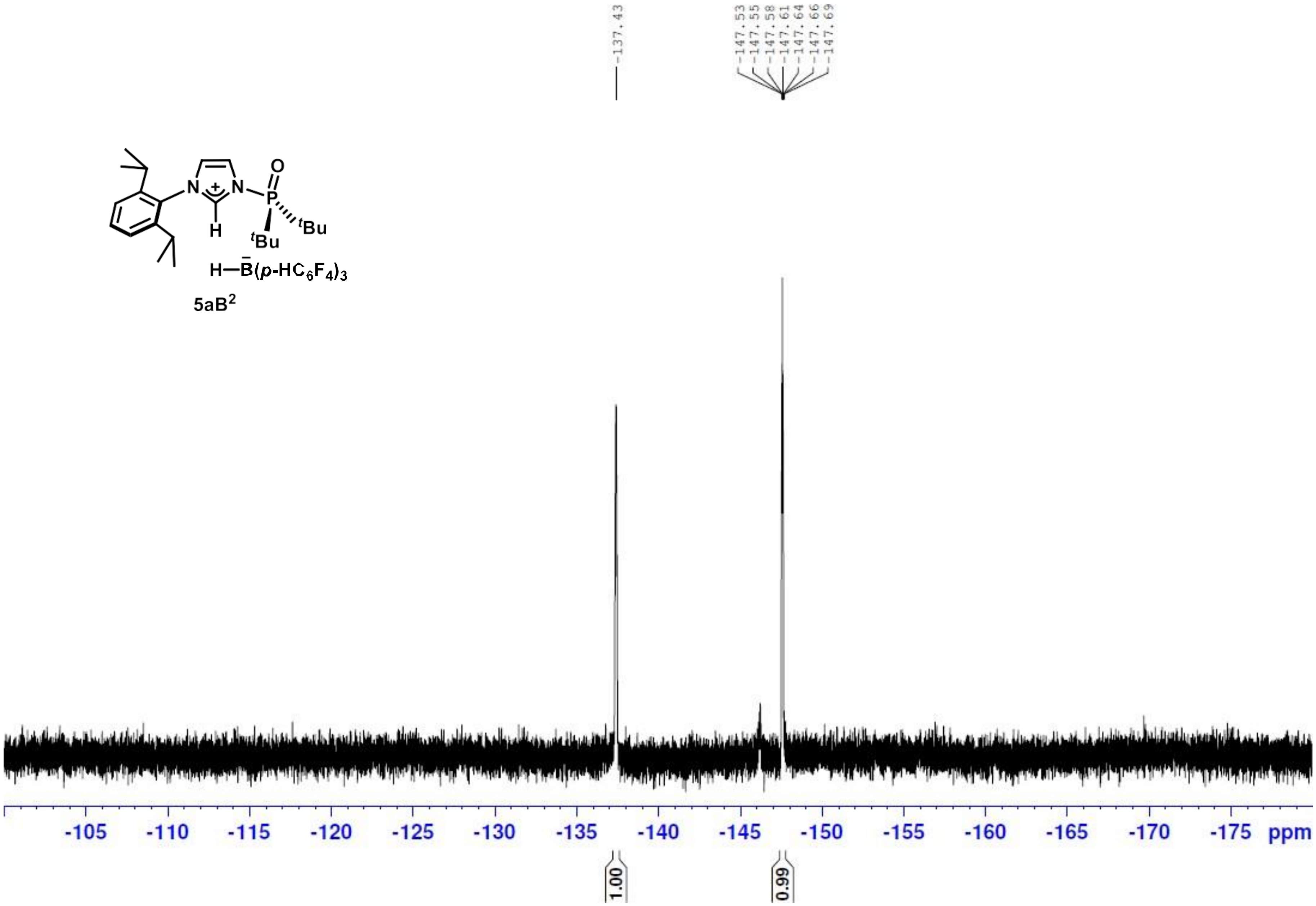

Supplementary Figure 47. $^{31}P$ NMR spectrum of $5aB^2$ (162 MHz, $CD_2Cl_2$).

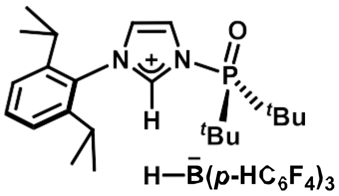


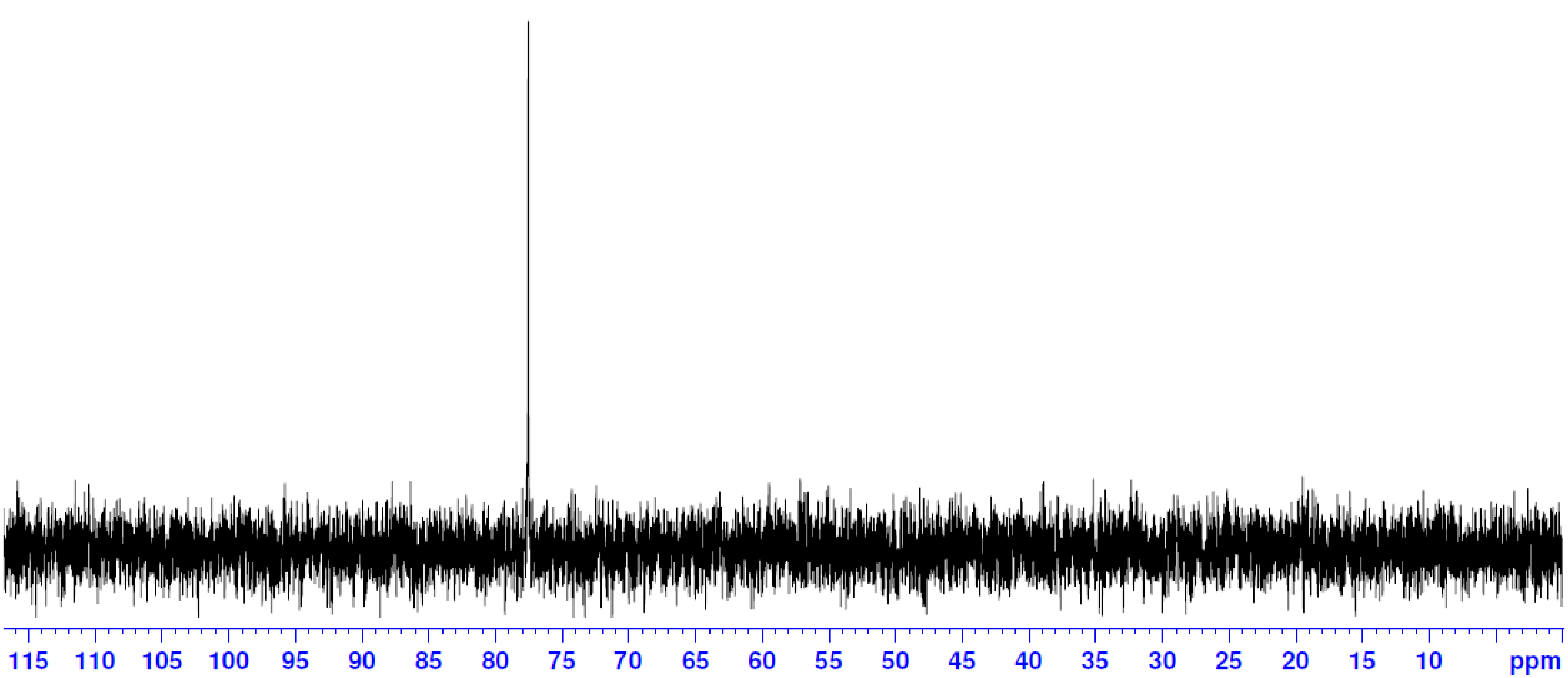

**Supplementary Figure 48.** $^1$H NMR spectrum of [1a–H][HO(B$^2$)$_2$] (400 MHz, $CD_2Cl_2$).

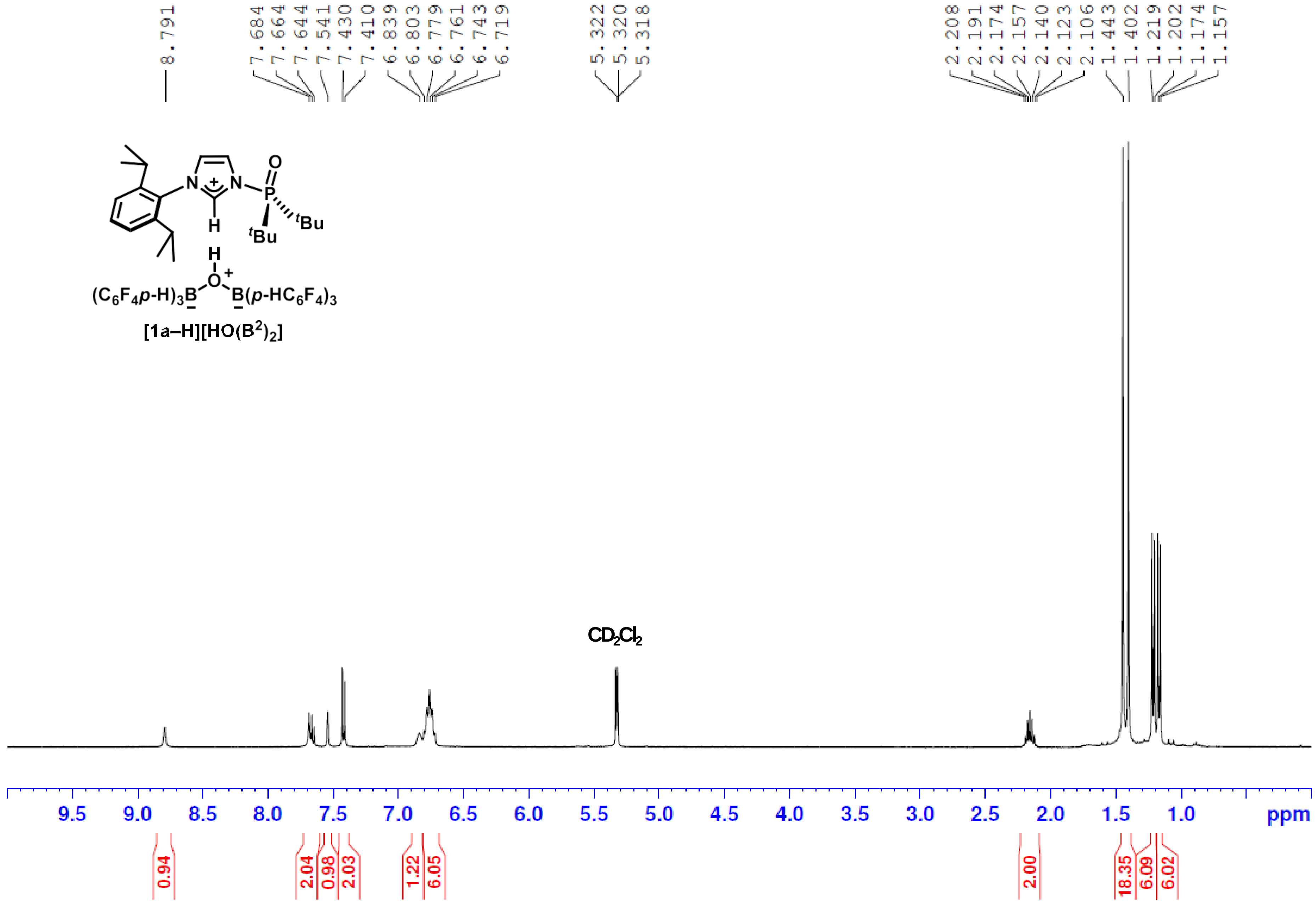

**Supplementary Figure 49.** $^{11}$**B NMR spectrum of [1a–H][HO(B$^2$)$_2$] (128 MHz, CD$_2$Cl$_2$).**

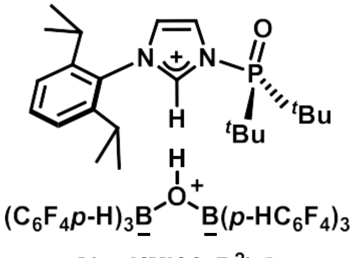


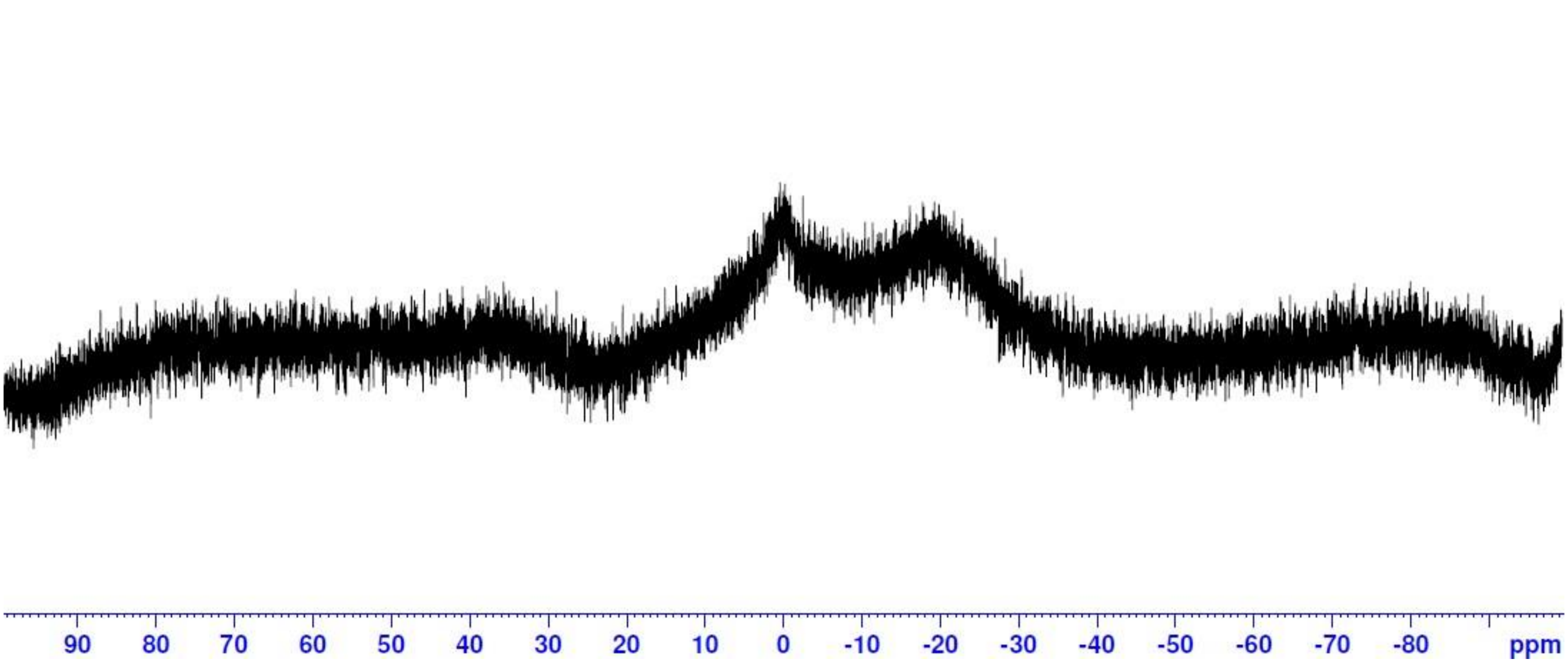

**Supplementary Figure 50.** $^{13}$C NMR spectrum of [1a–H][HO(B$^2$)$_2$] (150 MHz, $CD_2Cl_2$).

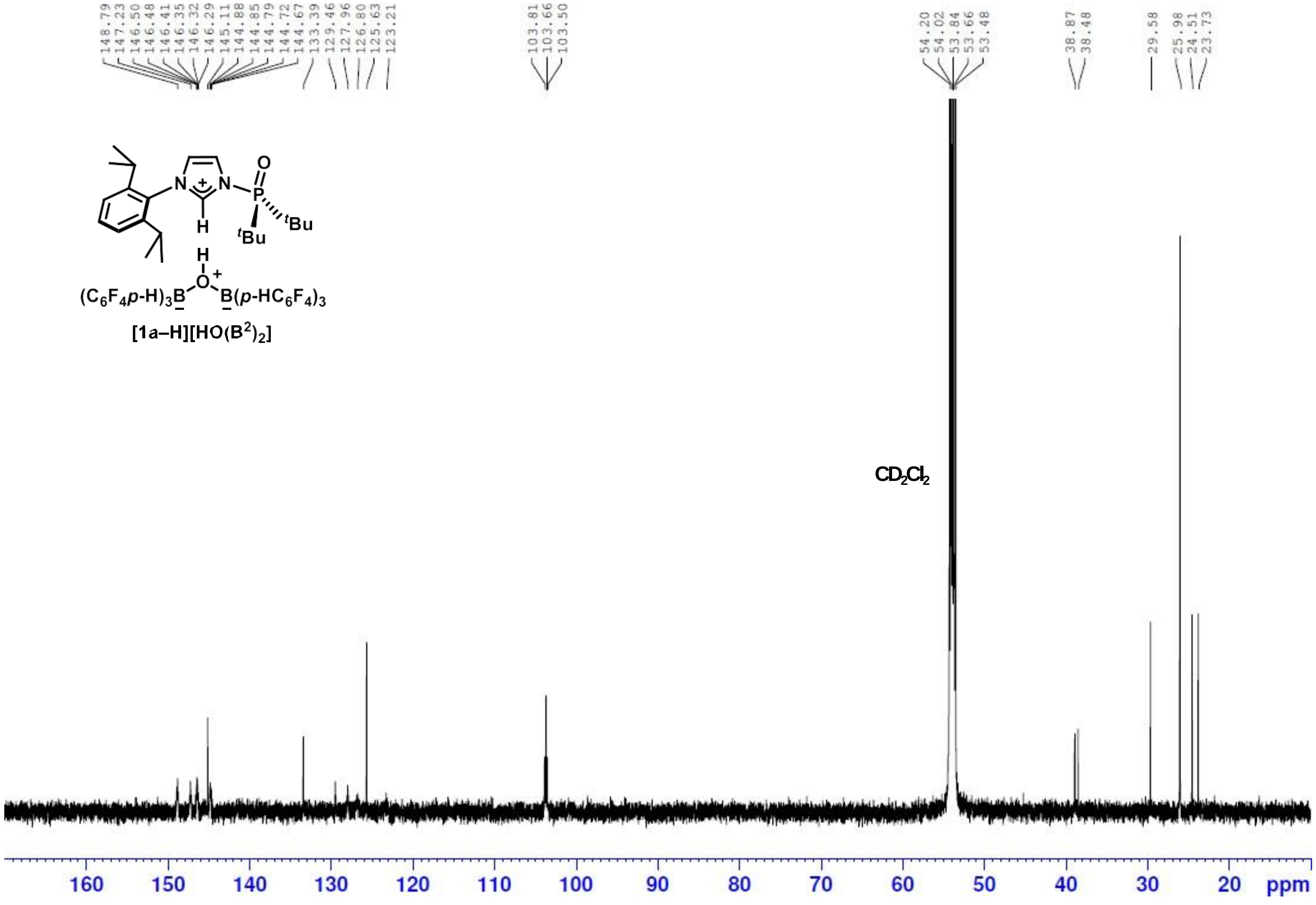

**Supplementary Figure 51.** $^{19}$**F NMR spectrum of [1a–H][HO(B$^2$)$_2$] (376 MHz, $CD_2Cl_2$).**

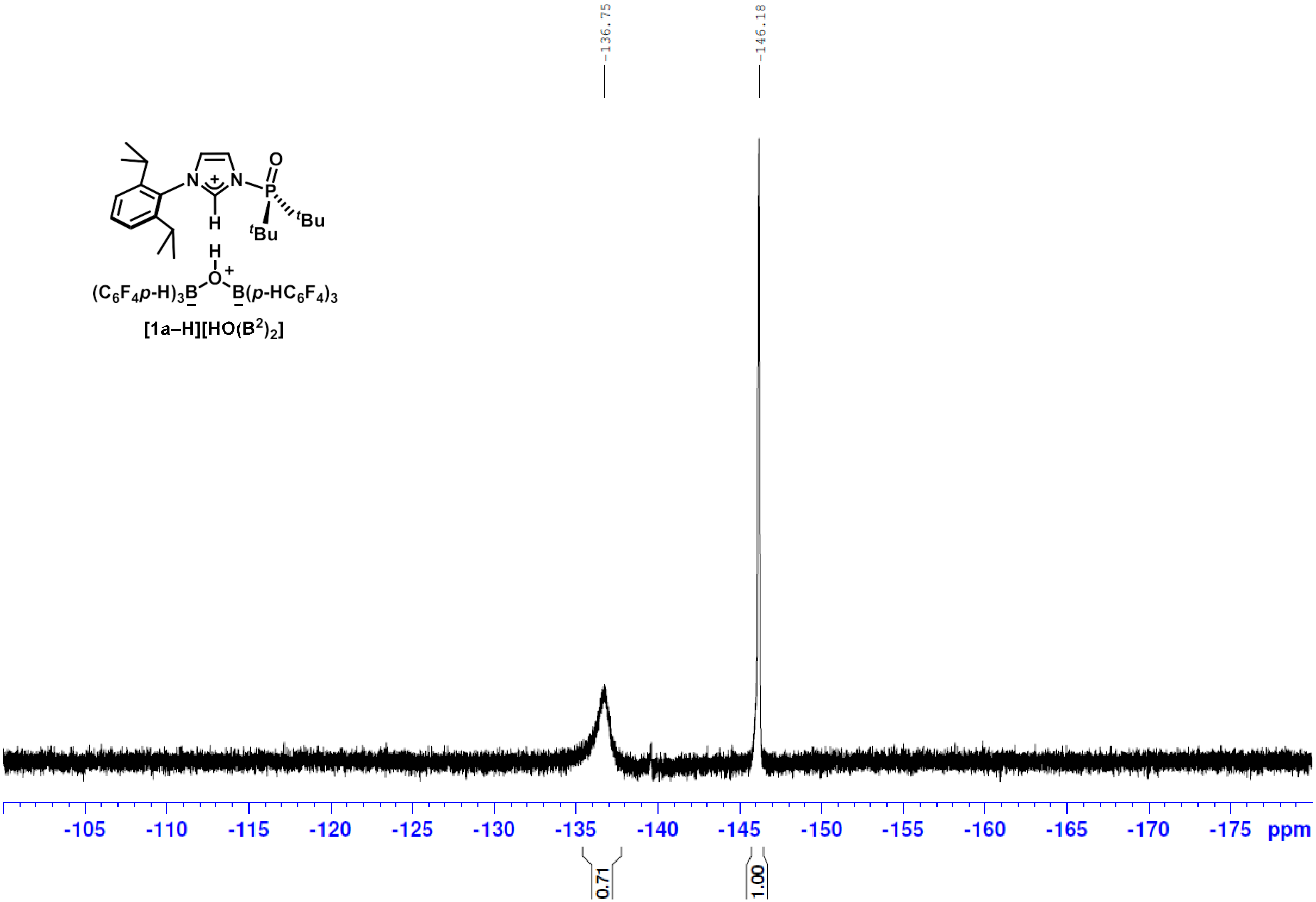

**Supplementary Figure 52.** $^{31}$**P NMR spectrum of [1a–H][HO(B$^2$)$_2$] (162 MHz, $CD_2Cl_2$).**

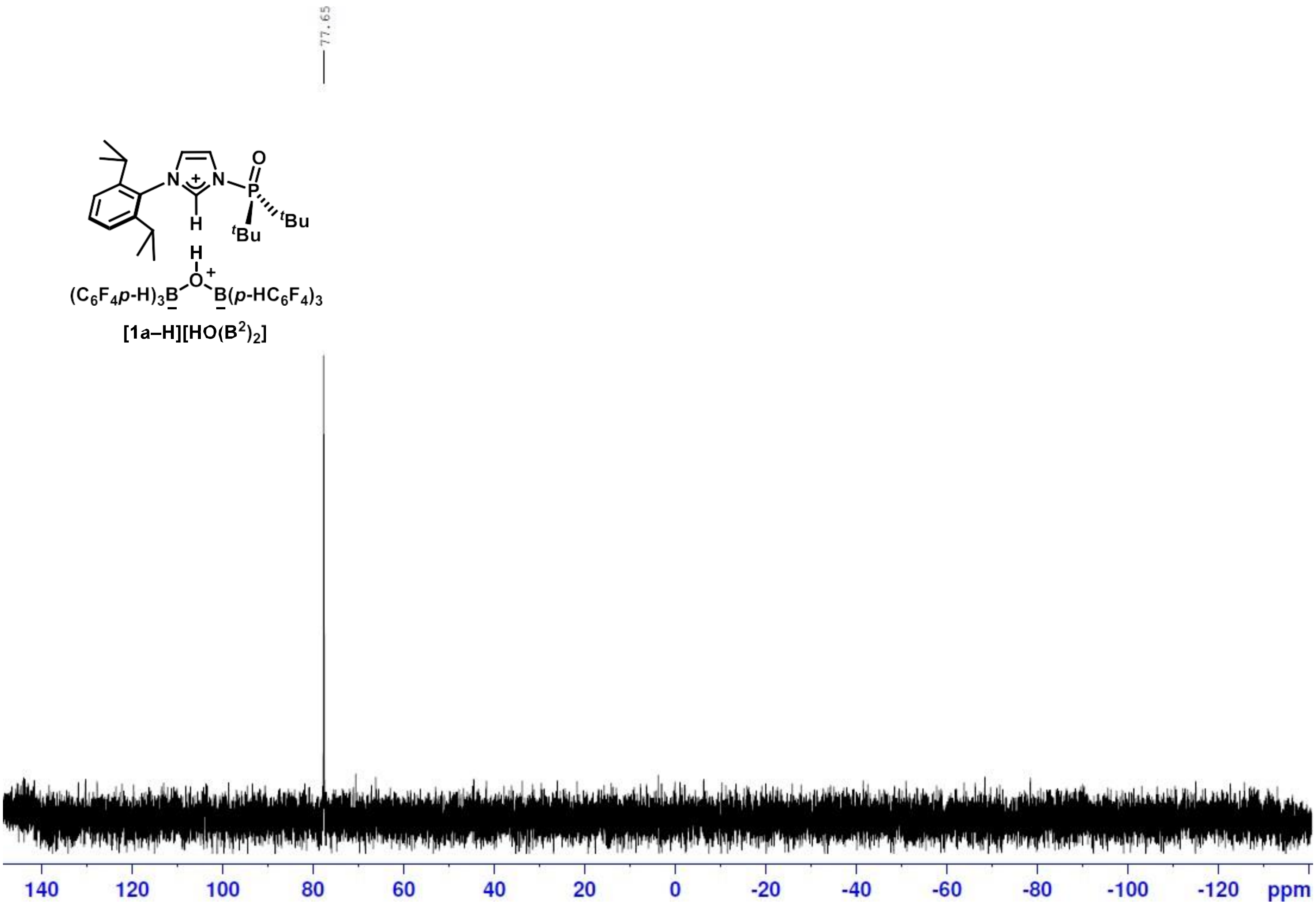

Supplement: Supplementary file 1 — Supplementary Materials [file 42004_2021_576_MOESM1_ESM.pdf]
