# Supplementary material for: A boron-transfer mechanism mediating the thermally induced revival of frustrated carbene–borane pairs from their shelf-stable adducts
Source: Commun Chem. 2021 Sep 27;4:137. doi: 10.1038/s42004-021-00576-1 (PMC9814311; doi:10.1038/s42004-021-00576-1)
Supplement: Supplementary file 2 — Description of Additional Supplementary Files [file 42004_2021_576_MOESM2_ESM.pdf]

# Description of Additional Supplementary Files

**File Name:** Supplementary Data 1

**Description:** Results of AIM Analysis

**File Name:** Supplementary Data 2

**Description:** CIF for Compound 3aB$^2$

**File Name:** Supplementary Data 3

**Description:** CIF for Compound 5aB$^2$

**File Name:** Supplementary Data 4

**Description:** CIF for Compound [1a-H][HO(B$^2$)$_2$]

**File Name:** Supplementary Data 5

**Description:** CIF for Compound 2aB$^2$
